# Feasibility of Accelerometer-Based Arterial Pulse Measurement: Part II—Experimental Investigation

**DOI:** 10.3390/s26216936

**Published:** 2026-11-02

**Authors:** Mamun Hasan, Md Mahfuzur Rahman, Zhili Hao

**Affiliations:** Department of Mechanical and Aerospace Engineering, Old Dominion University, Norfolk, VA 23529, USA; mhasa004@odu.edu (M.H.); mrahm009@odu.edu (M.M.R.)

**Keywords:** arterial pulse measurement, accelerometers, tactile sensors, baseline drift, motion artifacts, time-varying system parameters (TVSP), instantaneous parameters

## Abstract

This study, the second of two parts, experimentally investigates accelerometer (ACC)-based arterial pulse measurement in comparison with tactile-based measurement. Two ACCs, one digital and one analog, were used to measure radial, lateral, and axial arterial wall motions at the carotid artery (CA) in a total of ten subjects. Subsequently, both the analog ACC and a tactile sensor were used to conduct measurements sequentially at the CA of another three subjects. A single-degree-of-freedom time-frequency (SDOF-TF) method was applied to the measured acceleration signals to remove motion artifacts (MA) and sensor noise; reconstruct the pulse waveform in the acceleration domain, *APW_a_*, and displacement domain, as well as APW; and extract arterial indices. The same method was applied to the displacement signals measured by the tactile sensor. The results reveal that the analog ACC acquires higher-quality pulse signals than the digital ACC. Radial wall motion measured by the ACC corresponds well with the pulse signal measured by the tactile sensor, whereas axial wall motion presents substantial practical challenges for reliable measurement for cardiovascular (CV) assessment. Compared with tactile-based measurements, ACC-based measurements present two practical challenges: (1) achieving firm sensor fixation and (2) the acceleration-sensing nature of the ACC, which permits extraction of only *APW_a_* and its associated arterial indices, rather than APW. They also exhibit two fundamental differences: (1) as theoretically identified in Part I, acceleration sensing introduces an additional term into the instantaneous frequency (IF) of individual arterial pulse harmonics, thereby affecting both the IF-based arterial indices and the frequency spectrum of the IF; and (2) variations in the tissue-contact sensor (TCS) stacks contribute to substantial differences in the normalized harmonic amplitude distributions between ACC-based and tactile-based measurements. Overall, ACC-based measurement provides a simple approach for CV assessment; however, tactile-based measurement remains necessary for reliable acquisition of arterial pulse signals and accurate CV indices.

## 1. Introduction

Arterial pulse measurement has been explored primarily using three sensing modalities: photoplethysmography (PPG) sensors, micro/nano-fabricated tactile sensors, and accelerometers (ACCs) [1,2,3,4,5,6,7]. While PPG sensors are widely used for peripheral pulse measurement, tactile sensors and ACCs have been investigated for arterial pulse measurement at the carotid artery (CA), which provides more direct information on central arterial dynamics relevant to cardiovascular (CV) assessment [1,2,8,9,10,11,12,13]. Compared with tactile sensors, ACCs are commercially available, relatively low cost, small in size, and readily integrated into portable systems. In contrast, micro/nano-fabricated tactile sensors for arterial pulse measurement have generally been custom-designed and fabricated in laboratory settings, with relatively limited standardization and commercial availability [14,15,16,17]. These practical advantages make ACCs an attractive alternative for arterial pulse measurement. However, when the objective is to reconstruct the arterial pulse waveform (APW) in displacement, ACC-based measurement has been considerably less investigated. To the best of the authors’ knowledge, only two studies have employed a 3-axis analog accelerometer (LIS344ALH) to measure arterial pulse signals at the CA, followed by double integration of the measured acceleration signals to reconstruct the APW and derive arterial indices [18,19]. The limited use of ACCs for APW reconstruction has been primarily attributed to the challenges of double integration, which can substantially amplify measurement-related errors and distort the reconstructed APW [20,21,22,23,24].

In contrast, there has been increasing interest in directly measuring arterial pulse acceleration waveforms (APW_a_) at the CA using ACCs, thereby avoiding the need for APW reconstruction [25,26,27,28,29,30]. These studies have demonstrated that APW_a_ contains physiological information and can provide clinically relevant arterial indices [27,28,29,30]. Direct analysis of APW_a_ avoids the integration-related errors associated with reconstructing the APW from acceleration, providing a practical alternative to displacement reconstruction. Similarly, ACC-based measurement of cardiac-induced vibrations at the chest, known as seismocardiography (SCG), has demonstrated the physiological and clinical value of directly measured acceleration signals [31,32,33,34,35,36]. Together, these studies demonstrate the potential of directly measured acceleration signals for providing physiologically relevant information.

However, it remains unknown whether the arterial indices derived from ACC-based pulse measurements are consistent with those derived from tactile-based pulse measurements under the same measurement conditions. This comparison is important because the measurement-related factors, collectively quantified by the tissue-contact-sensor (TCS) stack detailed in Part I, differ in their quantitative characteristics between ACC-based and tactile-based measurements and may interact differently with MA. Consequently, differences in the extracted arterial indices between the two sensing modalities may arise not only from differences in MA and TCS stacks but also from fundamental differences between acceleration- and displacement-domain sensing.

Another potential advantage of ACC-based arterial pulse measurement is its ability to capture arterial wall motion along multiple directions. A three-axis ACC can simultaneously measure acceleration associated with radial and axial arterial wall motion at the CA. Previous ACC-based arterial pulse studies have focused primarily on radial arterial wall motion [6,7,18,19]. In contrast, ultrasound-based imaging studies have demonstrated that axial wall motion at the CA contains physiologically and clinically relevant information for CV assessment [37,38,39,40]. Ultrasound-based techniques provide an imaging-based approach to characterizing arterial axial wall motion, whereas ACCs offer a compact, commercially available approach to measuring arterial wall motion along multiple directions. More broadly, biomedical sensing systems involve trade-offs among sensor characteristics, electronic interfaces, signal-processing strategies, system complexity, and measurement performance, as discussed in a recent review of air-coupled ultrasound systems [41]. These considerations provide broader context for investigating three-axis ACCs as a practical approach to measuring multidirectional arterial wall motion at the CA and exploring its potential physiological relevance.

In Part I of this work, an SDOF-model-based theoretical analysis identified fundamental differences between acceleration- and displacement-domain measurements. All factors involved during pulse measurement are collectively characterized by the system parameters of the tissue-contact-sensor (TCS) stack, while MA is represented as baseline drift (BD), with its effect on the TCS stack quantified as time-varying system parameters (TVSP). Accordingly, MA affects the measured acceleration signal through two mechanisms: MA itself and TVSP-induced distortion, with the latter affecting the instantaneous parameters of individual arterial pulse harmonics. The associated time-frequency analysis (TFA) identifies the distinct effects of TVSP-induced distortion on the instantaneous parameters of individual arterial pulse harmonics, providing a mechanistic rationale for removing MA and sensor noise from a measured acceleration signal. Meanwhile, acceleration-domain sensing introduces additional amplitude and phase effects associated with the relationship between acceleration and displacement. These theoretical findings provide a theoretical basis for experimentally examining how MA and sensing modality affect the physiological information obtained from ACC-based arterial pulse measurement.

This paper, as Part II of this work, experimentally investigates these theoretical findings presented in Part I using ACC-based arterial pulse measurements at the CA at rest. Two different ACCs, a digital ACC (LIS2DW12) and an analog ACC (LIS344ALH), are first employed to measure arterial pulse signals at the CA to examine differences in their measured APW_a_ and reconstructed APW. The analog ACC and a tactile sensor are then used to directly compare acceleration- and displacement-domain pulse measurements. The measured signals are treated as nonstationary and analyzed using the SDOF time-frequency (SDOF-TF) method to obtain the instantaneous parameters of individual arterial pulse harmonics, reconstruct the APW, and derive arterial indices. The MA characteristics of ACC-based and tactile-based pulse measurements are compared, followed by comparison of the instantaneous parameters, reconstructed APW, and arterial indices extracted from the respective signals. The measured axial wall motion is also examined to investigate the feasibility of using a three-axis ACC to capture multidirectional arterial motion. The experimental observations are further evaluated against the theoretical predictions presented in Part I to determine their consistency and clarify the effects of MA and TCS system characteristics, as well as the fundamental differences between acceleration- and displacement-domain sensing. The findings provide an experimental basis for further evaluating ACC-based arterial wall motion measurements, including future quantitative comparisons with ultrasound-based measurements.

## 2. Materials and Methods

### 2.1. Measurement Hardware: Digital and Analog ACCs and a Tactile Sensor

Figure 1 illustrates the two accelerometers (ACCs) used in this study: a 3-axis digital ACC (LIS2DW12) and a 3-axis analog ACC (LIS344ALH) (STMicroelectronics, Geneva, Switzerland). These two ACCs are selected because of their high sensitivity and low noise [42,43]. The digital ACC provides a relatively simple implementation, whereas the analog ACC requires custom electrical circuits but can achieve lower sensor noise. The digital ACC is mounted on a custom PCB (1.1 cm × 1 cm × 0.2 mm) and connected to a microcontroller (Arduino Nano 33 BLE, Arduino S.r.l., Monza, Italy), which is further connected to a laptop. An Arduino program is used to communicate with and control the microcontroller, which interfaces with a LabVIEW program for real-time monitoring and recording of the three-axis signals at a sampling rate of 100 Hz. The analog ACC is also mounted on a custom PCB (1.1 cm × 1 cm × 0.2 mm), with each of its three axes connected to an identical PCB-based circuit with a cutoff frequency of 48.2 Hz. The three circuits are connected to a data acquisition (DAQ) card, and a LabVIEW 2021 (64-bit) program is used to monitor and record the three-axis signals in real time at a sampling rate of 1000 Hz. To measure arterial wall motion along three directions, each ACC is aligned with the CA and secured using medical tape, as shown in Figure 1c.

The digital and analog ACCs were operated with different electronic interfaces and data-acquisition configurations. Characterization of the complete measurement chain is important when evaluating the performance of integrated biomedical systems, as illustrated by Menniti et al. [44]. Details of the two ACC measurement systems, including their recorded zero-input noise signals and corresponding FFT spectra, are provided in the Supplementary Materials (Table S1 and Figures S1 and S2). Throughout the remainder of this paper, “digital ACC” and “analog ACC” refer to the respective complete measurement systems, including the ACCs and their associated electronic interfaces and data-acquisition components, rather than to the sensors alone.

Figure 2 illustrates the alignment of the ACC coordinate axes with the three arterial wall motion directions of the CA. Ideally, when the ACC is aligned with the center of the CA, the *z*- and *x*-axes of the ACC correspond to the radial and axial wall displacements, *u_r_*(*t*) and *u_x_*(*t*), respectively. Under this ideal alignment, the lateral wall displacement along the *y*-axis, *y*(*t*), is zero. Any off-center misalignment of the ACC with respect to the CA center results in a nonzero measured lateral displacement *y*(*t*). Nevertheless, *z*(*t*) is treated as the radial wall displacement in this study.

For comparison, a microfabricated tactile sensor is also used to measure the pulse signal at the CA. Figure 3 shows the tactile sensor used in this study and its associated PCB-based circuits for routing the sensor signals to a LabVIEW program for real-time monitoring and recording at a sampling rate of 1000 Hz. Note that the tactile sensor is held in place at the CA by the fingers during pulse measurement, allowing adjustment of the sensor location and contact pressure to acquire a measured pulse signal with optimal quality. This sensor has been extensively used for arterial pulse measurements, and details of the sensor and its application to arterial pulse measurement can be found in the literature [4,45,46]. Compared with tactile-based measurement, securing the ACC with medical tape results in lower contact pressure and reduced conformity between the ACC and the CA, which may affect the quality of the measured pulse signal. The system parameters, *m*_0_, *k*_0_, and *d*_0_, of the tissue-contact-sensor (TCS) stack, as detailed in Part I, are expected to differ between the two ACCs and the tactile sensor.

### 2.2. Measurement Protocol

This study was conducted in accordance with the requirements of the Old Dominion University (ODU) Institutional Review Board (IRB). Measurements involving ten subjects using the two ACCs were conducted under an ODU IRB-approved protocol (IRB Approval No. 26-021), and written informed consent was obtained from all subjects. Measurements involving three additional subjects using the analog ACC and tactile sensor were acknowledged by the ODU IRB.

Under the approved protocol, the first five subjects underwent both digital and analog ACC measurements, whereas the subsequent five underwent analog ACC measurements only. All ten subjects met the inclusion criteria: male, aged 21–40 years, and without diagnosed diseases. Of the first five subjects, recordings from three met the signal-quality criterion of lasting longer than 30 s without excessive sensor noise and substantial MA-related fluctuations and were included in the analysis. Of the subsequent five subjects, recordings from two met the same criterion and were included. Thus, ACC recordings from five of the ten subjects recruited under the approved protocol were analyzed. The three additional subjects who underwent sequential measurements using the analog ACC and tactile sensor also met the inclusion criteria; these measurements were conducted under the ODU IRB acknowledgment.

In addition to experimentally investigating the technical issues associated with ACC-based arterial pulse measurement identified through the theoretical analysis in Part I, this study aimed to compare ACC-based and tactile-sensor-based pulse measurements to identify practical technical challenges associated with ACC-based measurements. Accordingly, the study included a relatively small number of subjects and was not designed for statistical analysis.

For measurements using the two ACCs, each subject sat in a chair and was instructed to remain still. Blood pressure (BP) and heart rate (HR) were first measured using a medical instrument (Mindray Passport 12 (Mindray, Shenzhen, China)). The digital ACC was secured with double-sided tape and placed over the left CA to record arterial wall motion for approximately 1 min. The digital ACC was then replaced with the analog ACC, and the measurement procedure was repeated. Five subjects underwent measurements using both ACCs. Preliminary examination of the recorded data indicated that signals obtained using the digital ACC were generally noisier than those obtained using the analog ACC. However, some measurements obtained using the analog ACC exhibited substantial fluctuations associated with MA. Therefore, an additional five subjects underwent measurements using the analog ACC only to investigate whether substantial MA fluctuations were also observed in other analog-ACC measurements.

To further compare ACC-based and tactile-based measurements, another three subjects, sitting in a chair and remaining still, were subsequently measured using the analog ACC and tactile sensor sequentially. To improve conformity between the tactile sensor and the artery, the same PDMS layer (~1 mm) was placed between the tactile sensor and the skin during tactile-sensor measurements [4].

The subject characteristics are summarized in Table 1. As noted earlier, securing the ACC over the CA with medical tape may not provide sufficiently firm contact, potentially resulting in relatively large MA-related fluctuations even with slight subject motion, excluding respiration. Insufficiently firm contact may also reduce the measured pulse amplitude, thereby increasing the relative contribution of sensor noise to the original recording. Among the five subjects measured using both ACCs, only three (Subjects #1–#3) provided pulse recordings lasting longer than 30 s with reasonable sensor noise and MA fluctuations relatively small compared with the pulse-induced component in the original recording. Among the five subjects measured using only the analog ACC, only two (Subjects #4 and #5) met these signal-quality criterion. Examples of pulse recordings that met and did not meet these signal-quality criterion are provided in the Supplementary Materials (Figures S3 and S4). The three additional subjects (Subjects #6–#8) underwent sequential measurements using the analog ACC and tactile sensor. Their BP and HR were not recorded.

### 2.3. Time-Frequency Analysis of Measured Acceleration Signals Using an SDOF-TF Method

Based on the theoretical analysis in Part I, the distinct effects of MA on the three instantaneous parameters—instantaneous amplitude (IA), instantaneous frequency (IF), and instantaneous initial phase (IIP)—of a measured acceleration signal are identical to those on their counterparts in a measured displacement signal obtained using a tactile sensor. As such, the single-degree-of-freedom time-frequency (SDOF-TF) method developed for time-frequency analysis (TFA) of a measured displacement signal and subsequent removal of MA and sensor noise can be directly adopted to analyze a measured acceleration signal.

Figure 4 illustrates the signal-processing algorithm for time-frequency analysis (TFA) of a measured acceleration signal based on the SDOF-TF method. The measured pulse signal was first resampled to 125 Hz and then low-pass filtered to remove frequency components above 15 Hz. The resulting signal, representing the measured acceleration along the radial direction of arterial wall motion, is denoted by *a_z_*(*t*) and can be expressed as follows:

*a_z_*(*t*) = *a*_0_(*t*) + *a_bd_*(*t*) with *a*_0_(*t*) = *a_tf_*(*t*) + *a_tvsp_*(*t*) + *a_noise_*(*t*) and *a_tf_*(*t*) = *a_C_*(*t*) (1)

where *a_noise_*(*t*), *a_bd_*(*t*), and *a_tvsp_*(*t*) denote sensor noise, the BD (<0.7 Hz), and TVSP-induced distortion, respectively; *a_tf_*(*t*), with time-varying frequency, corresponds to the detected acceleration signal free of MA and is identical to *a_C_*(*t*) in Part I. First, Fast Fourier transform (FFT) is conducted on *a_z_*(*t*) to obtain the frequency *f_a_*_0_ of the 1st harmonic of the measured signal. Subsequently, a low-pass filter (LPF) is used to remove the BD *a_bd_*(*t*) from *a_z_*(*t*). A bandpass filter (BPF) is then successively applied to *a*_0_(*t*) to decompose it into individual harmonics, with the *i*th harmonic denoted by *a_sd_i_*(*t*).

Hilbert vibration decomposition (HVD) [47] is then used to extract IA, *A_i_*(*t*), IF, *f_i_*(*t*), and IIP, *ϕ*_0*i*_(*t*), of each harmonic. Accordingly, the *i*th harmonic can be expressed as:

(2) $$a_{i}\left(t\right)=A_{i}\left(t\right)\cdot \cos\;(2\pi \cdot \int f_{\mathrm{ai}}\left(t\right)\mathrm{dt}+\phi_{0i}\left(t\right))=a_{\mathrm{tf}\_i}\left(t\right)+a_{\mathrm{tvsp}\_i}\left(t\right)$$

This expression is consistent with the theoretical finding in Part I that TVSP-induced distortion is embedded in each individual harmonic. Accordingly, the sensor noise can be calculated as:

*a_noise_*(*t*) = *a*_0_(*t*) − *a_HVD_*(*t*) (3)

where

(4) $$a_{\mathrm{HVD}}\left(t\right)=\sum_{i=1}^{N}a_{i}\left(t\right)=a_{\mathrm{tf}}\left(t\right)+a_{\mathrm{tvsp}}\left(t\right)$$

To remove TVSP-induced distortion (i.e., *a_tvsp_*(*t*)) from *a_HVD_*(*t*), the mean of *A_i_*(*t*), denoted by $\bar{A_{i}}\left(t\right)$ and the mean of *ϕ*_0*i*_(*t*), denoted by ${\bar{\phi }}_{0i}$, are obtained. Then, free of MA and sensor noise, the pulse signal *a_tf_*(*t*) with time-varying frequency or heart rate variability (HRV) and *a_cf_*(*t*) with constant frequency are reconstructed [48]:

(5) $$a_{\mathrm{tf}}\left(t\right)=\sum_{i=1}^{N}a_{\mathrm{tf}\_i}\left(t\right)\;\mathrm{with}\;a_{\mathrm{tf}\_i}\left(t\right)=\bar{A_{i}}\cdot \cos\;(2\pi \cdot \int f_{\mathrm{ai}}\left(t\right)\mathrm{dt}+{\bar{\phi }}_{0i})$$

(6) $$a_{\mathrm{cf}}\left(t\right)=\sum_{i=1}^{N}a_{\mathrm{cf}\_i}\left(t\right)\;\mathrm{with}\;a_{\mathrm{cf}\_i}\left(t\right)=\bar{A_{i}}\cdot \cos\;(2\pi \cdot if_{a0}t+{\bar{\phi }}_{0i})$$

where *a_cf_*(*t*) can be treated as the reconstructed *APW_a_* free of beat-to-beat frequency variations.

### 2.4. Extraction of Arterial Indices from a_tf_(t) and Reconstruction of APW

#### 2.4.1. Extraction of Arterial Indices from *a_tf_*(*t*)

Figure 5 illustrates the characteristic features of *a_tf_*(*t*) used to extract arterial indices. Beat-based heart rate (HR) and HRV were determined using both peak *a* and the valley *b* of each pulse cycle. The labels *a* and *b* are adopted because these features are commonly used in the analysis of the second-derivative waveform of PPG signals [49,50,51,52]. The inter-beat intervals were calculated independently from the temporal differences Δ*t_k_* between consecutive *a* peaks and consecutive *b* valleys:

(7) $$\Delta t_{k}^{a-a}=t\left(a_{k+1}\right)-t\left(a_{k}\right)\;\mathrm{and}\;\Delta t_{k}^{b-b}=t\left(b_{k+1}\right)-t\left(b_{k}\right)$$

For comparison with IF-based HR and HRV, the time interval between successive pulse peaks (or valleys) is converted to HR by taking its reciprocal and multiplying by 60, thereby expressing HR in beats per minute (bpm). Accordingly, the beat-based HR, HR*_k_*, in bmp for each pulse cycle was calculated as:

(8) $${\mathrm{HR}}_{\mathrm{ka}}=\frac{60}{\Delta t_{k}^{a-a}}\;\mathrm{and}\;{\mathrm{HR}}_{\mathrm{kb}}=\frac{60}{\Delta t_{k}^{b-b}}$$

where HR*_ka_* and HR*_kb_* are from the peak and the valley values in successive pulse cycles, respectively.

The standard deviation of normal-to-normal intervals (SDNN), which quantifies overall beat-based HRV over the analyzed recording period, was calculated as:

(9) $$\mathrm{SDNN}=\sqrt{\frac{1}{N-1}\sum_{k=1}^{N}{\left({\mathrm{HR}}_{k}-\bar{{\mathrm{HR}}_{k}}\right)}^{2}}$$

where *N* is the number of analyzed pulse cycles and $\bar{{\mathrm{HR}}_{k}}$ is the mean value from analyzed pulse cycles.

In addition to beat-based HR and HRV, two feature-based arterial indices were extracted from each pulse cycle: the *b*/*a* ratio and the temporal interval between the *a* peak and *b* valley, Δ*t_a_*_−*b*_ = *t_b_* − *t_a_*. The mean values of *b*/*a* and Δ*t_a_*_−*b*_ across the analyzed pulse cycles were used as arterial indices.

Based on the TFA of the measured acceleration signal, IF-based HR and IF-based HRV can also be calculated [48,53]. In addition, respiration rate (RR) and respiration modulation (RM) can be determined from both IF and IIP [48,53]. Detailed derivations of these arterial indices have been reported in the literature [48,53]. The beat-based and IF-based HR and HRV are compared in Section 3.

#### 2.4.2. Reconstruction of APW for Normalized Harmonic Amplitude Distribution

Here, the corresponding APW is reconstructed from the extracted *a_tf_*(*t*) using two methods. First, based on the relationship between acceleration and displacement, the APW can be approximated by dividing each harmonic component of *a_tf_*(*t*) by the square of its time-varying angular frequency:

(10) $$z_{\mathrm{tf}}\left(t\right)\approx \sum_{i=1}^{N}-\frac{\bar{A_{i}}}{4\pi^{2}f_{\mathrm{ai}}^{2}\left(t\right)}\cdot \cos\;(2\pi \cdot \int f_{\mathrm{ai}}\left(t\right)\mathrm{dt}+\bar{\phi_{0i}})$$

This acceleration-to-displacement conversion is exact for a sinusoidal signal with constant frequency. However, because the IF of the arterial pulse signal varies over time, the relationship between acceleration and displacement cannot be accurately represented by Equation (10). To obtain the constant-frequency reconstruction, *z_cf_*(*t*), $f_{a_{i}}(t)$ in Equation (10) is replaced by *if_a_*_0_:

(11) $$z_{\mathrm{cf}}\left(t\right)\approx \sum_{i=1}^{N}A_{\mathrm{icf}}\cdot \cos\;(2\pi \cdot if_{a0}t+\bar{\phi_{0i}})\;\mathrm{with}\;A_{\mathrm{icf}}=\mathrm{mean}(-\frac{\bar{A_{i}}}{4\pi^{2}f_{\mathrm{ai}}^{2}\left(t\right)})$$

where *A_icf_* denotes the amplitude of the *i*th harmonic in *z_cf_*(*t*).

In theory, the APW can be more accurately reconstructed by directly integrating the acceleration signal twice. Accordingly, the second method reconstructs the APW by double integration of the extracted *a*_tf_(*t*), with a low-pass filter (LPF) with a cutoff frequency of 0.5 Hz applied during each integration process. The following derivation illustrates the technical challenges associated with double integration. For clarity, the symbols used to represent the parameters in this derivation differ from those defined in Section 2.3.

The extracted *a_tf_*(*t*) from a measured acceleration signal is represented as *a*(*t*)*e^jω^*^(*t*)*t*^. First, the built-in MATLAB R2023b function cumtrapz is used to numerically integrate the signal and obtain the velocity:

(12) $$v\left(t\right)=\int a\left(t\right)e^{\mathrm{j\omega}(t)t}\mathrm{dt}=\frac{a(t)}{\mathrm{j\omega}(t)}e^{\mathrm{j\omega}(t)t}-\int \frac{d}{\mathrm{dt}}\left(\frac{a(t)}{\mathrm{j\omega}(t)}\right)e^{\mathrm{j\omega}(t)t}\mathrm{dt}$$

where

(13) $$\int \frac{d}{\mathrm{dt}}\left(\frac{a\left(t\right)}{\mathrm{j\omega}\left(t\right)}\right)e^{\mathrm{j\omega}\left(t\right)t}\mathrm{dt}=\int \frac{\dot{a}(t)\omega (t)-a(t)\dot{\omega }(t)}{j\omega^{2}(t)}e^{\mathrm{j\omega}(t)t}\mathrm{dt}$$

The second term in Equation (12) represents the additional distortion introduced by the time-varying amplitude and time-varying frequency, which prevent the integration from being a simple inverse operation of differentiation. This term becomes increasingly prominent at low frequencies, because of the 1/*ω*^2^(*t*) dependence in Equation (13), making the integration particularly sensitive to slowly varying components, *a*(*t*) and *ω*(*t*), associated with sensor noise and numerical integration errors. Therefore, after the first integration, a LPF with a cutoff frequency of 0.5 Hz is applied to suppress these low-frequency components before the second integration.

The second integration is then performed on the filtered velocity signal to obtain the APW, denoted by *z*(*t*):

(14) $$z(t)=\int \int a(t)e^{\mathrm{j\omega}(t)t}\mathrm{dt}\;\mathrm{dt}=-\frac{a(t)}{{\left(\omega (t)\right)}^{2}}e^{\mathrm{j\omega}(t)t}-\int \int \frac{\dot{a}(t)\omega (t)-a(t)\dot{\omega }(t)}{j\omega^{2}(t)}e^{\mathrm{j\omega}(t)t}\mathrm{dt}\;\mathrm{dt}$$

After the second integration, the same LPF is applied to further suppress residual low-frequency components resulting from sensor noise and numerical integration error. The resulting signal is then used as the reconstructed APW.

Equations (12)–(14) demonstrate that ACC-to-displacement reconstruction cannot be treated as an exact inverse transformation because *a*(*t*) and *ω*(*t*) introduce additional terms that are not recovered by simple integration. However, as shown in Section 3, the difference in the reconstructed APW between the two methods is very small, indicating that when *a*(*t*) and *ω*(*t*) vary slowly with time, Equations (10) and (11) provide good approximations for reconstructing the APW from *a_tf_*(*t*). After APW reconstruction, the harmonic amplitude distributions normalized to the first-harmonic amplitude, *A_i_*_cf_/*A*_1cf_, serve as APW-based arterial indices.

### 2.5. Derivation of Displacement-Domain BD from Acceleration-Domain BD

To reconstruct the displacement-domain BD, *z*_bd_(*t*), from the measured acceleration-domain BD, *a*_bd_(*t*), the built-in MATLAB functions, cumtrapz and detrend, were applied sequentially at each integration step. As indicated by Equation (13), double integration has a frequency response proportional to 1/*ω*^2^. Therefore, at frequencies below 0.7 Hz, even small noise and MA-related errors contained in *a*_bd_(*t*) can be strongly amplified during double integration, potentially resulting in unrealistically large low-frequency components in the reconstructed *z*_bd_(*t*).

As shown in Section 3, although the magnitude of the obtained *z_bd_*(*t*) through double integration is approximately two orders of magnitude larger than the physiologically realistic displacement range of approximately 1–3 mm, differentiating *z_bd_*(*t*) twice produces an acceleration signal, *a_bd_*__*re*_(*t*), that closely reproduces the original *a_bd_*(*t*), with only a small constant offset. Thus, the temporal variation of *a_bd_*__*re*_(*t*) is essentially identical to that of the original *a_bd_*(*t*). This behavior can be attributed to the predominantly low-frequency nature of BD. Because BD is concentrated within a relatively narrow low-frequency range, double integration strongly increases its magnitude through the frequency-dependent 1/*ω*^2^ scaling while having a comparatively limited effect on its overall temporal pattern.

Accordingly, although the absolute magnitude of the reconstructed *z_bd_*(*t*) is not physiologically reliable, its temporal variation can still be used to characterize the displacement-domain BD. Therefore, *z_bd_*(*t*)/100 is used as a scaled representation of the displacement-domain BD derived from the acceleration measurement and compared with its counterpart obtained from the tactile-based measurement. This approach enables the reconstructed displacement-domain BD from the ACC-based measurement to be incorporated into the numerical simulation in Part I and compared with the corresponding BD obtained from the tactile-based measurement.

It is worth noting that this behavior differs from that observed when the same double-integration and detrending procedure is applied to the acceleration signal *a*_0_(*t*), which contains multiple harmonics, sensor noise, and TVSP-induced distortion. Because double integration applies frequency-dependent scaling to different harmonic components, sensor noise and TVSP-induced distortion associated with these components can be affected differently. Consequently, subsequent double differentiation of the reconstructed signal, *a*_0_*re*_(*t*), may produce noticeable temporal differences from the original acceleration, *a*_0_(*t*), despite the theoretical reversibility of integration and differentiation for an ideal signal. As shown later, this difference highlights the greater sensitivity of pulse-acceleration reconstruction to its multiple harmonics, TVSP-induced distortion, and sensor noise than that of low-frequency BD reconstruction. It also underscores the importance of applying TFA to measured acceleration signals based on the SDOF-TF method to characterize the pulse signal and extract its instantaneous parameters, thereby obtaining *a_tf_*(*t*) free of sensor noise and TVSP-induced distortion.

## 3. Results

Given the absence of statistical analyses and quantitative comparisons of some parameter values, the observations from ACC-based and tactile-based measurements and the comparisons between them are primarily descriptive. Accordingly, terms such as “higher-quality,” “substantial difference,” “consistent,” and “more reliable” describe qualitative observations in the measurements analyzed rather than statistically established differences. Specifically, higher-quality refers to pulse recordings in which TVSP-induced distortion and sensor noise are relatively small compared with the pulse-induced component of the measured signal after BD removal. Substantial difference refers to a noticeable difference in observed parameter values or signal characteristics, whereas consistent refers to a recurring pattern in the measurements examined. More reliable refers to a measurement or an extracted parameter expected to contain less error based on the observed signal characteristics, rather than reliability established through quantitative evaluation. These terms should therefore be interpreted descriptively and within the scope of the measurements analyzed.

### 3.1. Comparison of Radial Pulse Signals Between Digital and Analog ACC Measurements

Figure 6 shows the measured radial pulse signal (corresponding to *u_r_*(*t*)) of Subject #2 using the digital ACC and its analyzed results. The MA, quantified as *a*_bd_(*t*), varies with time at a much slower rate than *a*_0_(*t*). Except for two large fluctuations at approximately 6 and 27 s, the amplitude of the MA is smaller than that of *a*_0_(*t*). Accordingly, except during these two large fluctuations, when the pulse information in *a*_0_(*t*) is markedly distorted, the pulse information remains clearly visible. The frequency spectra of the derived signals reveal that *a*_tvsp_(*t*) and *a*_noise_(*t*) are comparable in amplitude to *a*_tf_(*t*) at some harmonics. While the IA, *A_i_*(*t*), is greatly affected by MA, the IF, $f_{a_{i}}(t)$, and the IIP, *ϕ*_0*i*_(*t*), show no significant temporal variation. As shown in Figure 6f, both *a*_tvsp_(*t*) and *a*_noise_(*t*) are comparable in amplitude to *a*_tf_(*t*) over most of each pulse cycle, except near the peaks and valleys, where *a*_tf_(*t*) has substantially larger amplitude. This is because the acceleration-domain arterial pulse signal fluctuates around zero over most of the pulse cycle, making the relative effects of MA and sensor noise substantially more pronounced. Consequently, the physiological information contained in most of these data points is unreliable. Moreover, the secondary peak within each pulse cycle is also substantially affected by *a*_tvsp_(*t*) and *a*_noise_(*t*). Figure 6g shows no large difference between *a*_tf_(*t*) and *a*_cf_(*t*), indicating low HRV. As shown in Figure 6h, the reconstructed *z*_cf_(*t*) deviates substantially from that of a healthy young male subject measured using a tactile sensor [48].

Figure 7 shows the measured pulse signal of Subject #2 using the analog ACC and its analyzed results. The MA, *a*_bd_(*t*), is much smaller than the pulse component in *a*_0_(*t*), and no extreme distortion of the pulse information is observed. The frequency spectra of the derived signals show that *a*_tvsp_(*t*) and *a*_noise_(*t*) are small at the lower harmonics (*n* ≤ 3) but become comparable in amplitude to *a*_tf_(*t*) at higher harmonics. As identified in Part I, MA greatly affects the IA, *A_i_*(*t*), but only slightly affects the IF, $f_{a_{i}}(t)$, and has almost no effect on the IIP, *ϕ*_0*i*_(*t*). As shown in Figure 7f, *a*_tvsp_(*t*) is much smaller than *a*_tf_(*t*) at the peaks, valleys, and secondary peaks. Meanwhile, *a*_noise_(*t*) is much lower than *a*_tvsp_(*t*). However, between the secondary peak and the peak, both *a*_tvsp_(*t*) and *a*_noise_(*t*) are comparable in amplitude to *a*_tf_(*t*). This is because the arterial pulse signal itself fluctuates around zero in this segment, making the relative effects of *a*_tvsp_(*t*) and *a*_noise_(*t*) more pronounced and the physiological information in this segment less reliable. Figure 7g shows no large difference between *a*_tf_(*t*) and *a*_cf_(*t*), indicating low HRV. As shown in Figure 7h, the reconstructed *z*_cf_(*t*) deviates substantially from that of a healthy young male subject measured using a tactile sensor [44]. Notably, the amplitude of *z*_cf_(*t*) is comparable between the digital and analog ACC measurements.

A comparison of the analyzed pulse signals from the same subject measured using the two ACCs reveals that the digital ACC exhibits substantially higher sensor noise and TVSP-induced distortion, even during segments without large MA fluctuations. In contrast, the analog ACC provides a higher-quality signal. Based on the above analysis, *a*_tf_(*t*)/(*a*_tvsp_(*t*) + *a*_noise_(*t*)) can be used as an indicator of measured signal quality. As shown in Table S2 in the Supplementary Materials, the values of *a*_tf_(*t*)/(*a*_tvsp_(*t*) + *a*_noise_(*t*)) for the first three subjects in Table 1 further demonstrate the overall better performance of the analog ACC. Therefore, the remaining analyses focus exclusively on measurements obtained using the analog ACC. Table S3 in the Supplementary Materials summarizes the values of *a*_tf_(*t*)/(*a*_tvsp_(*t*) + *a*_noise_(*t*)) for the eight subjects whose measured pulse signals were analyzed.

### 3.2. SDOF-TF Reconstruction of z_tf_(t) (i.e., APW) and Time-Domain Analysis of a_bd_(t) and a_0_(t) for Radial Pulse Signals Measured Using the Analog ACC

Figure 8 compares the reconstructed APW, *z_tf_*(*t*), for three subjects using the two methods described in Section 2.4.2. Here, *z_tf_*(*t*) represents the one obtained using Equation (10); *z_re_*(*t*) represents the one obtained using double integration and the LPF; and Δ*z* = *z_tf_*(*t*) − *z_re_*(*t*). Evidently, the difference between the two methods is very small, indicating the slow time-varying nature of *a*(*t*) and *ω*(*t*) in Equation (12).

Figure 9 compares *a_bd_*(*t*) extracted from *a_z_*(*t*) with the reconstructed *a_bd_*__*re*_(*t*), obtained by taking the second-order time derivative of the reconstructed *z_bd_*(*t*), for Subject #8. Note that *z_bd_*(*t*) is obtained solely by double integration and detrending of *a_bd_*(*t*) in the time domain. The difference between the two signals, Δ*a_bd_*(*t*) = *a_bd_*(*t*) − *a_bd_*__*re*_(*t*), is a small constant offset, indicating the effectiveness of the method for obtaining displacement-domain MA. In contrast, when the same method is applied to *a*_0_(*t*) extracted from *a_z_*(*t*), the reconstructed *a*_0_*re*_(*t*) differs from the original *a*_0_(*t*) in a time-varying manner, with Δ*a*_0_(*t*) = *a*_0_(*t*) − *a*_0_*re*_(*t*) varying with time. This result demonstrates the need to remove TVSP-induced distortion and sensor noise before reconstructing *APW_a_* to improve its accuracy. Figures S9 and S14 in the Supplementary Materials show the corresponding results for Subjects #6 and #7, respectively, leading to the same observations.

### 3.3. Comparison of Radial, Axial, and Lateral Pulse Signals from the Analog ACC Measurements

Figure 10 compares the three reconstructed APWs for the three arterial wall motion directions from the measured pulse signals of two subjects, where *z*(*t*) = *z_tf_*(*t*), *y*(*t*) = *y_cf_*(*t*), and *x*(*t*) = *x_cf_*(*t*). As shown in Figure 10a, the lateral displacement, *y*(*t*), is only moderately smaller than the radial wall displacement, *z*(*t*), suggesting appreciable off-center positioning of the ACC relative to the CA. Because the ACC was placed against the skin and secured with tape, substantial directional misalignment due to tilting of the ACC relative to the skin surface was considered less likely. Therefore, off-center positioning is considered a potential primary contributor to the lateral displacement in the present measurement setup, although its contribution cannot be quantitatively established from the available data. Other potential contributors include residual directional misalignment between the ACC measurement axes and the artery’s radial, axial, and lateral directions, intrinsic cross-axis sensitivity, neck movement, and sensor rotation during measurement. Meanwhile, *y*(*t*) follows *z*(*t*) in time, with a constant phase delay, suggesting a temporal relationship between the lateral and radial signals. In contrast, the axial wall displacement, *x*(*t*), differs substantially from *z*(*t*) in terms of pulse waveform, and *x*(*t*) is also much smaller than *z*(*t*) in amplitude.

As shown in Figure 10b, *z*(*t*) is much larger than *y*(*t*), indicating a much smaller off-center misalignment between the ACC and the CA. Furthermore, there is also a constant phase delay of *y*(*t*) relative to *z*(*t*). However, note that the pulse waveform of *x*(*t*) in Figure 10b is substantially different from that in Figure 10a. In both subjects, the amplitude of *x*(*t*) is much smaller than that of *z*(*t*). The corresponding measured acceleration signals for the two subjects are included in Figures S5 and S6 in the Supplementary Materials. Moreover, Figure S8 in the Supplementary Materials shows the reconstructed APWs for the three wall motion directions for the other three subjects (#1, #3, and #5). The observed findings on *x*(*t*), *y*(*t*), and *z*(*t*) in those three subjects are consistent with those observed in Figure 10. In particular, the pulse waveform of *x*(*t*) varies greatly among all five subjects.

### 3.4. Extraction of Arterial Indices from Radial Pulse Signals Measured Using the Analog ACC Measurments

Table 2a summarizes the beat-based arterial indices extracted from the measured acceleration signals using the analog ACC. The *b*/*a* ratio ranges from −0.47 to −1.56, while Δ*t_a_*_−*b*_ ranges from 0.0514 to 0.0952 s. The *b*/*a* ratio has been widely used in second-derivative photoplethysmography (SDPTG) to characterize arterial waveform morphology and vascular properties, and has been reported to vary with vascular aging, arterial distensibility, blood pressure, and other CV risk factors [52,54,55,56,57,58,59]. The *b*/*a* ratio has also been extracted from the second-derivative of carotid diameter waveforms for characterizing arterial stiffness and vascular aging [60]. The range of *b*/*a* values obtained in the present study is of a similar order of magnitude to values reported in the SDPTG literature [52,54,55,58,59]. However, direct comparison should be made with caution because the present *b*/*a* ratios are obtained from carotid arterial-wall acceleration in ACC-based measurement, whereas conventional *b*/*a* ratios are primarily obtained from the second derivative of fingertip PPG.

The Δ*t_a_*_−*b*_ ranges from 0.0514 to 0.0952 s (51–95 ms), representing the temporal separation between the *a*- and *b*-waves. Such a time interval is physiologically plausible for the successive features of the systolic portion of the APW, although a standardized reference range for Δ*t_a_*_−*b*_ specifically from carotid arterial-wall acceleration has not been established. The HR values obtained from the *b*-*b* and *a*-*a* intervals are highly consistent within each subject, with differences of less than 0.2 bpm for all subjects. In contrast, the HRV values obtained from the *b*-*b* and *a*-*a* intervals show noticeable differences for Subjects #1, #2, and #3, with differences of 0.0153, 0.0100, and 0.0033 s, respectively, while the HRV values are relatively consistent for the other subjects. Note that the HR measured using the medical instrument is nearly identical to the derived HR for Subjects #2, #3, and #5. However, for Subjects #1 and #4, there is a noticeable difference in HR between the medical instrument and the analog ACC measurements, with values of 64 vs. 69 bpm and 107 vs. 102 bpm, respectively.

Table 2b further compares the HR and HRV obtained using the IF-based method with those obtained from the *a*-*a* and *b*-*b* intervals in Table 2a. For each subject, the three HR values obtained using the *b*-*b*, *a*-*a*, and IF-based methods are highly consistent, with differences of less than 0.3 bpm. In contrast, the three HRV values show greater subject-dependent differences. For Subject #1, the IF-based HRV (5.0574 bpm) is lower than those obtained from the *b*-*b* (5.3318 bpm) and *a*-*a* (6.2024 bpm) intervals. Similarly, for Subject #2, the IF-based HRV (4.2429 bpm) is lower than the *b*-*b* (6.0580 bpm) and *a*-*a* (5.0476 bpm) values. In contrast, for Subject #3, the IF-based HRV (2.6333 bpm) is substantially higher than the *b*-*b* (0.8483 bpm) and *a*-*a* (0.7915 bpm) values. For the remaining subjects, the three HRV values are relatively consistent. Overall, the close agreement among the three HR estimates supports the consistency of HR determination using the different methods, whereas the greater differences in HRV for some subjects indicate that HRV is more sensitive to the method used for its determination. It is worth noting that different levels of MA-induced error are reflected in the three methods [53].

As shown in Table 2b, the IF-based RR is lower than the IIP-based RR for each subject. It should be noted that IIP was derived from IF during signal processing, as described in Part I. Compared with the IIP-based estimates, several IF-based RR values fall below the commonly cited normal at-rest RR range of 12–20 breaths/min for adults [61]. In contrast, all IIP-based RR values fall within or very close to this range. This observation suggests that the IIP-based RR estimates are more consistent with the cited reference range. However, because no reference respiratory signal was recorded, the accuracy of either estimate could not be independently established. Respiratory measurements derived from indirect sensing methods may also be affected by the measurement principle, signal quality, and artifacts [62,63,64].

Regarding RM, the IIP-based RM values are consistent with related findings reported in the literature [65]. Together with the greater consistency of the IIP-based RR estimates with the cited reference range, this finding provides support for considering the IIP-based RM estimates more trustworthy than the IF-based estimates. Nevertheless, this comparison does not constitute independent validation of RM as a physiological index, and further validation against appropriate reference measurements is needed.

Figure 11 compares *a_tf_*(*t*) with *a_tvsp_*(*t*) and *a_noise_*(*t*) for Subjects #1, #7, and #8. As shown in Figure 11a, *a_tvsp_*(*t*) and *a_noise_*(*t*) are comparable in amplitude, and both are also comparable in amplitude to *a_tf_*(*t*). This may explain the large difference in beat-based HRV between the *b*-*b* and *a*-*a* intervals, as well as the noticeable difference in HR between the medical instrument and the analog ACC measurements. In contrast, as shown in Figure 11b, *a_tvsp_*(*t*) is very small compared with *a_tf_*(*t*), whereas *a_noise_*(*t*) is substantially larger than *a_tvsp_*(*t*). Nevertheless, *a_tvsp_*(*t*) remains small relative to *a_tf_*(*t*) at the peaks and valleys. This may explain the slight difference in the derived HRV between the *b*-*b* and *a*-*a* intervals. As shown in Figure 11c, both *a_tvsp_*(*t*) and *a_noise_*(*t*) are very small throughout the pulse cycles, possibly contributing to the slight difference in the derived HRV between the *b*-*b* and *a*-*a* intervals.

Table 3 shows the harmonic amplitude distribution normalized to the amplitude of the 1st harmonic in the extracted APW. Although the harmonic amplitudes exhibit a decreasing trend with increasing harmonic order, the amplitude of the 2nd harmonic is below 22% of that of the 1st harmonic for the majority of the subjects, except for Subject #3, who has the highest BMI. The harmonic power distribution in Table S4 in the Supplementary Materials further shows that, except for Subject #3, more than 90% of the power in the measured pulse signal is concentrated in the 1st harmonic.

### 3.5. Comparison Between ACC-Based and Tactile-Based Pulse Measurements

We compared ACC-based (analog ACC) and tactile-based pulse measurements across Subjects #6, #7, and #8. Because the analog ACC measurements for Subject #8 exhibited the lowest MA and sensor noise, we present the detailed results for Subject #8 here. Corresponding data for Subjects #6 and #7 are provided in the Supplementary Materials.

#### 3.5.1. Measured Pulse Signal, MA, TVSP-Induced Distortion, Sensor Noise, and Signal Reconstruction

Figure 12 illustrates the radial acceleration signal measured using the analog ACC and its analyzed results. Due to the small MA, *a_bd_*(*t*), shown in Figure 12a, the frequency spectrum of *a*_0_(*t*) exhibits clear harmonic peaks. Although the effect of MA on the IA, *A_i_*(*t*), is obvious, the IF, *f_ai_*(*t*), and the IIP, *ϕ*_0*i*_(*t*), show very small variation over time.

As shown in Figure 12f, both *a_tvsp_*(*t*) and *a_noise_*(*t*) remain very small at the peaks and valleys relative to *a*_tf_(*t*), indicating that these features are highly reliable. However, *a*_tvsp_(*t*) and *a*_noise_(*t*) fluctuate near zero across the remainder of the pulse cycle, introducing non-negligible contamination that distorts the reconstructed APW. In Figure 12g, the slight difference between *a*_tf_(*t*) and *a*_cf_(*t*) reflects low HRV. Finally, the reconstructed APW, *z*_cf_(*t*), in Figure 12h differs substantially in both waveform morphology and amplitude from the tactile sensor measurement, as detailed below.

Figure 13 shows the measured pulse signal, *z*_mea_(*t*) = *z*_0_(*t*) + *z*_bd_(*t*), obtained for Subject #8 using the tactile sensor. The MA, *z*_bd_(*t*), is relatively large compared to that in the ACC measurement, though it remains small relative to the pulse information in the measured signal. The frequency spectra of the derived signals demonstrate very low amplitudes for *z*_tvsp_(*t*) and *z*_noise_(*t*) compared to *z*_tf_(*t*). While the IA, *A_i_*(*t*), is influenced by MA, the distortion is less severe than in the ACC signal. Meanwhile, the IF, *f_i_*(*t*), and IIP, *ϕ*_0*i*_(*t*), remain largely unaffected by MA. As shown in Figure 13f, *z*_tvsp_(*t*) and *z*_noise_(*t*) are significantly smaller than *z*_tf_(*t*). Crucially, except near the start and end of a pulse cycle—where *z*_tf_(*t*) experiences a DC offset due to harmonic reconstruction—the values across the cycle remain well above zero. This confirms that most data points within each pulse cycle yield reliable information for extracting the APW, *z*_tf_(*t*). Figure 13g highlights the minor difference between *z*_tf_(*t*) and *z*_cf_(*t*), further confirming a low HRV. Comparing Figure 12h and Figure 13g reveals a substantial difference in pulse waveform, with the amplitude of the ACC-based signal being approximately two orders of magnitude larger than that of the tactile-based signal.

#### 3.5.2. Normalized Harmonic Amplitude Distribution and Sensor Noise in the Frequency Domain

Figure 14 compares the normalized harmonic amplitude distributions, *A_icf_*/*A*_1*cf*_, between the ACC-based and tactile-based measurements. The normalized harmonic amplitudes for *n* ≥ 2 obtained via the tactile sensor are consistently higher than those measured using the ACC, explaining the significant difference in the reconstructed *z*_cf_(*t*) between the two measurement modalities. Similar trends were observed for Subjects #6 and #7, as shown in Figures S11 and S17 of the Supplementary Materials.

According to Part I, when BD is omitted, the pulse signal *z_ACC_*(*t*) measured by an ACC is related to the arterial pulse signal *u_r_*(*t*) in the CA by [66]:

(15) $$z_{\mathrm{ACC}}\left(t\right)\approx \frac{k_{t}+jd_{t}\omega_{u_{r}}}{-m_{t}\omega_{u_{r}}^{2}+({\dot{m}}_{t}+d_{t})j\omega_{u_{r}}+k_{t}}\cdot u_{r}\left(t\right)\;(\mathrm{ACC})$$

Meanwhile, the pulse signal *z_Tactile_*(*t*) measured by a tactile sensor is related to *u_r_*(*t*) by [67,68]:

(16) $$z_{\mathrm{Tactile}}\left(t\right)\approx \frac{k_{t}+jd_{t}\cdot \omega_{u_{r}}}{-m_{t}\cdot \omega_{u_{r}}^{2}+j\left({\dot{m}}_{t}+d_{t}\right)\cdot \omega_{u_{r}}+k_{t}}\cdot \frac{-m_{s}\omega_{u_{r}}^{2}}{k_{s}+jd_{s}\omega_{u_{r}}}\cdot u_{r}\left(t\right)\;(\mathrm{tactile sensor})$$

where *k_s_*, *d_s_*, and *m_s_* denote the three constant system parameters associated with the tactile sensor, and $\omega_{u_{r}}$ denotes the instantaneous angular frequency of *u_r_*(*t*). Note that *k_t_*, *d_t_*, and *m_t_* differ between the ACC-based and tactile-based measurement setups, due to difference in applied contact pressure and the addition of the PDMS layer in the tactile-based measurement. Assuming that the difference in *k_t_*, *d_t_*, and *m_t_* is relatively negligible, Equations (15) and (16) explains why *A_icf_/A*_1*cf*_ in the ACC-based measurement is consistently smaller than that in the tactile-based measurement for all higher harmonics, as shown in Figure 14, since $m_{s}\omega_{u_{r}}^{2}$ increases much faster with harmonic order than $k_{s}+jd_{s}\omega_{u_{r}}$.

Table 4a lists the normalized harmonic amplitudes, *A_icf_*/*A*_1*cf*_ obtained from the ACC-based and tactile-based measurements for all three subjects. As shown in Table 4a, this observed difference in *A_icf_*/*A*_1*cf*_ between the ACC-based and tactile-based measurements in Figure 14 is consistent across all three subjects. Table 4b presents the means and standard deviations (SD) of the absolute harmonic amplitudes, *A_icf_*, obtained from the two measurement modalities for all three subjects. Because *k_s_* is much larger than $m_{s}\omega_{u_{r}}^{2}$ [67,68], *A_icf_* is substantially higher in the ACC-based measurements than that in the tactile-based measurements for all three subjects.

Notably, although the SD of each harmonic in the tactile-based measurements is markedly smaller than that in the ACC-based measurements for all three subjects, the SD-to-mean ratios of all harmonics except the first are smaller in the ACC-based measurements than in the tactile-based measurements. According to the SDOF model, larger MA in the ACC-based measurements induces greater TVSP variation, which leads to greater distortion of the APW and larger fluctuations in the extracted IA. Because SD is calculated from the fluctuations in IA, the larger SD observed in the ACC-based measurements is consistent with greater TVSP-induced distortion. However, the SD-to-mean ratio is also influenced by the mean IA, which is substantially larger in the ACC-based measurements. Therefore, the smaller SD-to-mean ratios do not necessarily indicate less distortion. In addition, the higher sensor noise relative to the pulse-induced component in the ACC-based measurements may further affect the accuracy of the extracted IA and, consequently, IF and IIP, potentially affecting the derived time-based arterial indices HR, HRV, RR, and RM to varying extents.

Finally, in addition to the differences in harmonic amplitudes discussed above, other factors may contribute to the discrepancies between the two measurement modalities, as discussed below:

(1) TCS stack influence on the arterial pulse signal *u_r_*(*t*): During pulse measurement, the TCS stack acts as a local disturbance to arterial pulse propagation in the CV system, thereby altering the local arterial pulse signal *u_r_*(*t*). However, the effect of the TCS stack on *u_r_*(*t*) at the CA is omitted in the above evaluation. While this effect of the TCS stack on *u_r_*(*t*) is expected to differ between the ACC-based and tactile-based measurements, quantifying its precise impact remains difficult.
(2) System parameter variations in the TCS stack: Differences in *m_t_*, *k_t_*, and *d_t_* exist between the two measurement systems. Figure 15 compares the sensor-noise frequency spectra between the two modalities. The first peak corresponds to the residual of the first harmonic of the pulse signal. The second peak, circled in Figure 15, represents the natural frequency of the TCS stack, which varies with MA. The natural frequency of the TCS stack with the tactile sensor is slightly lower than that with the analog ACC. This observation is consistent with findings in the literature showing that increased contact pressure reduces the natural frequency of a soft medium, such as the tissue overlying the artery. Furthermore, classical vibration theory indicates that the first, second, and third harmonics may be amplified by the TCS stack relative to the higher harmonics. As shown in Figures S12 and S18 of the Supplementary Materials for Subjects #6 and #7, respectively, the sensor-noise peak differs between the two modalities, occurring at a slightly lower frequency for the ACC-based measurement than for the tactile-based measurement.

#### 3.5.3. Extracted HR, HRV, RR, and RM and Frequency Spectra of the IF

Table 5 compares the extracted IF-based and IIP-based arterial indices between ACC-based and tactile-based measurements across all three subjects. As established in Part I, IIP-based respiration rate (RR) and respiratory modulation (RM) provide higher accuracy than their IF-based counterparts due to the lower sensitivity of IIP to MA. Consequently, comparative evaluation focuses on the IIP-based estimates. The extracted RR and RM show noticeable discrepancies between measurement modalities, with the difference in IIP-based RM being particularly pronounced: tactile-based measurements yielded substantially lower IIP-based RM values across all subjects (0.0172 vs. 0.0239 for #6; 0.0308 vs. 0.0635 for #7; 0.0070 vs. 0.0144 for #8). In contrast, variations in HRV and IIP-based RR were mixed across subjects. These discrepancies likely stem from differing MA profiles between setups and the additional acceleration-sensing term present in the IF signal.

Figure 16 compares the frequency spectra of the IF from the first three harmonics, *f_ai_*(*t*)/*i* for the ACC-measured signal and *f_i_*(*t*)/*i* for the tactile-measured signal (*i* = 1, 2, and 3). Physiologically, the frequency spectra across these three harmonics are expected to be identical; discrepancies among them stem from measurement-related errors, such as MA and sensor noise. Because the ACC-measured pulse signal in Subject #8 contains relatively small MA, the inter-harmonic spectral variations are smaller for the ACC-based measurement than for the tactile-based measurement. Although both modalities share very similar overall spectral profiles, the peak frequency shifts slightly lower in the ACC-measured signal, whereas its spectral amplitude nearly doubles relative to the tactile counterpart. This frequency shift aligns with the theoretical prediction in Part I that acceleration sensing introduces an additional term into the IF.

As shown in Figures S13 and S19 of the Supplementary Materials for Subjects #6 and #7, respectively, higher MA in the ACC-measured signals causes larger discrepancies among the three harmonic spectra, whereas lower MA in the tactile-measured signals yields minimal inter-harmonic spectral differences. Furthermore, the spectral patterns for Subjects #6 and #7 consistently exhibit a slight frequency shift and an amplitude shift between modalities, further supporting the presence of the acceleration-induced term in the IF for ACC-based measurements. Notably, the overall spectral profiles also show inter-subject variability across the three participants.

## 4. Discussion

As established in Section 3, digital ACC measurements generally exhibit a lower ratio of *a_tf_*/(*a_tvsp_* + *a_noise_*) than analog ACC measurements. Consequently, the following discussion focuses primarily on the analog ACC measurements and their comparison with tactile-based measurements. The experimental findings, together with the theoretical results established in Part I, indicate that the differences between ACC- and tactile-based arterial pulse measurements can be understood in terms of two practical challenges and two fundamental measurement differences. The practical challenges concern the implementation of ACC-based measurement: (1) achieving firm (or stable) sensor fixation and (2) the acceleration-sensing nature of the ACC, which permits arterial indices to be extracted only from the measured acceleration waveform, *APW_a_*, rather than from the displacement waveform, APW. The fundamental differences arise from the underlying dynamics of acceleration sensing and the TCS stack: (1) differences in the IF-based arterial indices and the associated IF spectra resulting from acceleration sensing and (2) differences in the normalized harmonic amplitude distributions resulting from variations in the TCS stack. The practical challenges primarily affect the feasibility and reliability of pulse acquisition, whereas the fundamental differences affect the measured signal characteristics and derived arterial indices. The first fundamental difference was established and discussed in detail in Part I, whereas the second becomes evident in Section 2 through application of the Part I framework and is experimentally validated in the present study.

### 4.1. Radial, Lateral and Axial Wall Motions at the CA in ACC-Based Measurements

The reconstructed APWs in Figure 8 and Figure S1–S3 show substantial differences among radial, lateral, and axial arterial wall motions. Radial wall motion, represented by *z*(*t*), has the largest amplitude in all five subjects. The lateral displacement, *y*(*t*), is smaller than *z*(*t*), and its amplitude and waveform vary considerably across subjects. In contrast, the axial displacement, *x*(*t*), is not only smaller than *z*(*t*) but also exhibits a markedly different pulse waveform. These observations remain consistent across the five subjects.

The observed lateral motion can be attributed largely to radial wall motion resulting from off-center alignment of the ACC with the CA. The variation in *y*(*t*) among subjects suggests that even minor differences in ACC off-center alignment can significantly affect the measured lateral wall motion. Conversely, the smaller amplitude and distinct waveform of *x*(*t*) indicate that axial wall motion is mechanically distinct from radial wall motion.

The difficulty in measuring axial wall motion can be understood through the measurement configuration. Achieving reasonably good alignment between the ACC and the CA axis is challenging due to the anatomical complexity of the CA. Consequently, radial and lateral wall motions can readily contaminate the measured axial acceleration. Furthermore, while radial wall motion is constrained by the surrounding tissue outside the TCS stack, this tissue is not directly aligned with the direction of radial motion. In contrast, the surrounding tissue directly constrains axial wall motion—a constraint expected to increase with contact pressure. These mechanical differences explain both the suppressed axial wall motion observed in this study and the wide variation in axial pulse waveforms reported in imaging-based measurements using alternative dedicated instruments.

Overall, these results indicate that radial wall motion is the most prominent and practically measurable component of CA wall motion when using the ACC. Conversely, axial wall motion is substantially more difficult to measure reliably due to strict anatomical alignment requirements and stronger mechanical constraints imposed by the surrounding tissue. Although axial wall motion exhibits a distinct waveform from radial wall motion, the present study does not establish specific physiological markers that are uniquely encoded in axial motion or cannot be sufficiently inferred from radial measurements. Therefore, the potential value of axial sensing as a complementary approach remains to be established through further investigation.

### 4.2. Extracted Arterial Indices Based on APW_a_ at the CA in ACC-Based Measurement

#### 4.2.1. Ratio of *b*/*a*, Δ*t_a_*_−*b*_, and Normalized Harmonic Amplitude Distribution

The *b*/*a* ratio and Δ*t_a_*_−*b*_ provide complementary information on the morphology of *APW_a_*. The *b*/*a* ratio is an amplitude-based feature and is therefore more sensitive to measurement conditions, whereas Δ*t_a_*_−*b*_ is a time-based feature determined primarily by the temporal characteristics of the pulse waveform. Related amplitude-ratio and timing features have been reported in previous arterial pulse-wave studies, although direct numerical comparison should be made cautiously because measurement sites and sensing configurations are not identical.

For the eight subjects measured using the analog ACC, the *b*/*a* ratio showed greater inter-subject variation than Δ*t_a_*_−*b*_. The variation in the *b*/*a* ratio is expected because the measured pulse amplitude is affected by the TCS stack, which varies among subjects. Although the SDOF-TF method substantially removes TVSP-induced distortion and sensor noise, residual errors inevitably remain in the extracted *APW_a_*. Thus, the remaining variation in the *b*/*a* ratio may reflect subject-dependent TCS characteristics and residual measurement errors, in addition to physiological differences. In contrast, the relatively smaller variation in Δ*t_a_*_−*b*_ supports its greater robustness as a timing-based index for ACC-based pulse measurement.

The normalized harmonic amplitude distribution provides a key mechanism for evaluating waveform morphology while mitigating subject-dependent transfer functions of the TCS stack. As demonstrated in Table 3, the normalized harmonic amplitudes consistently decrease with increasing order across subjects, reflecting a dominant fundamental frequency component. Minor variations in these normalized harmonic amplitudes among individuals—particularly in subjects with higher BMI—highlight how unique anatomical profiles within the TCS stack can influence frequency-dependent transmission dynamics. Overall, despite these subject-specific mechanical coupling differences, normalizing the harmonic amplitudes successfully isolates relative spectral features and underscores the consistency of the primary physiological waveform characteristics across subjects.

#### 4.2.2. Beat-Based HR and HRV Verus IF-Based HR and HRV

The consistency between beat-based HR—determined from pulse-wave peaks or valleys—and IF-based HR indicates that the dominant cardiac frequency is preserved in ACC-based measurements. In contrast, HRV is more sensitive and thus more susceptible to measurement-related effects, namely, the TCS stack dynamics, MA, and sensor noise. The observed differences between beat-based and IF-based HRV reflect varying levels of error embedded in their respective calculation approaches.

#### 4.2.3. IF-Based RR and RM Versus IIP-Based RR and RM

As established in Part I, acceleration sensing introduces an additional term to the IF, but whether acceleration sensing similarly introduces an additional term into IIP and affects related temporal features remains unclear. As shown in Table 2b, IF-based RR is lower than IIP-based RR, with the latter being within or very close to the norminal at-rest RR range. In the meantime, IIP-based RM is below 0.1 for all the subjects, which is aligned with the related findings in our previous study on finger PPG signals [59]. It is interesting to note that the IIP-based RR and RM from tactile-based measurement here reveal the same difference from IF-based RR and RM in a previous study [46].

### 4.3. Comparison Between ACC-Based and Tactile-Based Measurements

#### 4.3.1. MA and Effects of TVSP-Induced Distortion and Sensor Noise on Reconstructed APW

Compared with tactile-based measurement, ACC-based measurement is more susceptible to MA, primarily because firm fixation of the ACC to the skin is more difficult to achieve using medical tape. In addition, the two measurement configurations differ in their control of sensor–skin contact conditions. The tactile sensor was held manually, allowing its position and contact force to be adjusted to optimize pulse acquisition, whereas tape fixation of the ACC did not allow adequate control of contact pressure or skin conformity. These differences may have contributed to the observed signal-quality differences and represent potential confounding factors in the direct comparison between ACC- and tactile-based measurements. The resulting relative motion between the ACC and the CA can introduce substantial low-frequency components (i.e., MA) into the measured acceleration signal. Furthermore, In addition, because the acceleration pulse signal occupies only a small portion of the ACC’s measurement range, TVSP-induced distortion and sensor noise can have a substantial effect on the measured signal. Although the SDOF-TF method substantially reduces these effects in the extracted APW_a_, residual errors are expected to remain in the extracted pulse waveform.

As a result, the peak and valley features of the extracted APW_a_, which correspond to relatively prominent features of individual pulse cycles, are generally more reliable than other waveform features. Nevertheless, their amplitudes and exact locations can still be affected by residual errors. In contrast, reconstruction of the displacement-domain APW from the extracted APW_a_ requires double integration, which substantially increases the influence of residual errors. The reconstructed APW therefore exhibits significant distortion and is not sufficiently reliable for extraction of arterial indices. This difference represents an important limitation inherent to ACC-based measurement that does not exist in tactile-based displacement measurement.

#### 4.3.2. Normalized Harmonic Amplitude Distribution

The absolute pulse amplitude measured by either sensor does not carry strong physiological implications because it depends on the TCS stack and the degree of sensor alignment with the artery. Consequently, differences in absolute pulse amplitude between ACC-based and tactile-based measurements cannot be directly interpreted as differences in arterial pulse strength.

For the same reason, the absolute harmonic amplitudes in the two measurement modalities are not directly comparable. The TCS stacks differ substantially between the ACC and tactile sensor, and the interaction between the artery and the TCS stack also depends on sensor type, contact pressure, and measurement configuration. These differences can modify the transmission of individual harmonic components and thereby substantially alter the measured harmonic amplitude distribution. Thus, the absolute harmonic amplitudes obtained from the two modalities are influenced by the combined artery–TCS–stack dynamics to different extents compared to the unperturbed arterial pulse signal.

However, the normalized harmonic amplitude distribution removes the overall amplitude scaling associated with the measurement system and thus provides more meaningful physiological information. Nevertheless, its interpretation still requires consideration of the TCS transfer characteristics. In particular, the substantial difference in normalized harmonic amplitude distributions observed between ACC-based and tactile-based measurements indicates that the measurement modality itself can alter the apparent spectral composition of the arterial pulse. Therefore, normalized harmonic amplitudes may be useful for physiological assessment within a given measurement modality and under consistent measurement conditions, whereas direct comparison between modalities should be approached cautiously.

#### 4.3.3. Extracted HR, HRV, RR, RM, and Frequency Spectrum of the IF

HR was generally consistent between ACC-based and tactile-based measurements, indicating that the dominant cardiac periodicity is preserved despite differences in sensing principles and conditions. In contrast, IF-based HRV showed moderate differences between the two modalities. From a time-frequency perspective, HRV tracks non-stationary, time-varying frequency shifts across cardiac cycles, rendering it inherently more sensitive to measurement-related effects than HR. This divergence is further consistent with the theoretical finding in Part I that acceleration sensing introduces an additional term into the IF.

To examine this more closely, the frequency spectra of the IFs of the first three harmonics reveal how motion artifact (MA) and acceleration sensing interact. For Subject #8, where MA is minimal in both measurements, the frequency spectra of the three harmonics are closely aligned. In contrast, for Subjects #6 and #7, MA in the ACC measurement is substantial, causing large variations in the frequency spectra among the three harmonics.

Beyond these MA-induced variations, fundamental differences emerge when comparing ACC-based and tactile-based measurements. Specifically, the difference in the frequency spectra of the IF between the ACC and tactile measurements is driven by acceleration sensing introducing an additional term to the IF. In Subject #8—where MA is minimal—the overall spectral profiles remain very similar, but the peak frequency shifts slightly lower in the ACC-measured signal, its spectral amplitude nearly doubles relative to the tactile counterpart, and low-frequency spectral patterns differ between the two measurements. Similarly, for Subjects #6 and #7, differences in spectral patterns in the IF are observed between the ACC and tactile measurements. These modality-dependent differences provide direct experimental evidence of the acceleration-induced term altering the measured IF and its temporal variation.

Compared with HRV, IIP-based RR and RM showed greater differences between ACC-based and tactile-based measurements, with RM exhibiting the most substantial difference. These differences can be attributed to the combined effects of MA and the TCS stack dynamics, and differences in the measured pulse waveform between the two sensing modalities. In particular, MA was generally larger in ACC-based measurements, making waveform features used to determine RR and RM more susceptible to measurement-related errors. The tactile sensor, which is firmly coupled to the CA through controlled contact pressure, is less susceptible to this type of sensor motion and therefore provides more reliable measurements of these indices. Overall, HR is relatively robust to the choice of measurement modality, whereas HRV, RR, and particularly RM are more sensitive to the measurement system and its associated artifacts.

### 4.4. Key Findings of ACC-Based Measurement in Comparison with Tactile-Based Measurement

The findings demonstrate that radial wall motion is the most feasible direction for ACC-based arterial pulse measurement, whereas axial wall motion presents substantial challenges for reliable measurement. Lateral wall motion provides a useful indicator of off-center misalignment between the ACC and the CA and may potentially be used for pulse-amplitude correction. Among the two ACCs investigated, the analog ACC provides higher-quality pulse signals than the digital ACC.

Pertaining to radial wall motion, ACC-based measurement presents the following technical challenges compared with tactile-based measurement:

(1) Sensor fixation and subject motion: ACC-based measurement places a more stringent requirement on the subject remaining still because medical tape alone cannot provide sufficiently firm fixation of the ACC at the CA. Therefore, subject motion can more readily introduce MA into ACC-based measurements.
(2) Selection of the pulse waveform for arterial-index extraction: Because of TVSP-induced distortion, sensor noise, and the fact that the acceleration waveform fluctuates around zero over most of a pulse cycle, only the measured APW_a_ is suitable for arterial-index extraction. In contrast, the displacement-domain APW reconstructed from APW_a_ accumulates residual measurement errors and is therefore not sufficiently reliable for arterial-index extraction.
(3) Reliability of *b*/*a* ratio and Δ*t_a_*_−*b*_: For the *b*/*a* ratio and the time interval Δ*t_a_*_−*b*_ determined from features of APW_a_, the *b*/*a* ratio is less reliable than Δ*t_a_*_−*b*_ because MA and sensor noise affect IA more strongly than IF and IIP, in addition to the TCS stack. Consequently, the timing-based Δ*t_a_*_−*b*_ provides a more robust arterial index than the amplitude-based *b*/*a* ratio.
(4) Comparability of normalized harmonic amplitude distributions: The normalized harmonic amplitude distributions obtained from ACC-based and tactile-based measurements are not directly comparable because the TCS stacks of the two measurement modalities are substantially different, and the effects of the TCS stack on the arterial pulse signal at the CA also vary with the measurement configuration. Thus, differences in normalized harmonic amplitudes between the two modalities cannot be directly interpreted as physiological differences.
(5) Effects on HR, HRV, RR, RM, and the frequency spectrum of IF: Among HR, HRV, RR, and RM, only HR is consistent between ACC-based and tactile-based measurements; HRV and RR show relatively moderate differences, whereas RM shows a significant difference. The difference in HRV is consistent with the theoretical finding in Part I that acceleration sensing introduces an additional term into the IF. The frequency spectra of the IFs of the first three harmonics further demonstrate differences between ACC-based and tactile-based measurements, indicating that acceleration sensing affects not only the IF but also its frequency-domain variation. This provides additional experimental support for the theoretical prediction regarding the acceleration-sensing contribution to IF and its potential influence on HRV. Whether acceleration sensing similarly introduces an additional term into IIP remains unclear and requires further investigation. Furthermore, because MA is generally substantially larger in ACC-based measurement than in tactile-based measurement, RR and RM obtained from tactile-based measurement are expected to be more reliable than those obtained from ACC-based measurement.

Overall, these findings indicate that, although ACC-based measurement provides a simple and compact approach for measuring radial arterial wall motion, its practical reliability is more strongly affected by sensor fixation, acceleration-sensing characteristics, and residual measurement errors than tactile-based measurement.

### 4.5. Study Limitations

There are six major limitations in this study. First, the TCS stack is assumed to behave linearly. This assumption is reasonable when the ACC is firmly fixed at the CA with sufficient contact pressure, which can be validated by the absence of large fluctuations in a measured acceleration signal. However, medical tape can result in loose fixation in some measurements. Such loose fixation can be reflected by large fluctuations in the measured acceleration signals, which were not analyzed here. Nevertheless, the analyzed results from all three axes reveal consistent temporal patterns of arterial wall motion in three directions, supporting the effectiveness of the SDOF-TF method for removing MA and sensor noise. Note that this SDOF-TF method is built upon the assumption of a linear TCS stack, as shown in Part I. As such, the analyzed results from the measured acceleration signals with no large fluctuations support this assumption.

Second, only a small number of subjects were included in this study. This limited sample size restricts statistical power and the generalizability of the findings. However, the aim of this work is to examine measurement-related technical issues associated with ACC-based arterial pulse measurement in comparison with tactile-based measurement, rather than to establish population-level clinical findings. The detailed analysis of the measured pulse signals from the available subjects enabled an in-depth examination of the observed measurement-related factors and a comparison between ACC-based and tactile-based measurements. In particular, the practical challenges identified—including difficulty securing the ACC to minimize MA, and the limited pulse-signal amplitude relative to the ACC measurement range—may have broader technical implications, although their generalizability requires further investigation in larger and more diverse cohorts.

Third, the comparison between ACC- and tactile-sensor-based measurements was subject to practical constraints associated with the two sensor configurations. The ACC was secured to the skin using medical tape, whereas the tactile sensor was held manually, allowing its position and contact force to be adjusted to optimize pulse acquisition. Although manually holding the ACC could permit similar adjustments, the arterial pulse signal occupies only a small portion of the ACC measurement range, making it particularly susceptible to MA. Manual holding could therefore introduce larger MA than tape fixation. Conversely, tape fixation did not ensure adequate control of contact pressure or skin conformity. These differences in sensor fixation and contact conditions may have contributed to the observed differences in signal quality and extracted arterial indices, potentially confounding the direct comparison between the two measurement modalities. Future studies should improve ACC fixation and control of sensor–skin contact conditions to better distinguish the effects of sensing principles from those of measurement configuration. In addition, alternative mechanical decoupling strategies to mitigate axial-motion suppression were not investigated in this study and warrant further investigation. Alternative numerical integration and filtering approaches, including Kalman filtering and adaptive high-pass filtering, were also not evaluated. Their potential to suppress residual BD in the first harmonic while preserving the physiological pulse waveform warrants further investigation.

Fourth, sequential acquisition is a limitation of the experimental design. To account for spontaneous physiological variations, clinical studies involving multiple instruments often use synchronized measurements at different arterial sites [69]. However, differences in the overlying tissue among arterial sites can lead to variations in TCS system parameters. Although sequential measurements at the same CA avoid inter-site differences in the overlying tissue, which is one factor influencing TCS system parameters, they remain susceptible to spontaneous physiological variations and changes in other measurement conditions, including sensor position relative to the CA and contact pressure. Under the measurement conditions of this study, spontaneous physiological variations were not expected to be the primary contributors to the observed differences. Because relevant physiological variables were not independently monitored, the contributions of spontaneous physiological variations to the observed differences cannot be quantified from the available data. In addition, each measurement modality may introduce instrument-dependent biases in the extracted arterial indices [62,63,64,70,71,72,73]. Future studies should incorporate independent monitoring of relevant physiological variables and improved control of sensor positioning and contact pressure to better distinguish these sources of variation and assess their contributions to differences between the two measurement modalities.

Fifth, the effect of the TCS stack on the pulse signal in the artery cannot be quantified. Theoretically, the TCS stack, as a local disturbance along the CA, exerts interacting forces on the arterial wall at its location, which may alter or suppress the local arterial pulse signal. However, quantifying this effect would require knowledge of *m*_0_, *k*_0_, and *d*_0_ of the TCS stack, as well as the mechanical properties and geometries of the artery and the artery-TCS-stack coupled dynamic interaction, which is beyond the scope of the present study. Therefore, the pulse signal analyzed in this study should be considered the arterial pulse signal at the TCS location that may already be affected by the TCS stack. This limitation does not affect the findings of this study because the primary objective is to examine how MA and sensor-related effects influence the measured pulse signal, rather than to quantify the disturbance of the arterial pulse caused by the TCS stack.

Lastly, three time-based arterial indices, HRV, RR, and RM, were not independently validated against ECG and a reference respiratory signal, respectively. Therefore, the extracted values should be interpreted as arterial-pulse-derived indices rather than independently validated physiological measurements. Nevertheless, the primary objective of this study was to investigate the technical issues associated with ACC-based arterial pulse measurement in comparison with tactile-based measurement, rather than to establish the clinical accuracy of the derived CV indices. Although ECG measurements can be affected by factors such as electrode placement and MA [70,71,72,73], and respiratory monitoring methods may be subject to measurement errors, calibration requirements, and MA [62,63,64], these limitations do not eliminate the need for independent reference measurements. Future studies should incorporate appropriate ECG and respiratory reference methods, with consideration of their measurement uncertainties, to evaluate the accuracy and physiological significance of the derived indices.

## 5. Conclusions

This study experimentally investigated ACC-based arterial pulse measurement and compared it with tactile-based measurement. The results demonstrated that the analog ACC overall provides higher-quality pulse signals than the digital ACC. Three-axis measurements showed that radial wall motion corresponds well to the arterial pulse measured by the tactile sensor, whereas lateral wall motion can indicate off-center misalignment between the ACC and CA. Although ACC-based measurement offers a simpler approach than imaging methods for characterizing axial arterial wall motion, reliable characterization of axial wall motion for arterial indices remains challenging because of two substantial practical challenges identified experimentally.

Comparison with tactile-based measurement further revealed two practical challenges associated with ACC-based measurement: the need for firm sensor fixation and the inherent limitation of acceleration sensing, which directly provides the acceleration-domain pulse waveform, APW_a_, rather than the displacement-domain waveform, APW. In addition, ACC- and tactile-based measurements exhibit two fundamental differences. First, consistent with the theoretical analysis in Part I, acceleration sensing introduces an additional term into the IF of individual pulse harmonics, thereby affecting IF-based arterial indices and the frequency spectrum of IF. Second, differences in the TCS stacks substantially alter the measured normalized harmonic amplitude distribution between the two measurement approaches. Overall, ACC-based measurement provides a simple approach for CV assessment; however, its reliability depends strongly on adequate sensor fixation, and its acceleration-sensing nature introduces fundamental differences in the extracted arterial indices and harmonic characteristics. Tactile-based measurement therefore remains preferable for reliable acquisition of arterial pulse waveforms and accurate CV indices.

## Figures and Tables

**Figure 1:**
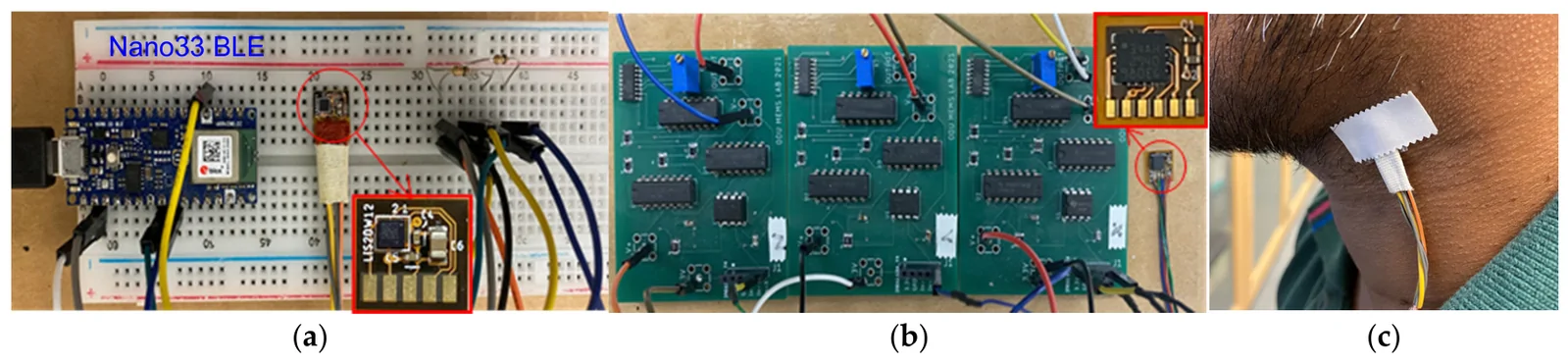
Measurement hardware for two ACCs: (**a**) a 3-axis digital ACC (LIS2DW12) mounted on a custom PCB and connected to an Arduino Nano 33 BLE microcontroller; (**b**) an analog ACC (LIS344ALH) mounted on a custom PCB and connected to the associated PCB-based circuits; and (**c**) fixation of an ACC at the CA using medical tape.

**Figure 2:**
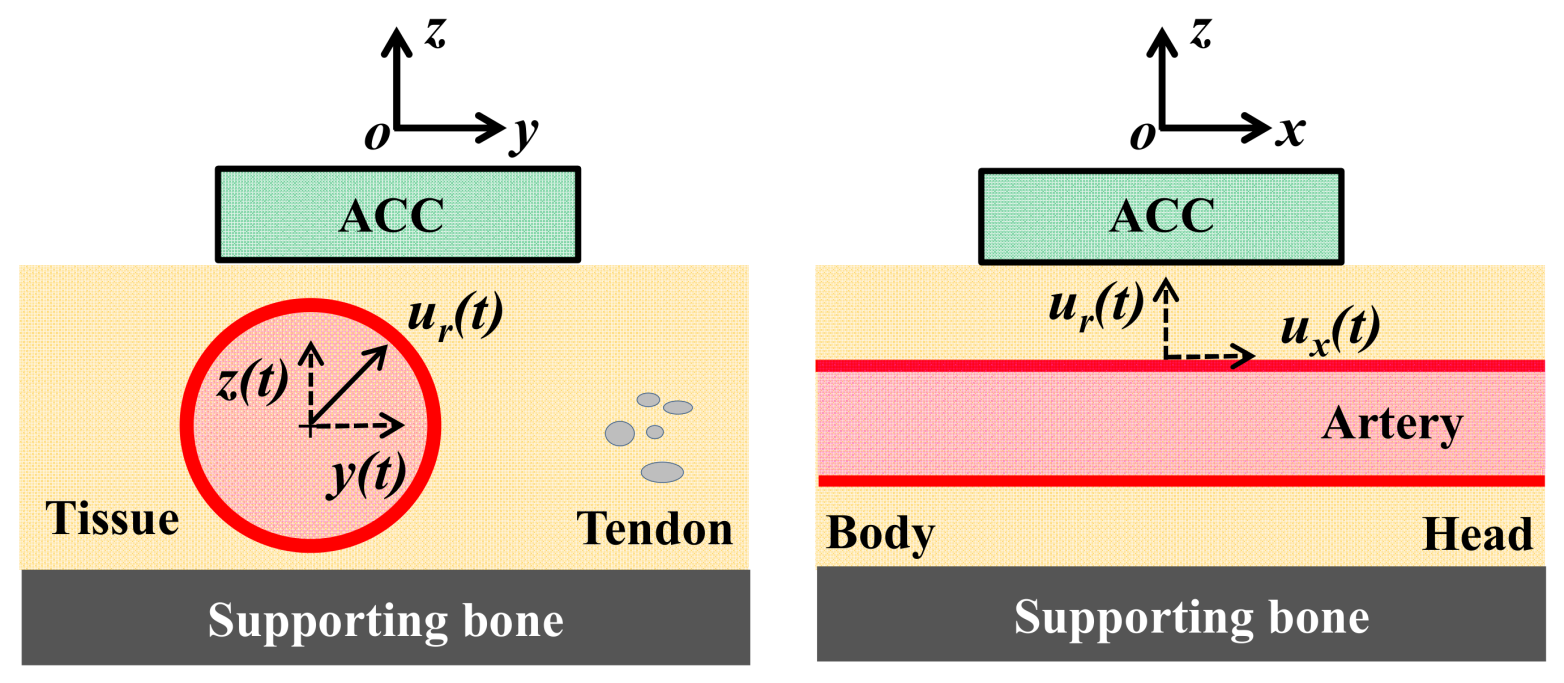
Schematic of the alignment of the ACC coordinate axes with the three arterial wall motion directions of the CA: *u_r_*(*t*) and *u_x_*(*t*) denote the radial and axial wall displacements of the CA, respectively; and the three ACC axes are *z*(*t*), corresponding to the radial direction; *y*(*t*), corresponding to the lateral direction; and *x*(*t*), corresponding to the axial direction.

**Figure 3:**
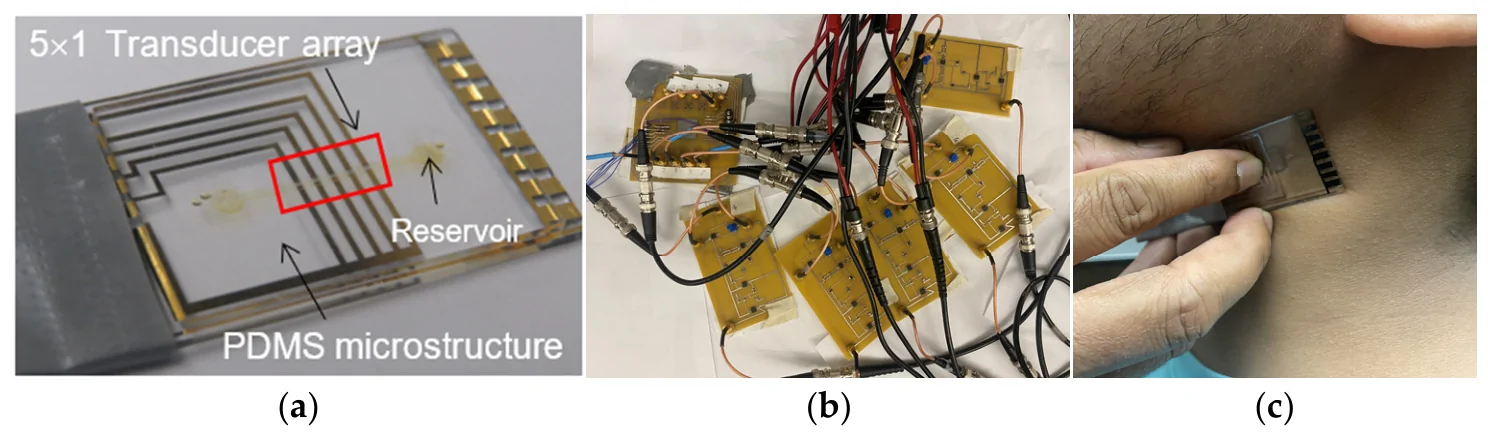
Measurement hardware for a micro-fabricated tactile sensor: (**a**) the tactile sensor; (**b**) associated PCB-based circuits; (**c**) fixation of the sensor at the CA using the fingers.

**Figure 4:**
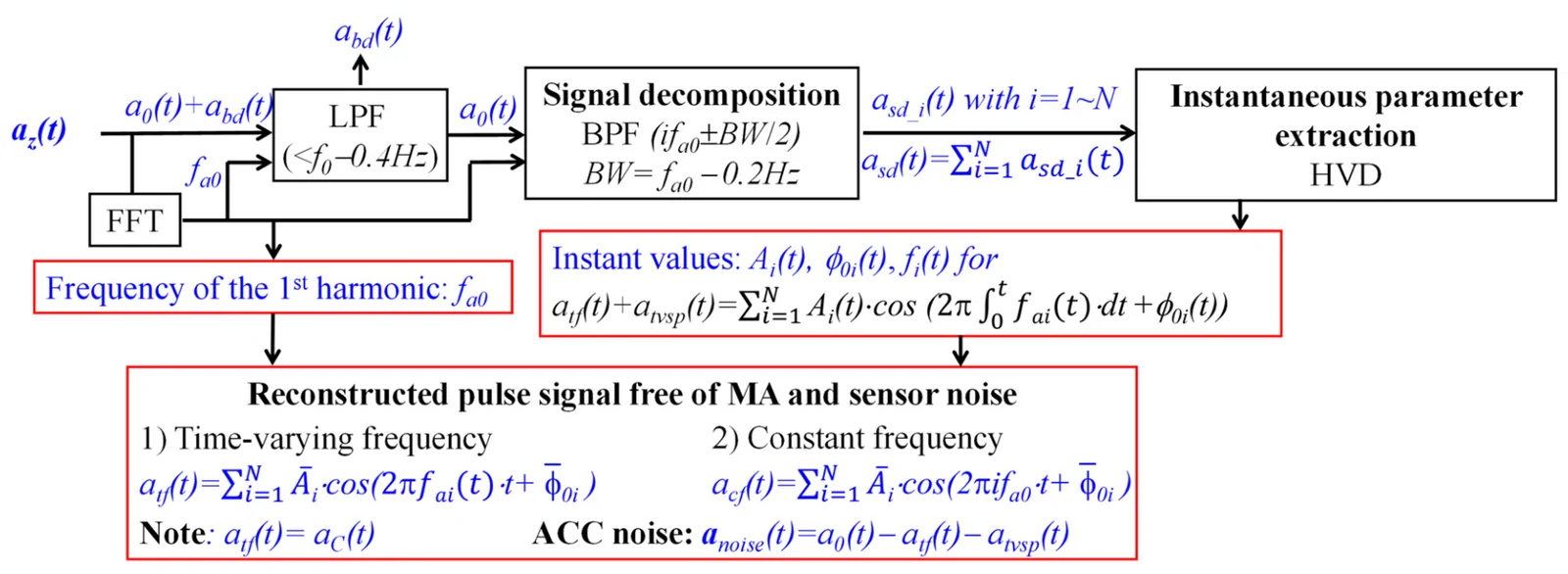
Signal-processing flowchart for time-frequency analysis (TFA) of a measured acceleration signal based on the SDOF-TF method.

**Figure 5:**
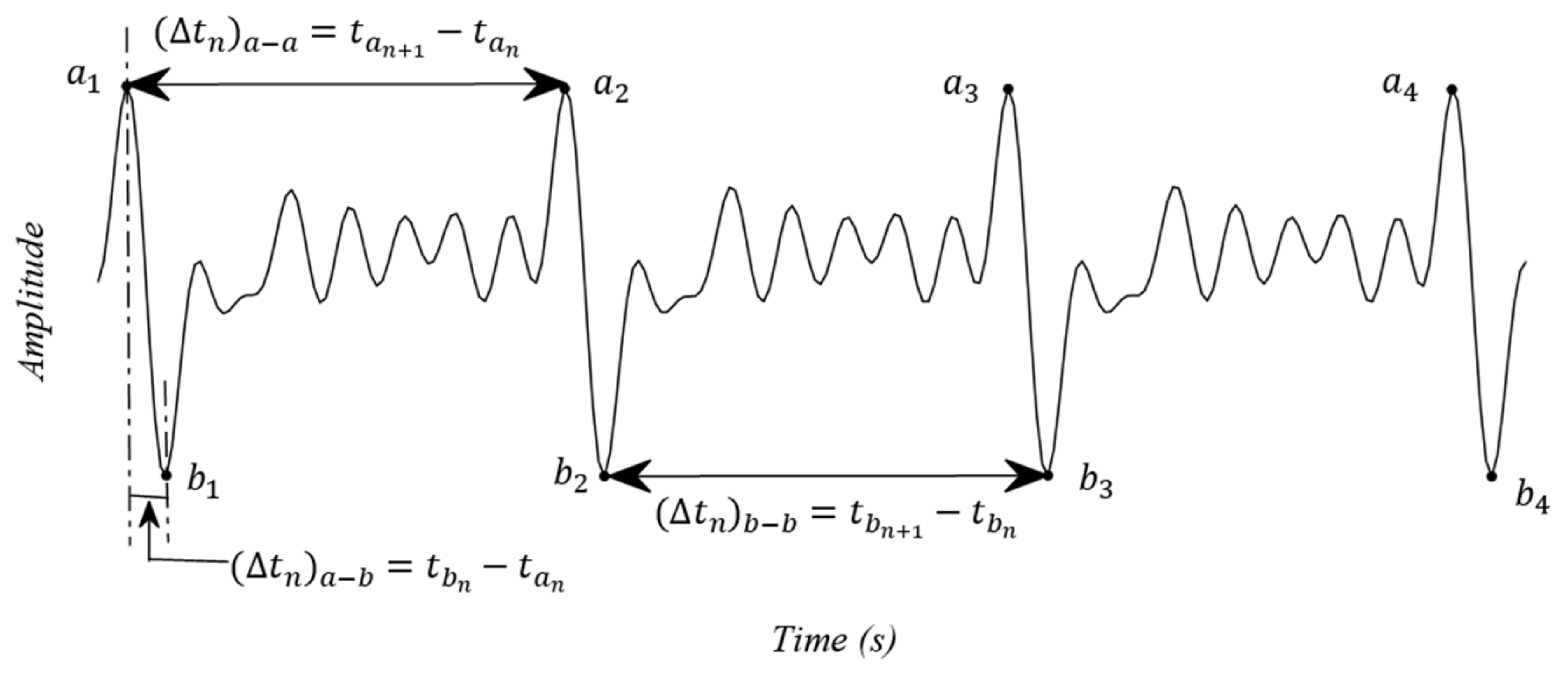
Features of the extracted *a_tf_*(*t*) used to derive arterial indices.

**Figure 6:**
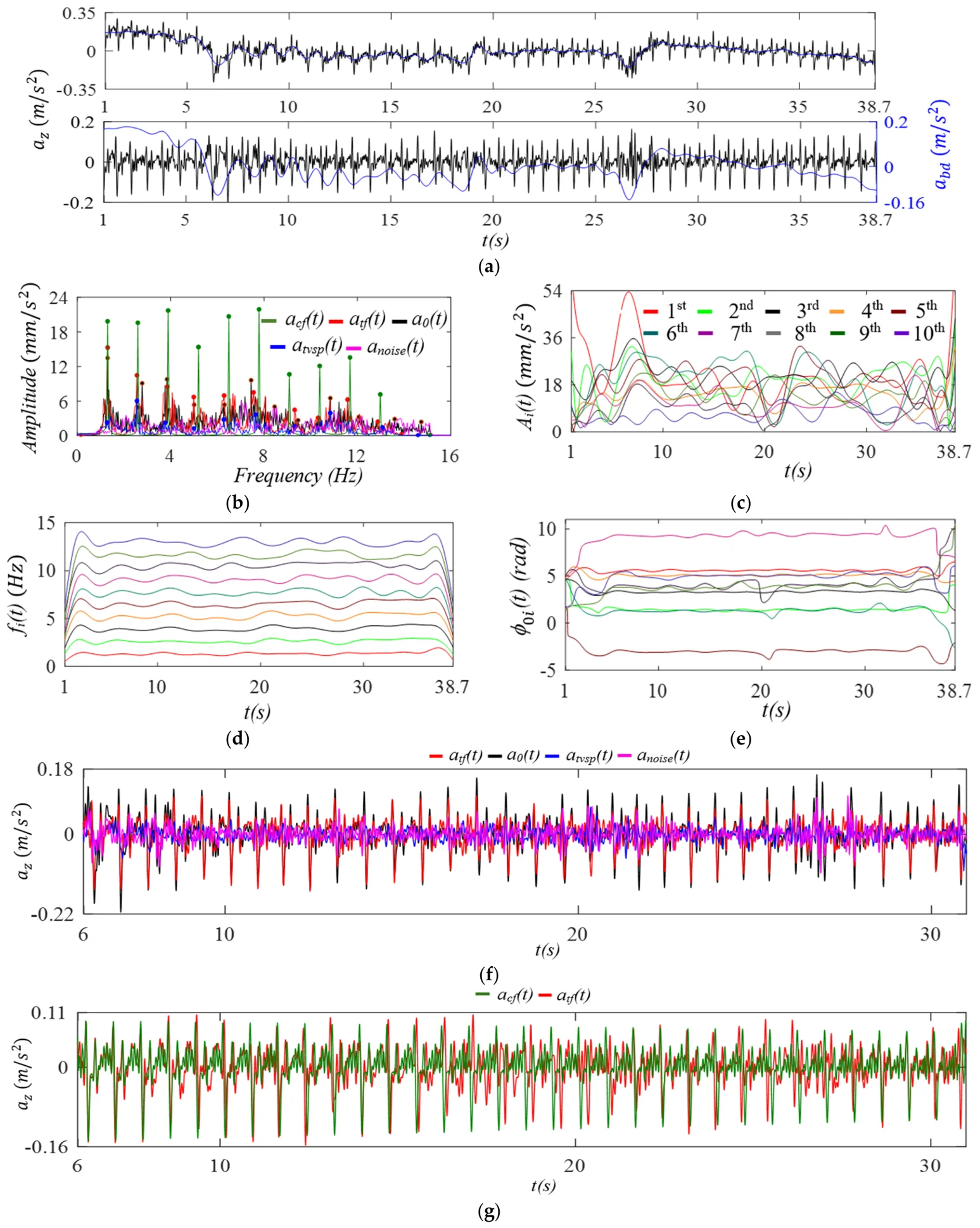

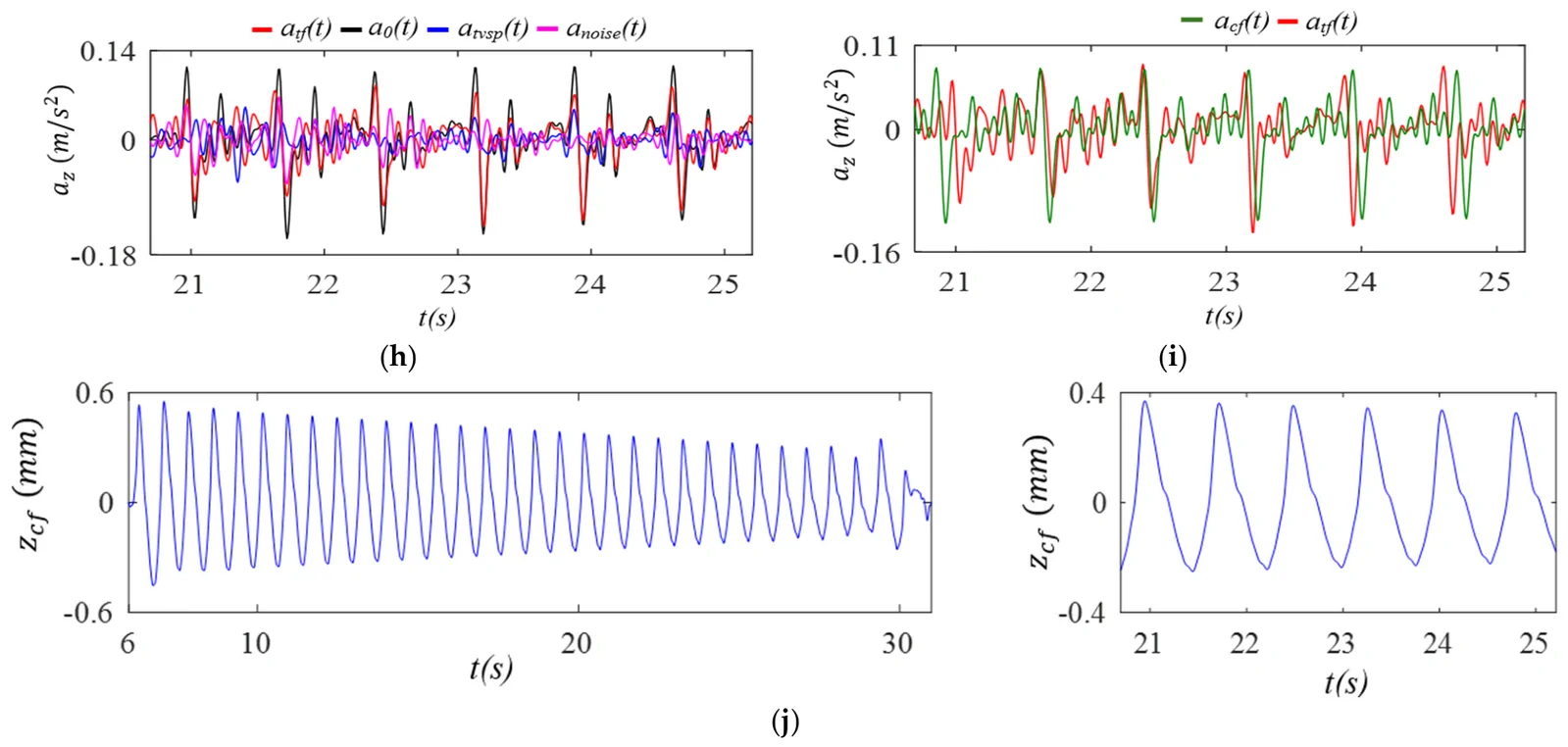
Measured acceleration signal of subject #2 using the digital ACC and its analyzed results: (**a**) originally measured acceleration signal *a_z_*(*t*) and the extracted baseline-drift acceleration *a_bd_*(*t*); (**b**) frequency spectra of different signal components in *a_z_*(*t*); (**c**) instant amplitude of *a_z_*(*t*); (**d**) instant frequency of *a_z_*(*t*); (**e**) instant initial phase of *a_z_*(*t*); (**f**) comparison of *a_z_*(*t*) with the extracted signal components; (**g**) comparison of *a_tf_*(*t*) with *a_cf_*(*t*); (**h**) an enlarged portion of the comparison of *a_z_*(*t*) with the extracted signal components; (**i**) an enlarged portion of the comparison of *a_tf_*(*t*) with *a_cf_*(*t*); and (**j**) extracted *z_cf_*(*t*) using Equation (11).

**Figure 7:**
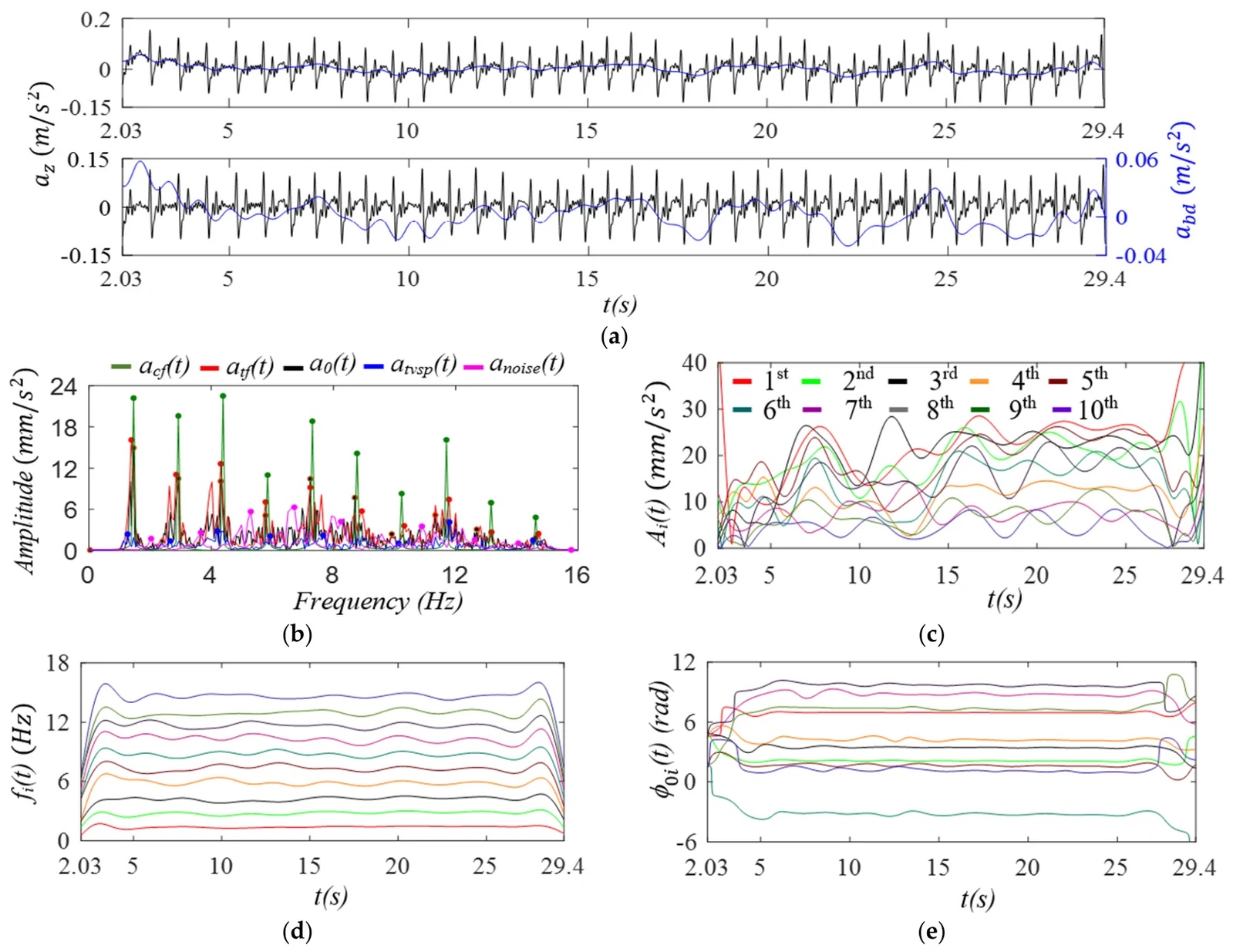

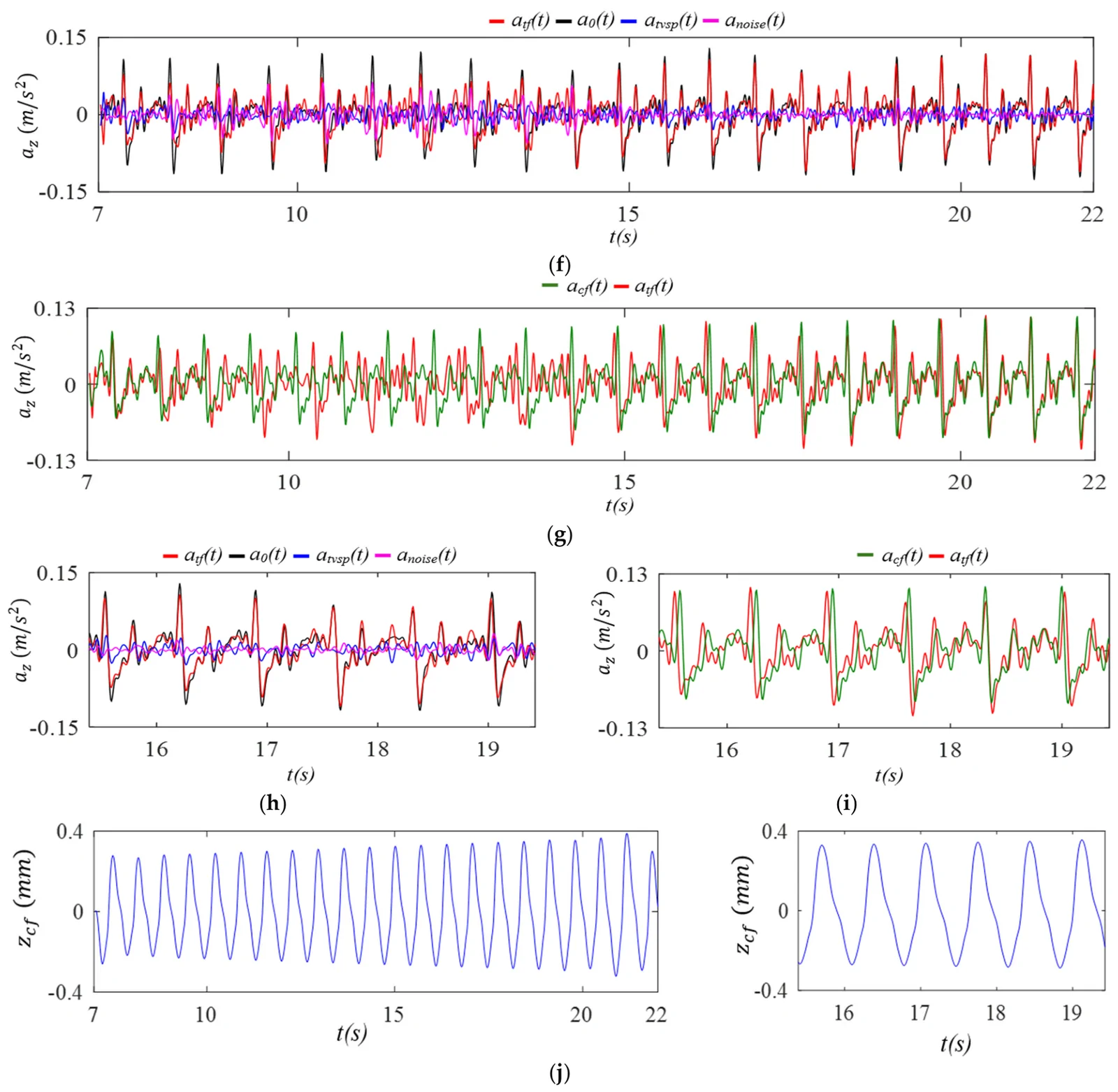
Measured acceleration signal of subject #2 using the analog ACC and its analyzed results: (**a**) originally measured acceleration signal *a_z_*(*t*) and the extracted baseline-drift acceleration *a_bd_*(*t*); (**b**) frequency spectra of different signal components in *a_z_*(*t*); (**c**) instant amplitude of *a_z_*(*t*); (**d**) instant frequency of *a_z_*(*t*); (**e**) instant initial phase of *a_z_*(*t*); (**f**) comparison of *a_z_*(*t*) with the extracted signal components; (**g**) comparison of *a_tf_*(*t*) with *a_cf_*(*t*); (**h**) an enlarged portion of the comparison of *a_z_*(*t*) with the extracted signal components; (**i**) an enlarged portion of the comparison of *a_tf_*(*t*) with *a_cf_*(*t*); and (**j**) extracted *z_cf_*(*t*) using Equation (11).

**Figure 8:**
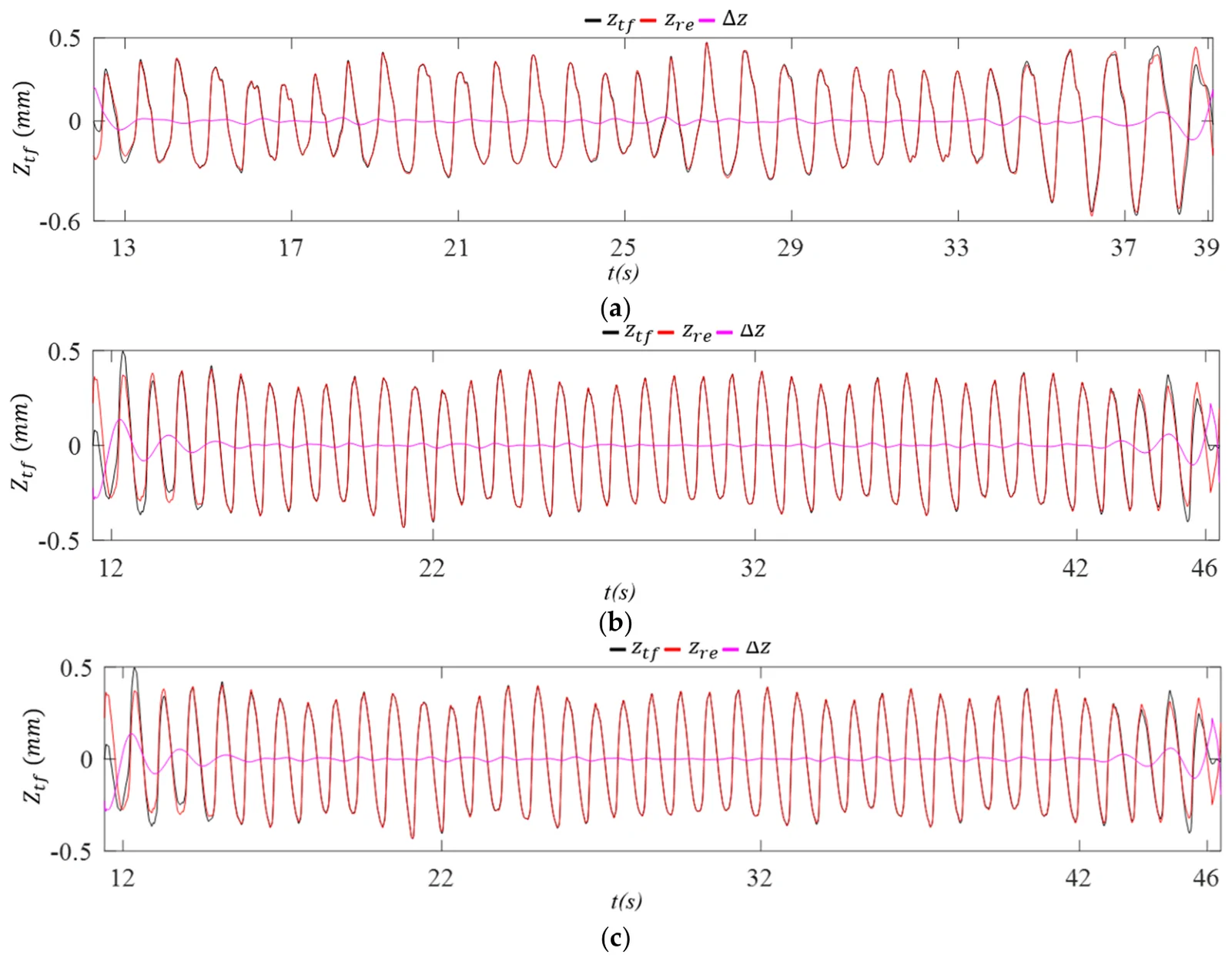
Comparison of the derived *z_tf_*(*t*) using Equation (10) and double integration using *cumtrapz* and *LPF*: (**a**) Subject #1; (**b**) Subject #7; (**c**) Subject #8.

**Figure 9:**
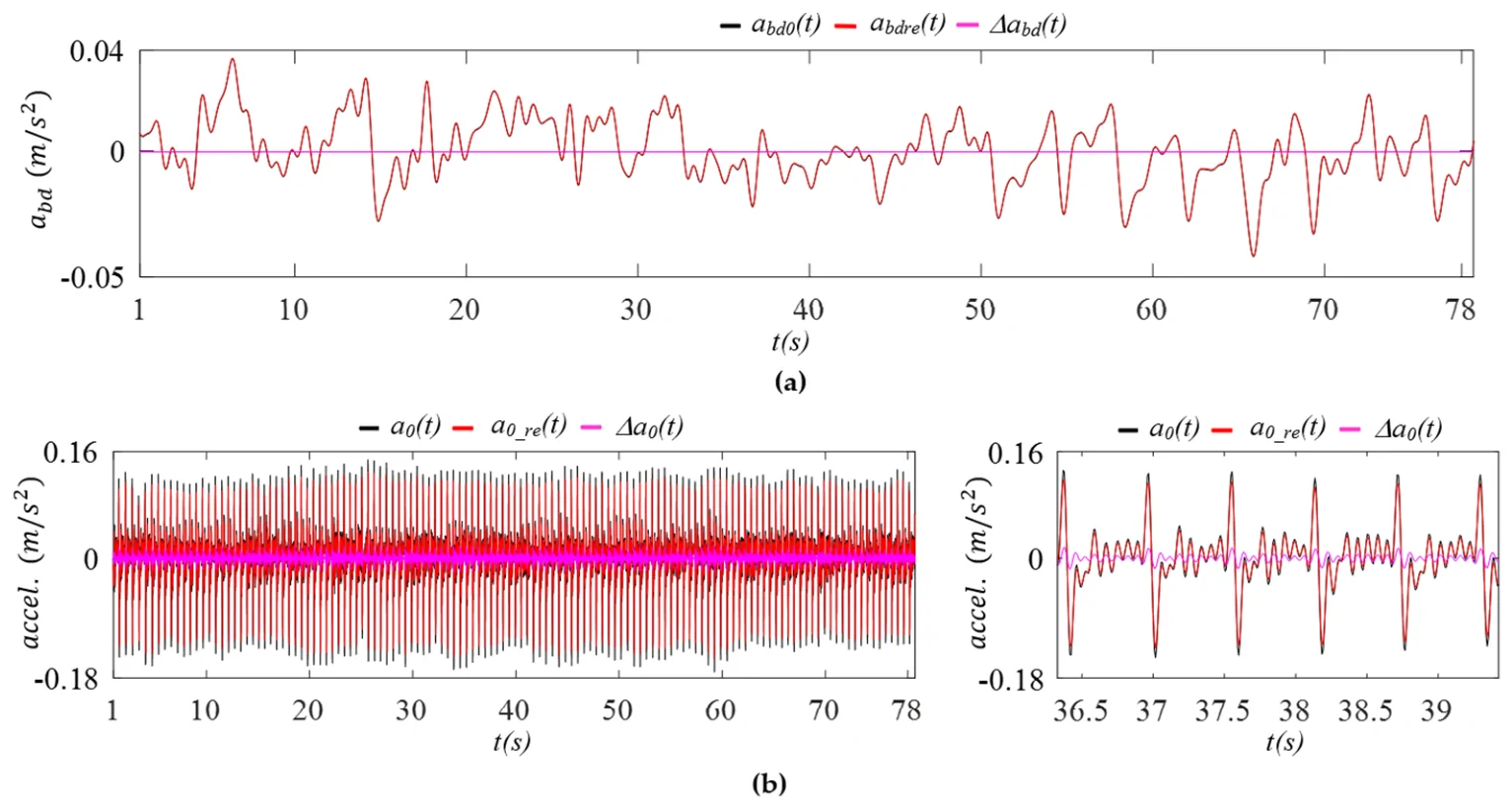
Comparison of (**a**) *a_bd_*(*t*), *a_bd_re_*(*t*), and Δ*a_bd_*(*t*); and (**b**) *a*_0_(*t*), *a*_0*_re*_(*t*), and Δ*a*_0_(*t*) of Subject #8 from the analog ACC measurement.

**Figure 10:**
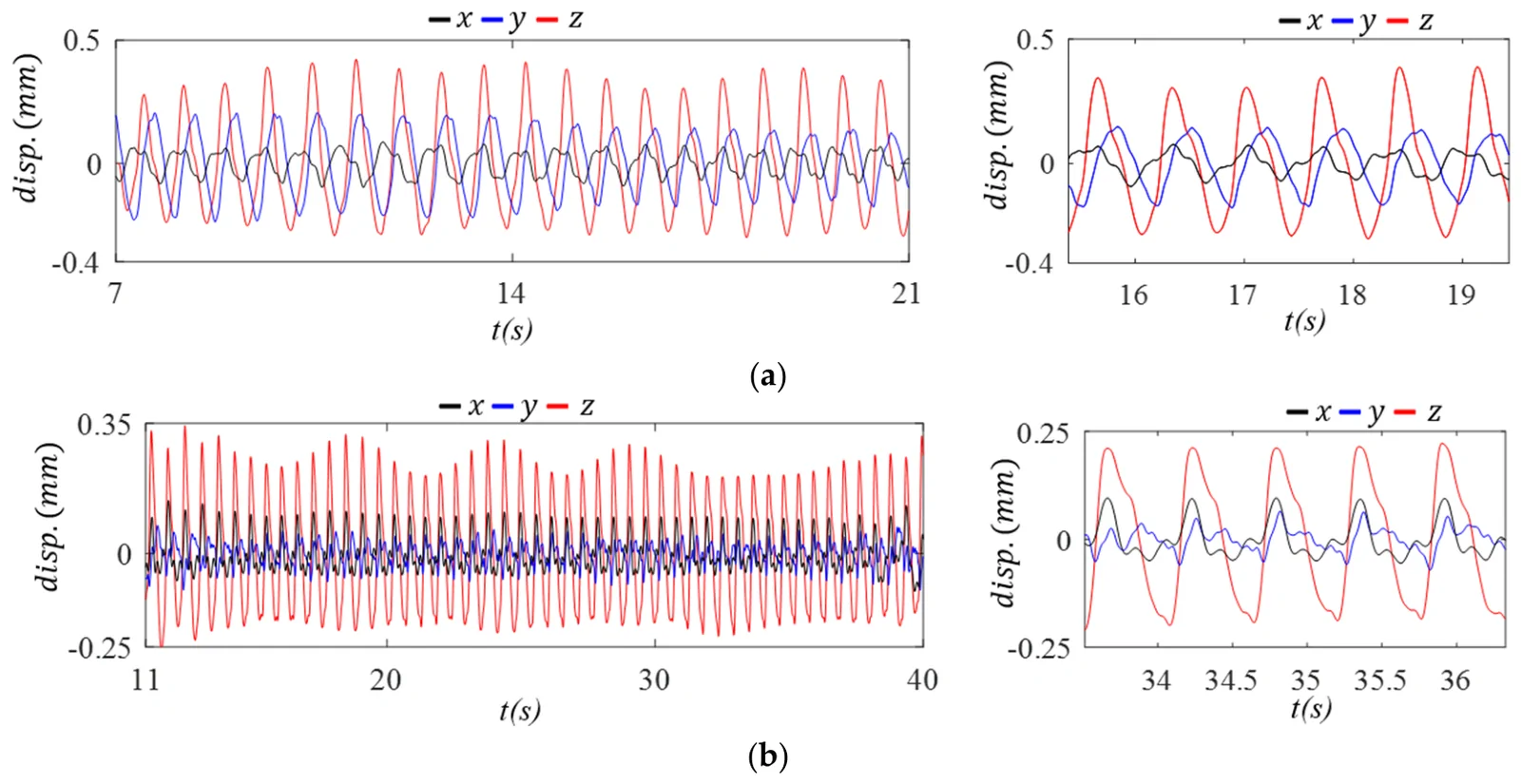
Extracted displacements (or APWs) along the three axes from the measured acceleration signals using the analog ACC: (**a**) Subject #2, (**b**) Subject #4.

**Figure 11:**
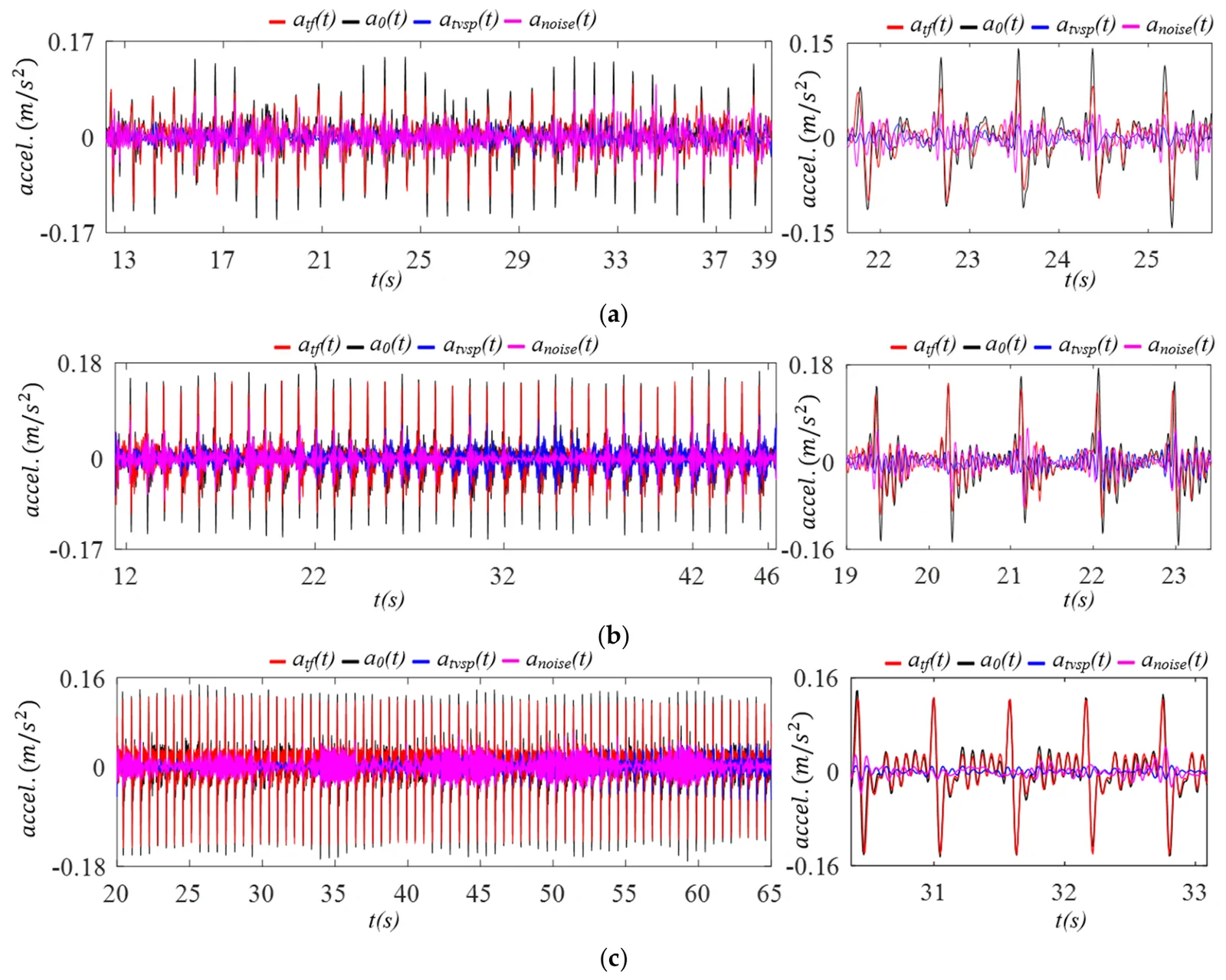
Comparison of *a*_0_(*t*), *a_tf_*(*t*), *a_tvsp_*(*t*), and *a_noise_*(*t*): (**a**) Subject #1, (**b**) Subject #7, (**c**) Subject #8.

**Figure 12:**
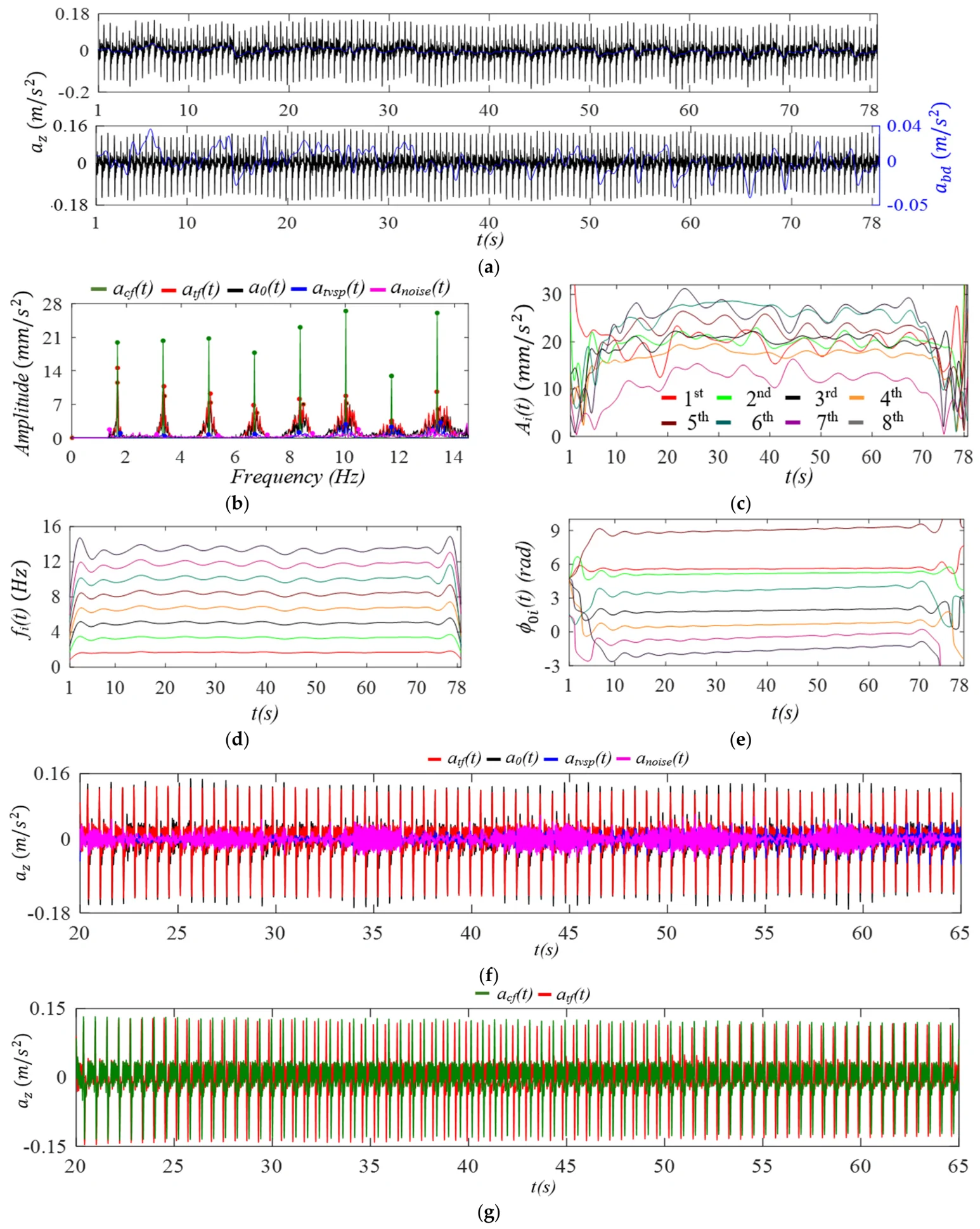

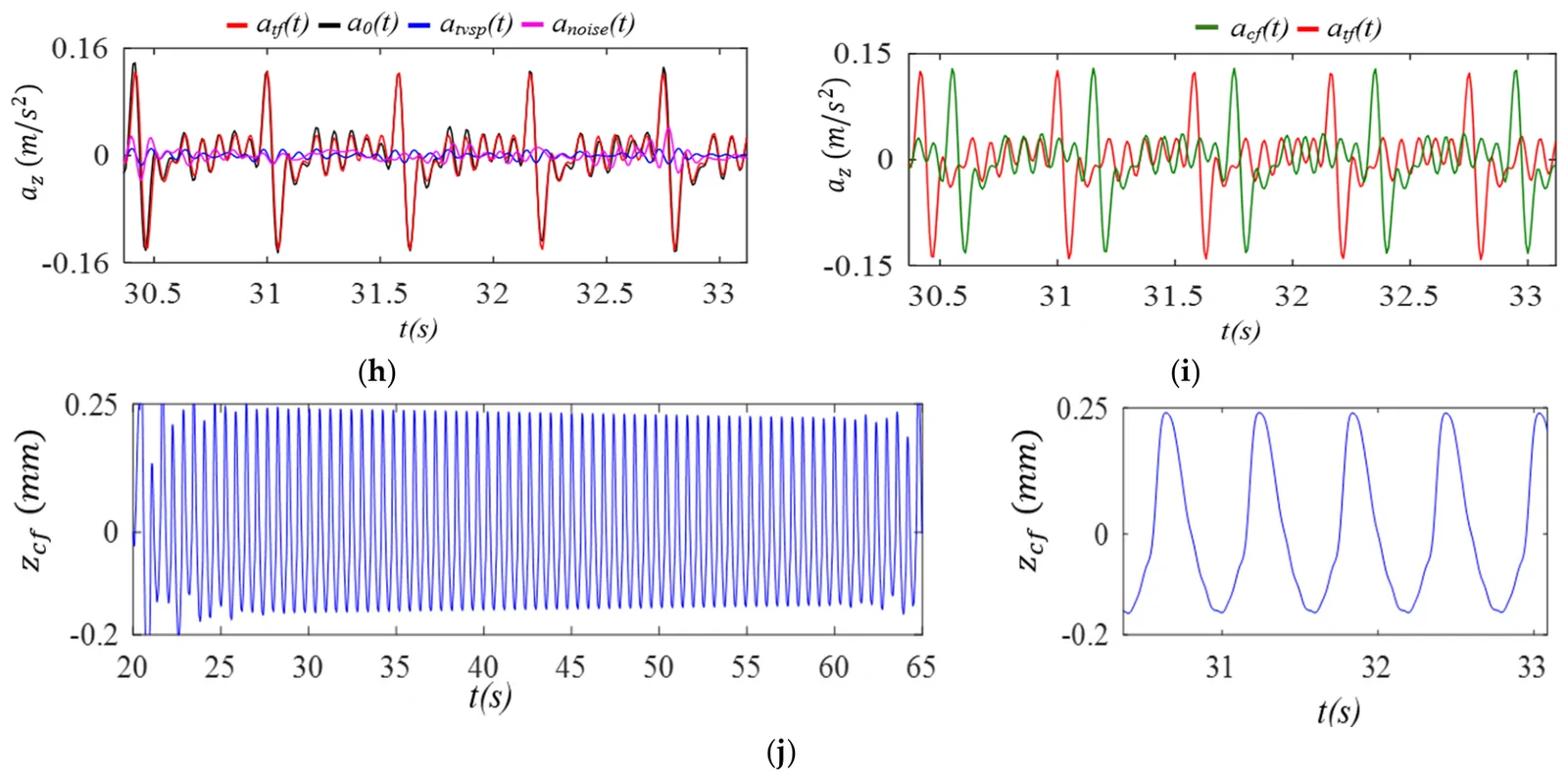
Measured acceleration signal of subject #8 using the analog ACC and its analyzed results: (**a**) originally measured acceleration signal *a_z_*(*t*) and the extracted baseline-drift acceleration *a_bd_*(*t*); (**b**) frequency spectra of different signal components in *a_z_*(*t*); (**c**) instant amplitude of *a_z_*(*t*); (**d**) instant frequency of *a_z_*(*t*); (**e**) instant initial phase of *a_z_*(*t*); (**f**) comparison of *a_z_*(*t*) with the extracted signal components; (**g**) comparison of *a_tf_*(*t*) with *a_cf_*(*t*); (**h**) an enlarged portion of the comparison of *a_z_*(*t*) with the extracted signal components; (**i**) an enlarged portion of the comparison of *a_tf_*(*t*) with *a_cf_*(*t*)*;* and (**j**) extracted *z_cf_*(*t*) using Equation (11).

**Figure 13:**
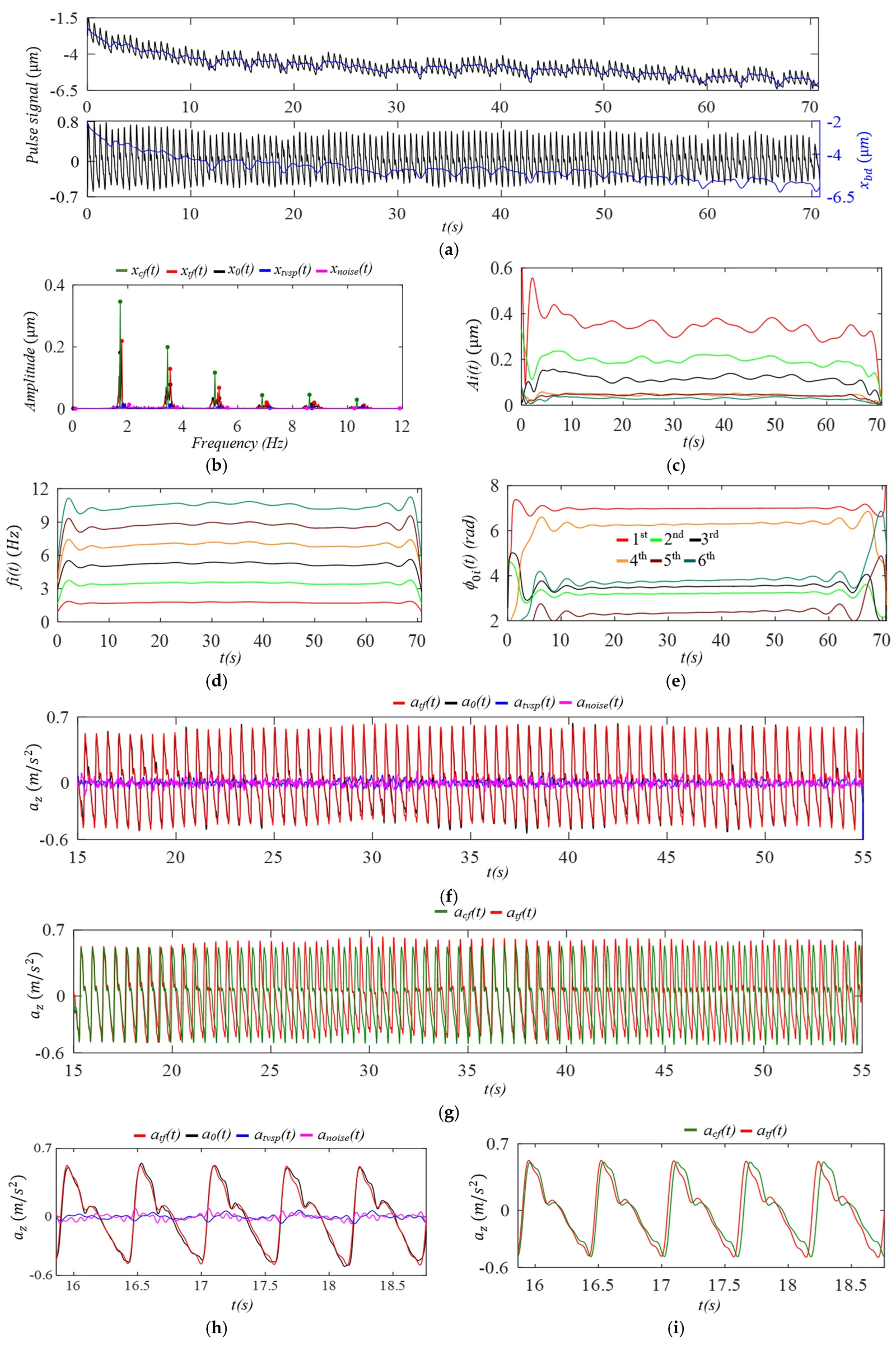
Measured pulse signal of subject #8 using the tactile sensor ACC and its analyzed results: (**a**) originally measured pulse signal *z_mea_*(*t*) = *z*_0_(*t*) + *z_bd_*(*t*) and the extracted baseline-drift acceleration *z_bd_*(*t*); (**b**) frequency spectra of different signal components in *z_mea_*(*t*); (**c**) instant amplitude of *z*_0_(*t*); (**d**) instant frequency of *z*_0_(*t*); (**e**) instant initial phase of *z*_0_(*t*); (**f**) comparison of *z*_0_(*t*) with *z_tvsp_*(*t*) and *z_noise_*(*t*); (**g**) comparison of *z_tf_*(*t*) with *z_cf_*(*t*); (**h**) an enlarged portion of the comparison of *z*_0_(*t*) with *z_tvsp_*(*t*) and *z_noise_*(*t*); (**i**) an enlarged portion of the comparison of *z_tf_*(*t*) with *z_cf_*(*t*).

**Figure 14:**
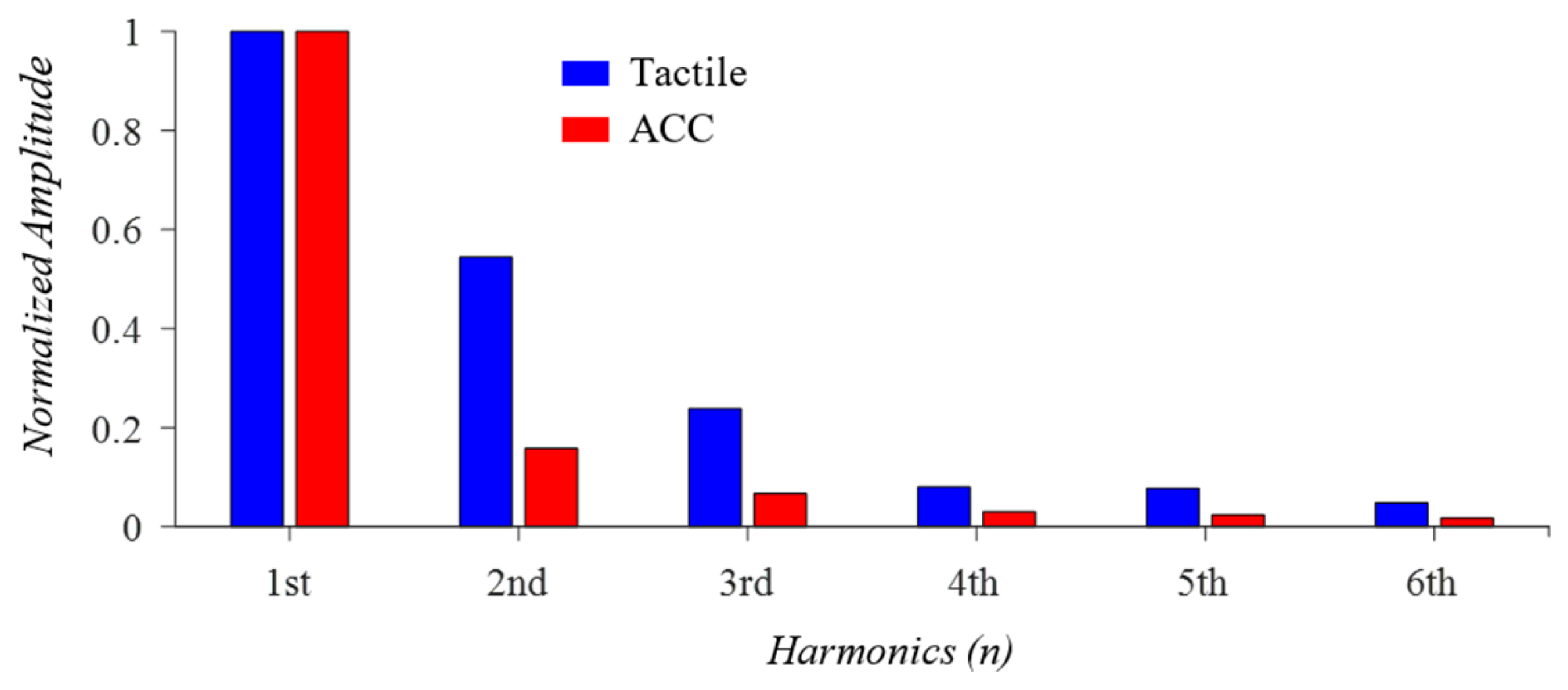
Comparison of normalized harmonic amplitude distribution relative to the first harmonic amplitude, *A_icf_*/*A*_1*cf*_, of Subject #8 between ACC and tactile sensor measurements.

**Figure 15:**
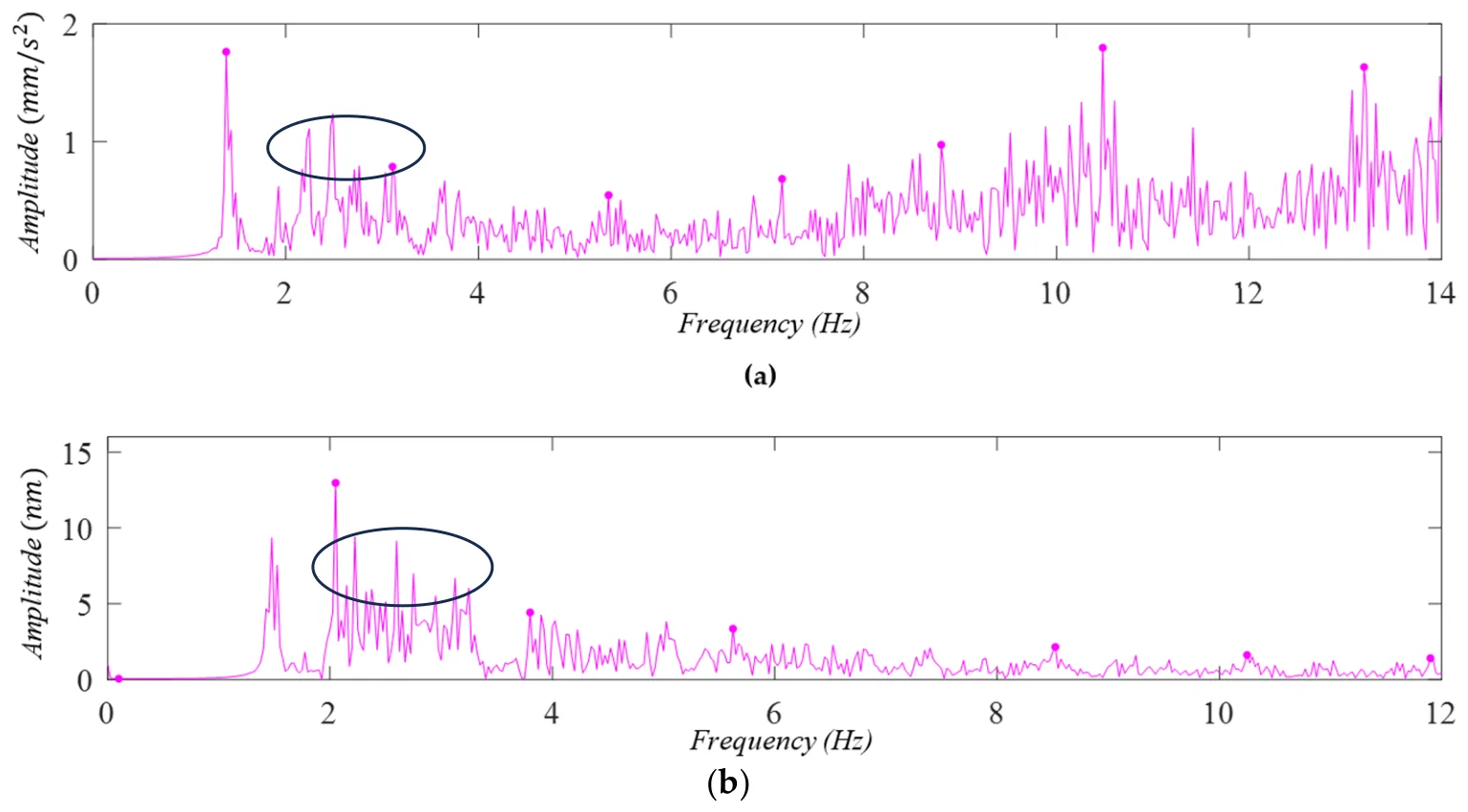
Comparison of the frequency spectra of sensor noise in the measured pulse signals of Subject #8: (**a**) ACC-based measurement; (**b**) tactile-based measurement (Note: Due to TVSP, the peak corresponding to the natural frequency of the TCS stack spans a frequency range).

**Figure 16:**
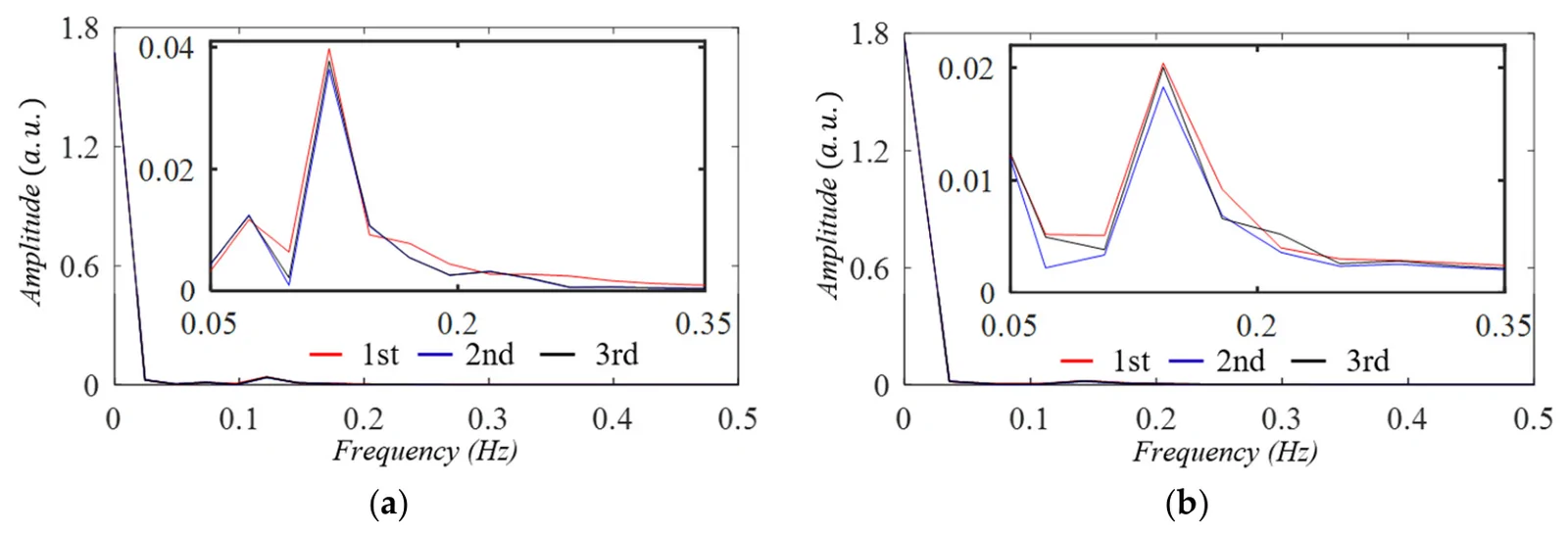
Comparison of the frequency spectra of the IFs of the first three harmonics of the measured pulse signals of Subject #8: (**a**) ACC-based measurement; (**b**) tactile-based measurement.

**Table 1:** Subject characteristics.

Subject ID	Age (Year)	Height (ft)	Weight (lb)	BMI	HR (bpm)	BP (mmHg)
#1 (digital and analog ACCs)	36	6.0	195	26.8	63	105/66
#2 (digital and analog ACCs)	20	5.8	149	21.4	84	110/60
#3 (digital and analog ACCs)	31	5.0	160	31.2	65	111/70
#4 (analog ACC)	29	5.8	125	18.2	107	110/70
#5 (analog ACC)	19	6.2	161	20.7	77	119/66
#6 (analog ACC and tactile)	40	5.1	120	22.7	--	--
#7 (analog ACC and tactile)	39	5.8	188	27	--	--
#8 (analog ACC and tactile)	33	5.3	170	29.1	--	--

**Table 2:** Summary of the extracted arterial indices from the measured acceleration signals by the analog AC of eight subjects: (**a**) beat-based method and (**b**) IF-based method.

(**a**)										
**Subjects**	**Age**	**BMI**	***b*** **/*a* Ratio**	**Δt_a−b_ (s)**	**HRV (s)** **(b-b)**	**HRV (s)** **(a-a)**	**HR (b-b) (bpm)**	**HRV (b-b) (bpm)**	**HR (a-a) (bpm)**	**HRV (a-a) (bpm)**
#1	36	26.8	−1.4309	0.0895	0.0746	0.0899	69.4792	5.3318	69.6074	6.2024
#2	20	21.4	−1.0727	0.0777	0.0551	0.0451	84.0528	6.058	83.9132	5.0476
#3	31	31.2	−1.1368	0.0667	0.0123	0.0116	64.0038	0.8483	63.9752	0.7915
#4	29	18.2	−1.5629	0.0952	0.0192	0.0225	102.3954	3.2536	102.4664	3.9175
#5	19	20.7	−1.0292	0.0634	0.0207	0.0203	77.268	2.0482	77.224	2.0089
#6	40	22.7	−0.4719	0.0514	0.0129	0.0127	77.7414	1.2921	77.7548	1.2765
#7	39	27	−0.6852	0.0541	0.0132	0.013	66.6312	0.9787	66.6144	0.9646
#8	33	29.1	−1.1583	0.0514	0.0119	0.0124	100.5102	2.0032	100.5313	2.075
(**b**)										
**Subjects**		**HR (bpm)**	**HRV (bpm)**		**RR (IF) (Breaths/min)**		**RR (IIP) (Breaths/min)**	**RM (IF) (a.u.)**	**RM (IIP) (a.u.)**	
#1		69.6203	5.0574		9.4665		15.3757	0.1640	0.0483	
#2		83.7189	4.2429		14.1845		20.4257	0.1081	0.0311	
#3		63.9192	2.6333		16.3002		17.9389	0.0642	0.0768	
#4		102.2063	3.2869		12.1334		15.2310	0.1017	0.0422	
#5		77.2446	2.4706		9.8621		14.5104	0.0523	0.0297	
#6		78.0268	1.5888		8.6448		13.3114	0.0341	0.0239	
#7		66.6823	1.3974		13.4806		15.3836	0.0418	0.0635	
#8		100.4671	1.9943		7.9327		12.0947	0.0769	0.0144	

**Table 3:** Harmonic amplitude distribution normalized to the first harmonic amplitude, *A_icf_*/*A*_1*cf*_, of the extracted APWs from the measured acceleration signals using the analog ACC.

	Subject	#1	#2	#3	#4	#5	#6	#7	#8
Harmonics									
1st		1	1	1	1	1	1	1	1
2nd		0.1486	0.1667	0.3134	0.2115	0.1768	0.1612	0.1756	0.1579
3rd		0.1096	0.0871	0.2031	0.1457	0.0932	0.0475	0.0697	0.0665
4th		0.0378	0.0230	0.0633	0.0315	0.0235	0.0258	0.0401	0.0301
5th		0.0290	0.0231	0.0483	0.0233	0.0242	0.0270	0.0285	0.0236
6th		0.0165	0.0117	0.0509	0.0056	0.0078	0.0169	0.0190	0.0172
7th		0.0098	0.0036	0.0190	0.0027	0.0047	0.0052	0.0081	0.0068

**Table 4:** Comparison of (**a**) the normalized harmonic amplitudes and (**b**) the absolute harmonic amplitude and standard deviation (SD) between the ACC-based and tactile-based measurement.

(**a**)												
	**#6**				**#7**				**#8**			
**Harmonics**	**Tactile**		**ACC**		**Tactile**		**ACC**		**Tactile**		**ACC**	
1st	1		1		1		1		1		1	
2nd	0.3244		0.1612		0.3651		0.1756		0.5441		0.1579	
3rd	0.1306		0.0475		0.2094		0.0697		0.2385		0.0665	
4th	0.0506		0.0258		0.0876		0.0401		0.0799		0.0301	
5th	0.0713		0.0270		0.0561		0.0285		0.0774		0.0236	
6th	0.0393		0.0169		0.0496		0.0190		0.0484		0.0172	
7th	0.0155		0.0052		0.0279		0.0081				0.0068	
(**b**)												
	**#6**				**#7**				**#8**			
**Harmonics**	**Tactile**	**SD**	**ACC**	**SD**	**Tactile**	**SD**	**ACC**	**SD**	**Tactile**	**SD**	**ACC**	**SD**
1st	0.2685	0.0360	180	38.747	1.097	0.133	300	50.175	0.2604	0.0210	130.0	17.450
2nd	0.0871	0.0146	29.6	4.737	0.401	0.061	52.4	4.845	0.1417	0.0130	20.2	2.361
3rd	0.0351	0.0088	8.73	1.279	0.230	0.039	20.8	1.966	0.0621	0.0102	8.5	1.087
4th	0.0136	0.0038	4.74	0.654	0.096	0.022	12	1.403	0.0208	0.0033	3.8	0.419
5th	0.0191	0.0055	4.96	0.565	0.062	0.019	8.52	0.531	0.0202	0.0025	3.0	0.329
6th	0.0106	0.0054	3.11	0.455	0.054	0.020	5.68	0.596	0.0126	0.0031	2.2	0.280
7th	0.0042	0.0036	0.962	0.328	0.031	0.023	2.41	0.465				

**Table 5:** Comparison of extracted IF-based HR and HRV, RR, and RM between ACC and tactile (TAC) sensor measurements.

Subject ID	#6		#7		#8	
Parameters	ACC	Tactile	ACC	Tactile	ACC	Tactile
HR (bpm)	78.0268	78.9287	66.6823	68.1628	100.4671	105.3623
HRV (bpm)	1.5888	1.6808	1.3974	1.8134	1.9943	1.8802
RR (IF) (bpm)	8.6448	6.9775	13.4806	13.3178	7.9327	8.2360
RR (IIP) (bpm)	13.3114	12.9600	15.3836	13.5326	12.0947	13.5752
RM(IF) (a.u.)	0.0373	0.0511	0.0418	0.0328	0.0769	0.0389
RM(IIP) (a.u.)	0.0239	0.0172	0.0635	0.0308	0.0144	0.0070

## Data Availability

The data that support the findings of this study are available upon reasonable request from the corresponding author. The data are not publicly available due to privacy or ethical restrictions.

## Supplementary Materials

The following supporting information can be downloaded at: https://www.mdpi.com/article/10.3390/s26216936/s1, Table S1. Acquisition parameters of the measurement systems associated with the digital and analog ACCs; Table S2. Comparison of signal-to-noise ratio (SNR) in measured acceleration signals at the CA between the digital ACC (LIS12DW) and the analog ACC (LIS344ALH); Table S3. Comparison of signal-to-noise ratio (SNR) in measured acceleration signals at the CA using the analog ACC (LIS344ALH) between subjects; Table S4. Harmonic power distribution (%) of the extracted APWs from the measured acceleration signals using the analog ACC (LIS344LAH); Figure S1. Output noise recorded from (a) the digital ACC and (b) the analog ACC under zero-input conditions. DC levels were shifted to display all three axes within a single plotting window; Figure S2. FFT spectra of the output noise recorded from (a) the digital ACC and (b) the analog ACC under zero-input conditions; Figure S3. Original CA pulse signals recorded from Subject #1: (a) digital ACC outputs and (b) analog ACC outputs. DC offsets in the digital ACC outputs were adjusted to display all three-axis outputs within a single plotting window. The rectangular box indicates the time window selected for analysis; Figure S4. Original CA pulse signals recorded from a subject whose recordings were excluded from subsequent analysis due to excessive sensor noise and substantial MA-related fluctuations in the z-axis: (a) digital ACC outputs and (b) analog ACC outputs. DC offsets in the digital ACC outputs were adjusted to display all three-axis outputs within a single plotting window; Figure S5. The original measured acceleration signals in three axes with BD removal of Subject #2; Figure S6. The original measured acceleration signals in three axes with BD removal of Subject #4; Figure S7. The extracted displacements with time-varying frequency in three axes from the measured acceleration signals using the analog ACC (LIS344ALH) (a) Subject #1; (b) Subject #3; and (c) Subject #5; Figure S8. Comparison of (a) *a_bd_*(*t*), *a_bd_re_*(*t*), and Δ*a_bd_*(*t*); and (b) *a*_0_(*t*), *a*_0*_re*_(*t*), and Δ*a*_0_(*t*) of Subject #6 from the analog ACC measurement; Figure S9. Measured acceleration signal of subject #6 using the analog ACC and its analyzed results (a) originally measured acceleration signal *a_z_*(*t*) and the extracted baseline-drift acceleration *a_bd_*(*t*); (b) frequency spectra of different signal components in *a_z_*(*t*); (c) instant amplitude of *a_z_*(*t*); (d) instant frequency of *a_z_*(*t*); (e) instant initial phase of *a_z_*(*t*); (f) comparison of *a_z_*(*t*) with the extracted signal components; (g) comparison of *a_tf_*(*t*) with *a_cf_*(*t*); and (h) extracted *z_cf_*(*t*) using Equation (11); Figure S10. Measured pulse signal of subject #6 using the tactile sensor ACC and its analyzed results (a) originally measured pulse signal *z_mea_*(*t*) = *z*_0_(*t*) + *z_bd_*(*t*) and the extracted baseline-drift acceleration *z_bd_*(*t*); (b) frequency spectra of different signal components in *z_mea_*(*t*); (c) instant amplitude of *z*_0_(*t*); (d) instant frequency of *z*_0_(*t*); (e) instant initial phase of *z*_0_(*t*); (f) comparison of *z*_0_(*t*) with *z_tvsp_*(*t*) and *z_noise_*(*t*); (g) comparison of *z_tf_*(*t*) with *z_cf_*(*t*); Figure S11. Comparison of normalized harmonic amplitude distribution relative to the first harmonic amplitude, *A_icf_*/*A*_1*cf*_, of Subject #6 between ACC and tactile sensor measurements; Figure S12. Comparison of the frequency spectra of sensor noise in the measured pulse signals of Subject #6 (a) ACC-based measurement (b) tactile-based measurement; Figure S13. Comparison of the frequency spectra of the IFs of the first three harmonics of the measured pulse signals of Subject #8 (a) ACC-based measurement (b) tactile-based measurement; Figure S14. Comparison of (a) *a_bd_*(*t*), *a_bd_re_*(*t*), and Δ*a_bd_*(*t*); and (b) *a*_0_(*t*), *a*_0*_re*_(*t*), and Δ*a*_0_(*t*) of Subject #8 from the analog ACC measurement; Figure S15. Measured acceleration signal of subject #7 using the analog ACC and its analyzed results (a) originally measured acceleration signal *a_z_*(*t*) and the extracted baseline-drift acceleration *a_bd_*(*t*); (b) frequency spectra of different signal components in *a_z_*(*t*); (c) instant amplitude of *a_z_*(*t*); (d) instant frequency of *a_z_*(*t*); (e) instant initial phase of *a_z_*(*t*); (f) comparison of *a_z_*(*t*) with the extracted signal components; (g) comparison of *a_tf_*(*t*) with *a_cf_*(*t*); and (h) extracted *z_cf_*(*t*) using Equation (11); Figure S16. Measured pulse signal of subject #8 using the tactile sensor ACC and its analyzed results (a) originally measured pulse signal *z_mea_*(*t*) = *z*_0_(*t*) + *z_bd_*(*t*) and the extracted baseline-drift acceleration *z_bd_*(*t*); (b) frequency spectra of different signal components in *z_mea_*(*t*); (c) instant amplitude of *z*_0_*(t)*; (d) instant frequency of *z*_0_(*t*); (e) instant initial phase of *z*_0_(*t*); (f) comparison of *z*_0_(*t*) with *z_tvsp_*(*t*) and *z_noise_*(*t*); (g) comparison of *z_tf_*(*t*) with *z_cf_*(*t*); Figure S17. Comparison of normalized harmonic amplitude distribution relative to the first harmonic amplitude, *A_icf_*/*A*_1*cf*_, of Subject #7 between ACC and tactile sensor measurements; Figure S18. Comparison of the frequency spectra of sensor noise in the measured pulse signals of Subject #7 (a) ACC-based measurement (b) tactile-based measurement; Figure S19. Comparison of the frequency spectra of the IFs of the first three harmonics of the measured pulse signals of Subject #7 (a) ACC-based measurement (b) tactile-based measurement.

## Abbreviations

The following abbreviations are used in this manuscript:

BD	Baseline drift
SDOF-TF	Single-degree-of-freedom time-frequency
TVSP	Time-varying system parameters
ACC	Accelerometer
MA	Motion artifacts
CA	Carotid artery
TFA	Time-frequency analysis
APW	Arterial pulse waveform
IA	Instantaneous amplitude
IF	Instantaneous frequency
IIP	Instantaneous initial phase
APW_a_	Arterial pulse acceleration waveforms
RR	Respiration rate
RM	Respiration modulation
TCS	Tissue-contact sensor
HR	Heart rate
