# Supplementary material for: Feasibility of Accelerometer-Based Arterial Pulse Measurement: Part II—Experimental Investigation
Source: Sensors (Basel). 2026 Nov 2;26(21):6936. doi: 10.3390/s26216936 (PMC13766535; doi:10.3390/s26216936)
Supplement: Supplementary file 1 [file sensors-26-06936-s001.zip › sensors-4602980-supplementary.pdf]

Supplementary Materials

Supplementary A

Details on the measurement systems associated with the digital and analog ACCs

Table S1. Acquisition parameters of the measurement systems associated with the digital and analog ACCs

Parameters	Analog ACC (LIS344ALH)	Digital ACC (LIS2DW12)
Measurement range	±2 g	±2 g
Data acquisition hardware and interface	NI USB-6002 DAQ	Arduino Nano 33 BLE; I²C
Acquisition sampling rate	1000 samples/s	100 samples/s
Sensor output type and output data rate	Continuous analog voltage	Digital; 200 Hz ODR
ADC resolution and input range	16-bit DAQ ADC; ±10 V input	14-bit; high-performance mode
Accelerometer sensitivity	0.66 V/g	0.244 mg/count
Front-end analog circuit gain	X: 46.5189<br>Y: 44.6202<br>Z: 46.8354	No external analog amplification
Acceleration resolution	X: 9.748×10^{-5}<br>Y: 1.0162×10^{-4}<br>Z: 9.682×10^{-5}<br>(m/s^2/count)	2.393×10^{-3}; all axes (m/s^2/count)
Accelerometer bandwidth	100 Hz	100 Hz
Front-end analog circuit filter	First-order RC low-pass; 48.2 Hz cutoff	None
Measured system noise	Not separately measured	Not separately measured

The following provides the derivation of the relationships used to convert the digital and analog ACC outputs into acceleration components.

Digital LIS2DW12

For the LIS2DW12, the Arduino library functions readFloatAccelX(), readFloatAccelY(), and readFloatAccelZ() provide the sensor outputs in mg (milligravity). The sensitivity is required to convert the raw digital readings; however, it was already incorporated in these library functions and therefore was not applied again. During acquisition, the outputs were scaled as

$$X_{out} = \frac{readFloatAccelX()}{10} + 80,$$

$$Y_{out} = \frac{readFloatAccelY() - 8}{10},$$

$$Z_{out} = \frac{readFloatAccelZ()}{10}$$

DC offsets are added to adjust the outputs so that they can be displayed in a single plotting window. Reversing these operations and using $1\,mg = 9.80665 \times 10^{-3}\,m/s^2$, the acceleration components were calculated as

$$a_X = -10(X_{out} - 80) \times 9.80665 \times 10^{-3}\,m/s^2$$
$$a_Y = (10Y_{out} + 8) \times 9.80665 \times 10^{-3}\,m/s^2$$
$$a_Z = 10Z_{out} \times 9.80665 \times 10^{-3}\,m/s^2$$

Analog LIS344ALH

For the LIS344ALH, the analog voltage outputs were amplified by the front-end analog circuit before feeding into the DAQ board. The amplification factors were 46.5189, 44.6202 , and 46.8354 for the X, Y, and Z-axis channels, respectively, and the sensor sensitivity was $0.66\,V/g$ (Volt/gravity). The acceleration components were calculated as

$$a_X = -(V_X/(46.5189 \times 0.66)) \times 9.80665\,m/s^2$$
$$a_Y = (V_Y/(44.6202 \times 0.66)) \times 9.80665\,m/s^2$$
$$a_Z = (V_Z/(46.8354 \times 0.66)) \times 9.80665\,m/s^2$$

Here, V_X, V_Y, and V_Z denote the amplified LIS344ALH voltage outputs. For both accelerometers, the X-axis sign was reversed because, with the sensor orientation used on the neck, the positive X-axis was directed toward the head and opposite to the selected gravitational reference direction.

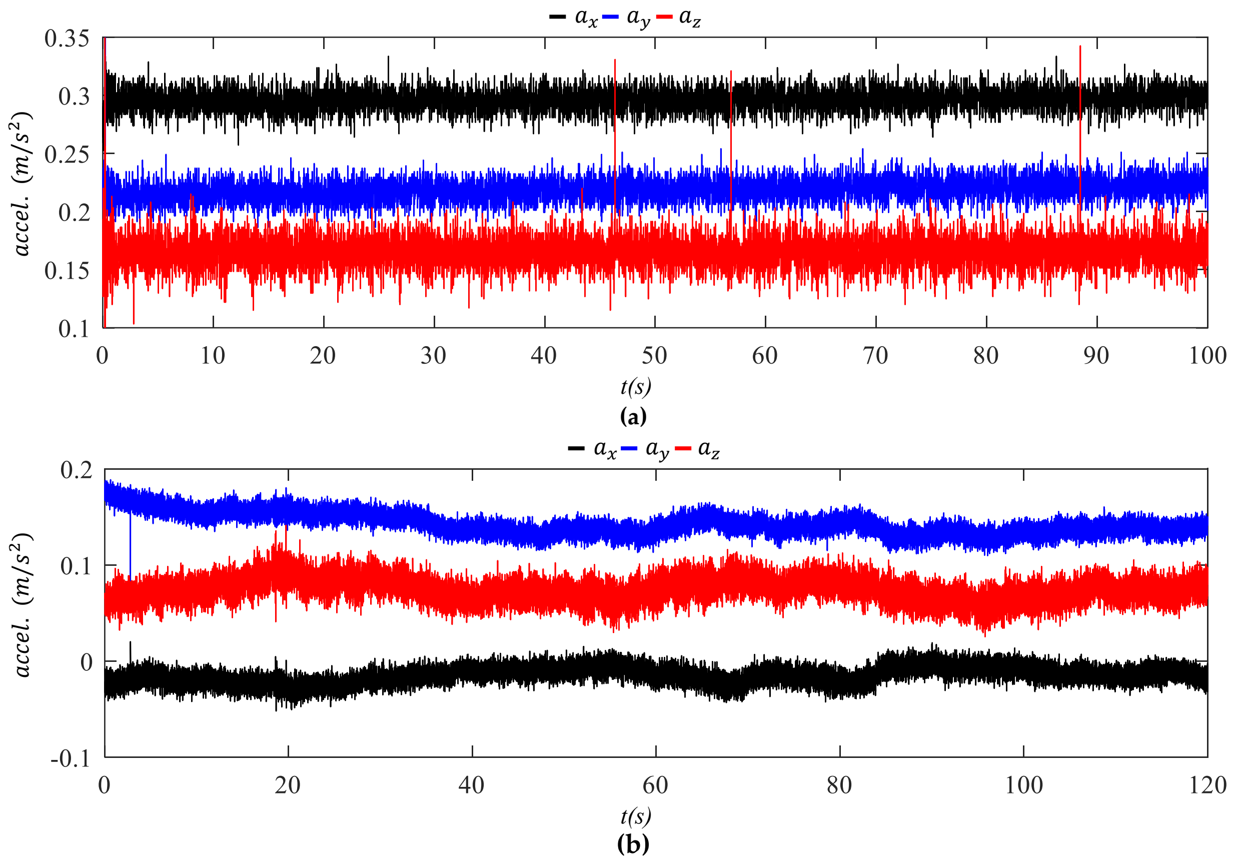

Figure S1. Output noise recorded from (a) the digital ACC and (b) the analog ACC under zero-input conditions. DC levels were shifted to display all three axes within a single plotting window.

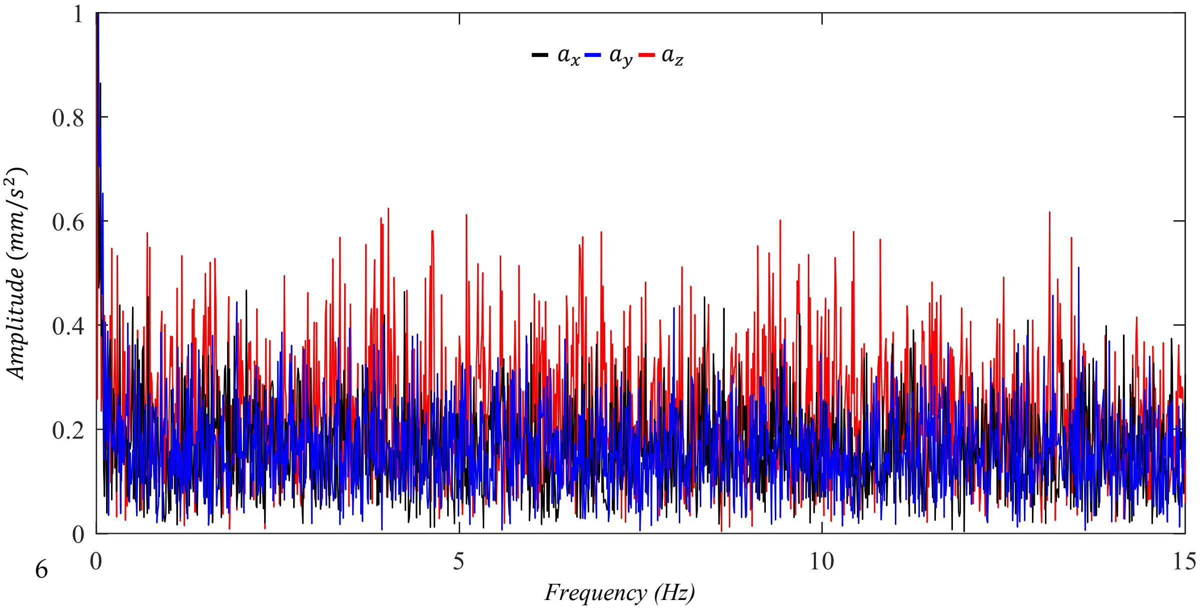

(a)

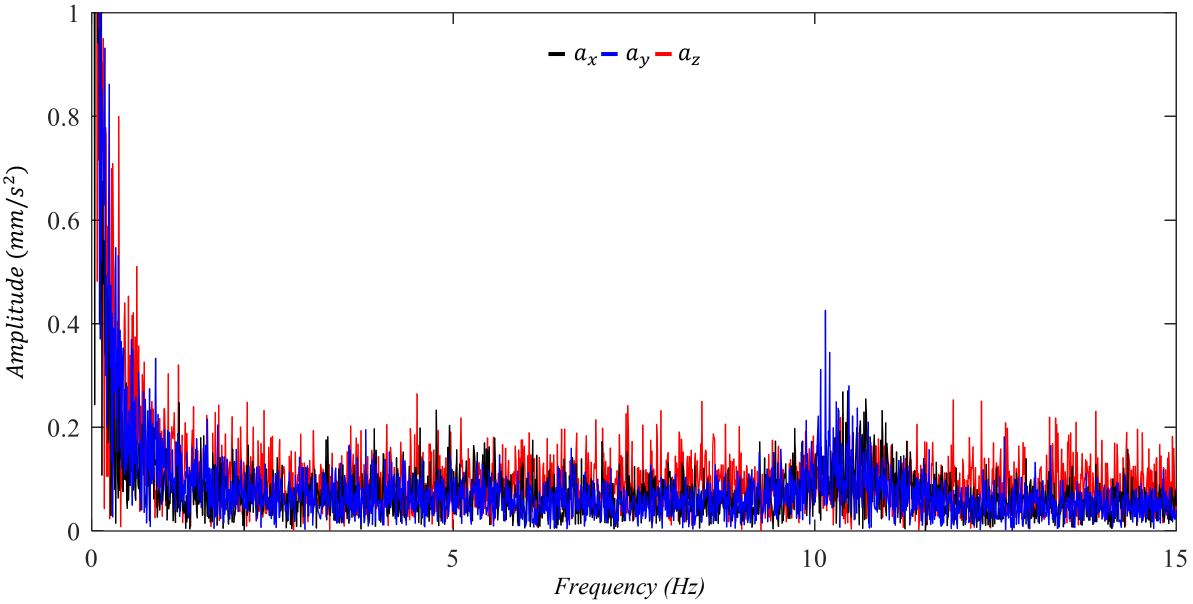

(b)

Figure S2. FFT spectra of the output noise recorded from (a) the digital ACC and (b) the analog ACC under zero-input conditions.

Supplementary B

Examples of pulse recordings that met and did not meet the signal-quality criteria

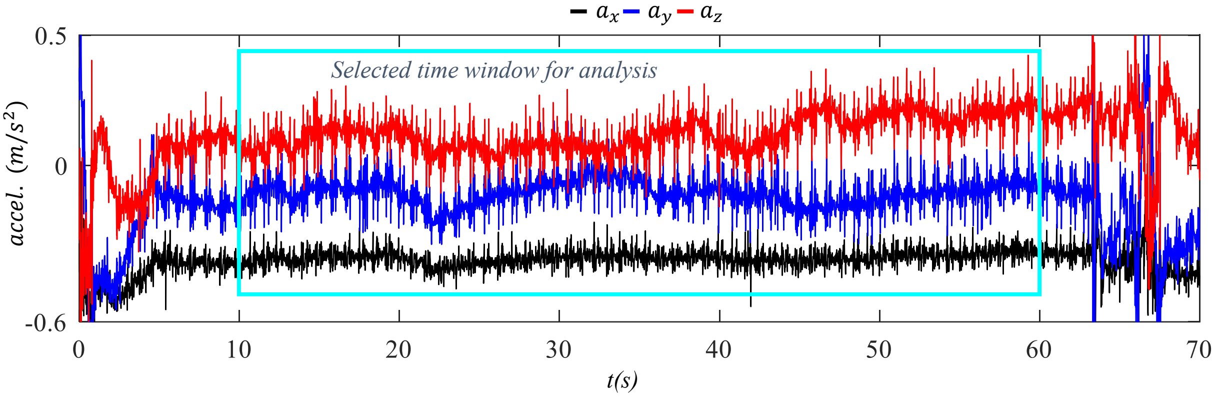

(a)

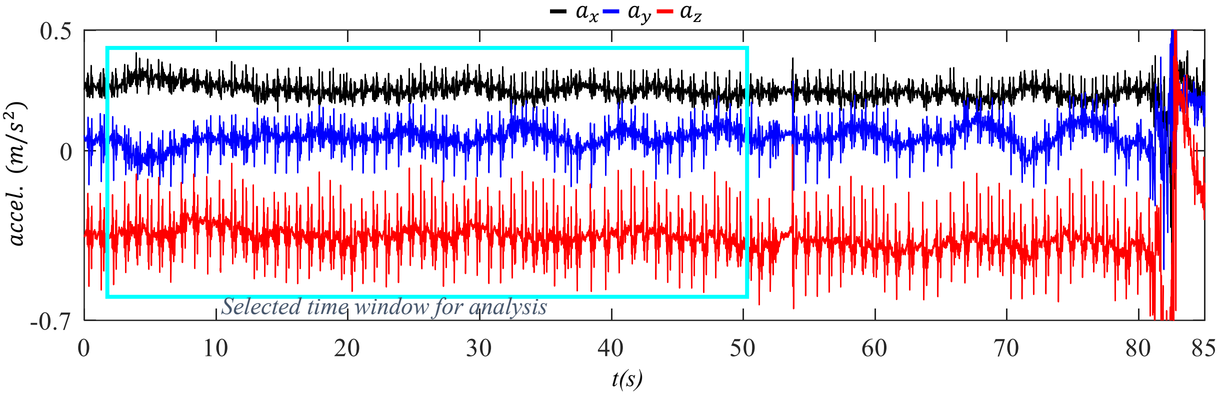

(b)

Figure S3. Original CA pulse signals recorded from Subject #1: (a) digital ACC outputs and (b) analog ACC outputs. DC offsets in the digital ACC outputs were adjusted to display all three-axis outputs within a single plotting window. The rectangular box indicates the time window selected for analysis.

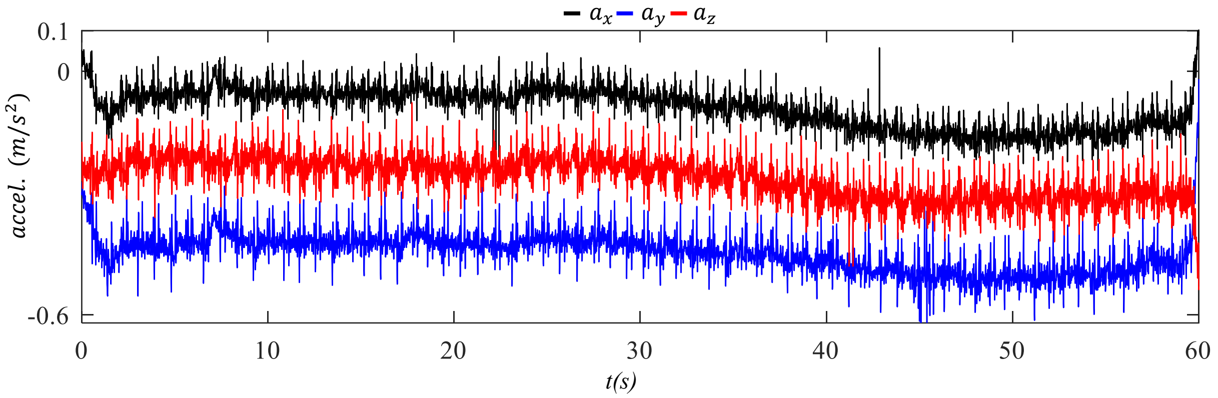

(a)

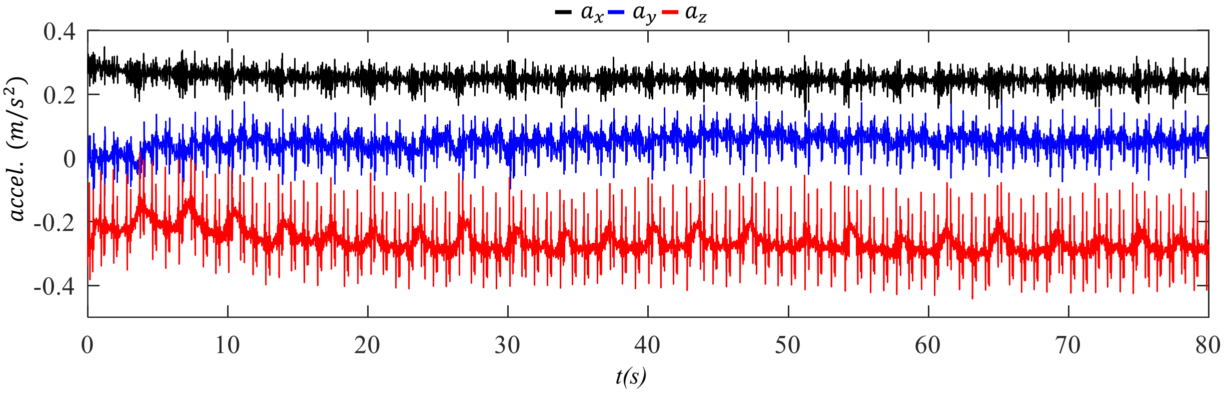

(b)

Figure S4. Original CA pulse signals recorded from a subject whose recordings were excluded from subsequent analysis due to excessive sensor noise and substantial MA-related fluctuations in the z-axis: (a) digital ACC outputs and (b) analog ACC outputs. DC offsets in the digital ACC outputs were adjusted to display all three-axis outputs within a single plotting window.

Supplementary C

Comparison of signal-to-noise ratio of measured acceleration signals using two accelerometers

As shown in Table S1, compared with the digital ACC, the analog ACC provides a higher ratio of $a_{tf}(t)$ to $a_{tvsp}(t) + a_{noise}(t)$, which determines the quality of the measured pulse signal for both Subject #2 and Subject #3. Only Subject #1 exhibits a relatively low SNR in the analog ACC measurement; nevertheless, it remains higher than that obtained with the digital ACC.

Table S2. Comparison of signal-to-noise ratio (SNR) in measured acceleration signals at the CA between the digital ACC (LIS12DW) and the analog ACC (LIS344ALH)

Parameters	#1		#2		#3	
	analog	digital	analog	digital	analog	digital
SNR (a_{HVD}/a_{noise})	4.3605	4.3346	8.2778	5.4201	8.446	7.8813
SNR (a_{tf}/a_{tvsp})	9.462	6.8602	10.3811	7.2573	11.3653	10.658
SNR ($a_{tf}/(a_{tvsp} + a_{noise})$)	2.914	1.8246	6.0103	2.7597	6.012	5.9153

As shown in Table S2, except Subject #1 and #7, the ratio of $a_{tf}(t)$ to $a_{tvsp}(t) + a_{noise}(t)$ is relatively high, with Subject #8 achieving the highest ratio.

Table S3. Comparison of signal-to-noise ratio (SNR) in measured acceleration signals at the CA using the analog ACC (LIS344ALH) between subjects

**Parameters**	**#1**	**#2**	**#3**	**#4**	**#5**	**#6**	**#7**	**#8**
SNR (a_{HVD}/a_{noise})	4.3605	8.2778	8.446	9.7989	8.7151	7.7273	7.3051	9.239
SNR (a_{tf}/a_{tvsp})	9.462	10.3811	11.3653	10.2595	10.9219	10.0504	5.9905	11.5762
SNR ($a_{tf}/(a_{tvsp} + a_{noise})$)	2.914	6.0103	6.012	6.8158	6.6383	5.5998	2.833	7.1473

Supplementary D

Comparison of measured radial, axial, and lateral accelerations after BD removal using the analog ACC (LIS344ALH)

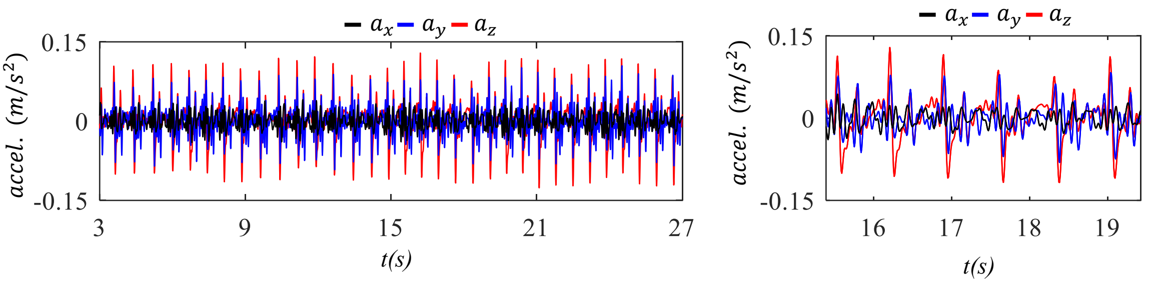

Figure S5. The original measured acceleration signals in three axes with BD removal of Subject #2

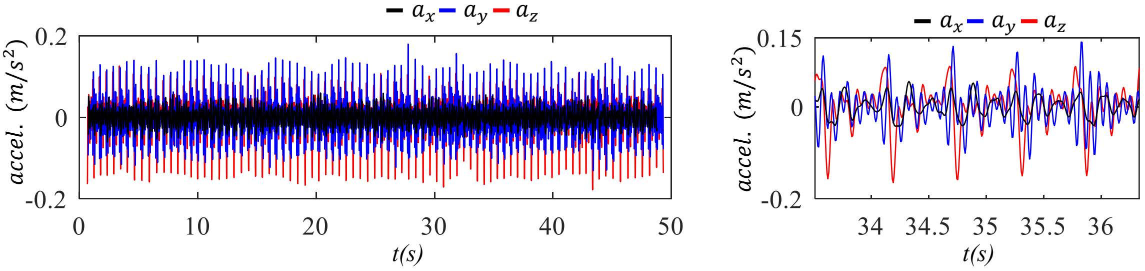

Figure S6. The original measured acceleration signals in three axes with BD removal of Subject #4

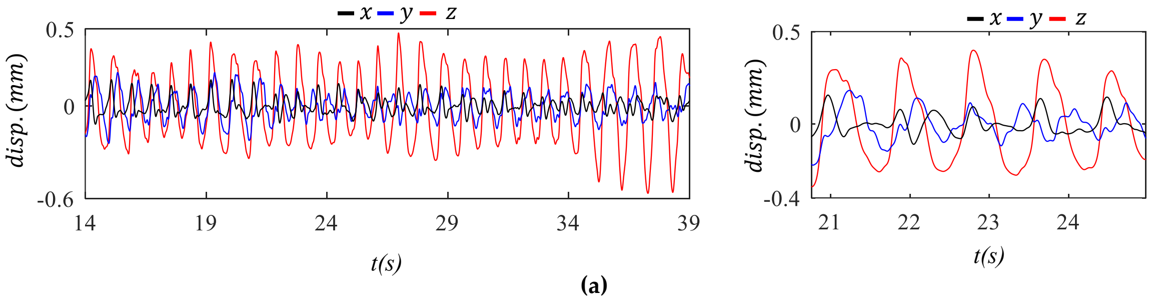

(a)

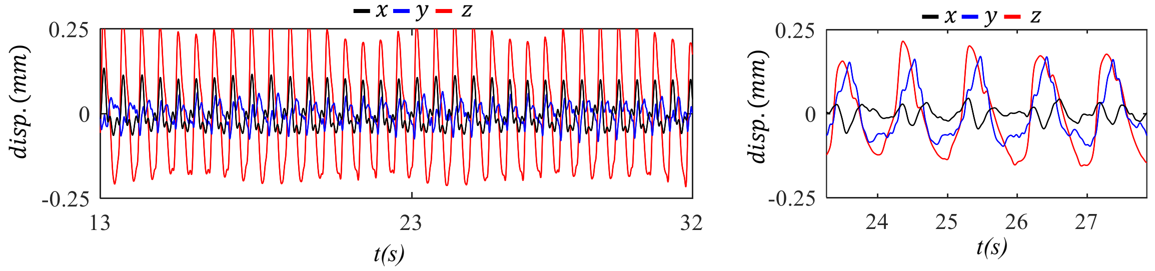

(b)

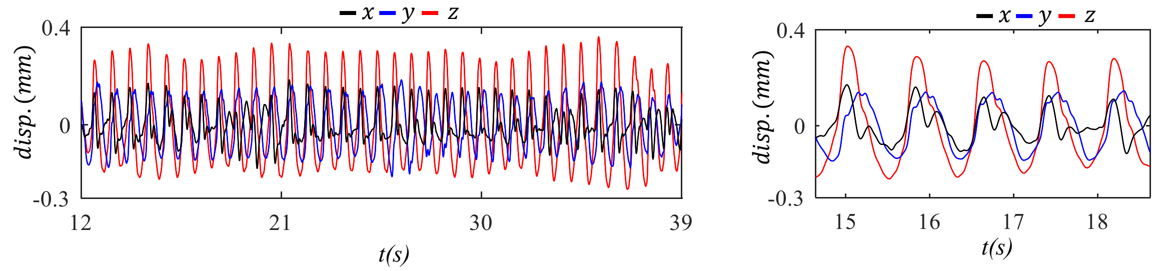

(c)

Figure S7. The extracted displacements with time-varying frequency in three axes from the measured acceleration signals using the analog ACC (LIS344ALH) (a) Subject #1; (b) Subject #3; and (c) Subject #5.

Supplementary E

Harmonic power distribution (%) of the extracted APWs from the measured acceleration signals

Table S4. Harmonic power distribution (%) of the extracted APWs from the measured acceleration signals using the analog ACC (LIS344LAH)

Harmonic	#1	#2	#3	#4	#5	#6	#7	#8
1st	96.4591	96.4712	***87.0508***	93.6748	96.0456	97.0918	96.2905	96.9771
2nd	2.1292	2.6802	***8.5502***	4.1903	3.0033	2.5228	2.9680	2.4191
3rd	1.1577	0.7314	***3.5901***	1.9873	0.8342	0.2194	0.4673	0.4290
4th	0.1376	0.0512	***0.3488***	0.0930	0.0528	0.0647	0.1548	0.0879
5th	0.0809	0.0517	***0.2032***	0.0510	0.0561	0.0708	0.0783	0.0539
6th	0.0263	0.0131	***0.2256***	0.0029	0.0058	0.0279	0.0348	0.0285
7th	0.0092	0.0012	***0.0313***	0.0007	0.0021	0.0027	0.0062	0.0045

Supplementary F

Comparison of the measured pulse signals between the analog ACC (LIS344ALH) and the tactile sensor of Subject #6

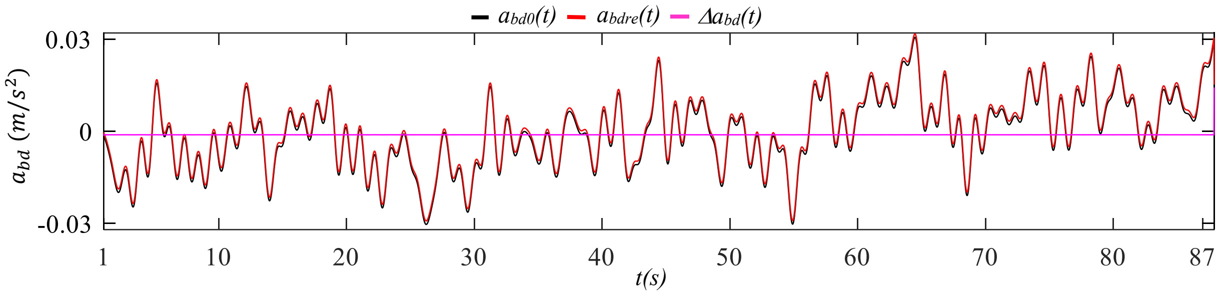

(a)

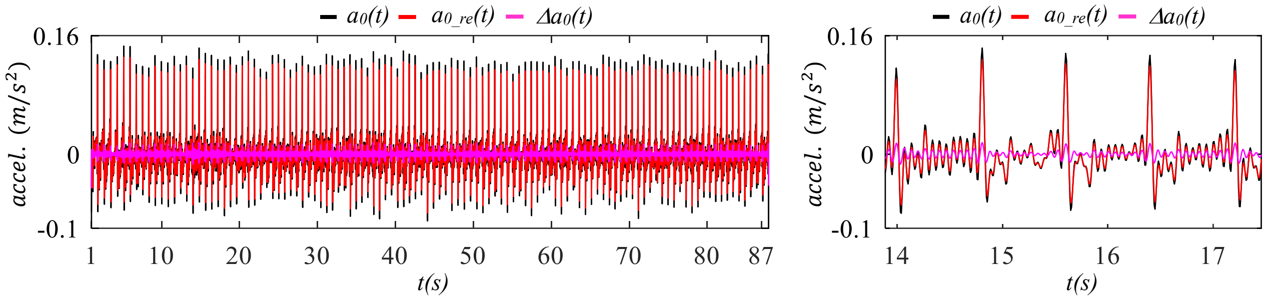

(b)

Figure S8. Comparison of **(a)** $a_{bd}(t)$, $a_{bd\_re}(t)$, and $\Delta a_{bd}(t)$; and **(b)** $a_0(t)$, $a_{0\_re}(t)$, and $\Delta a_0(t)$ of Subject #6 from the analog ACC measurement.

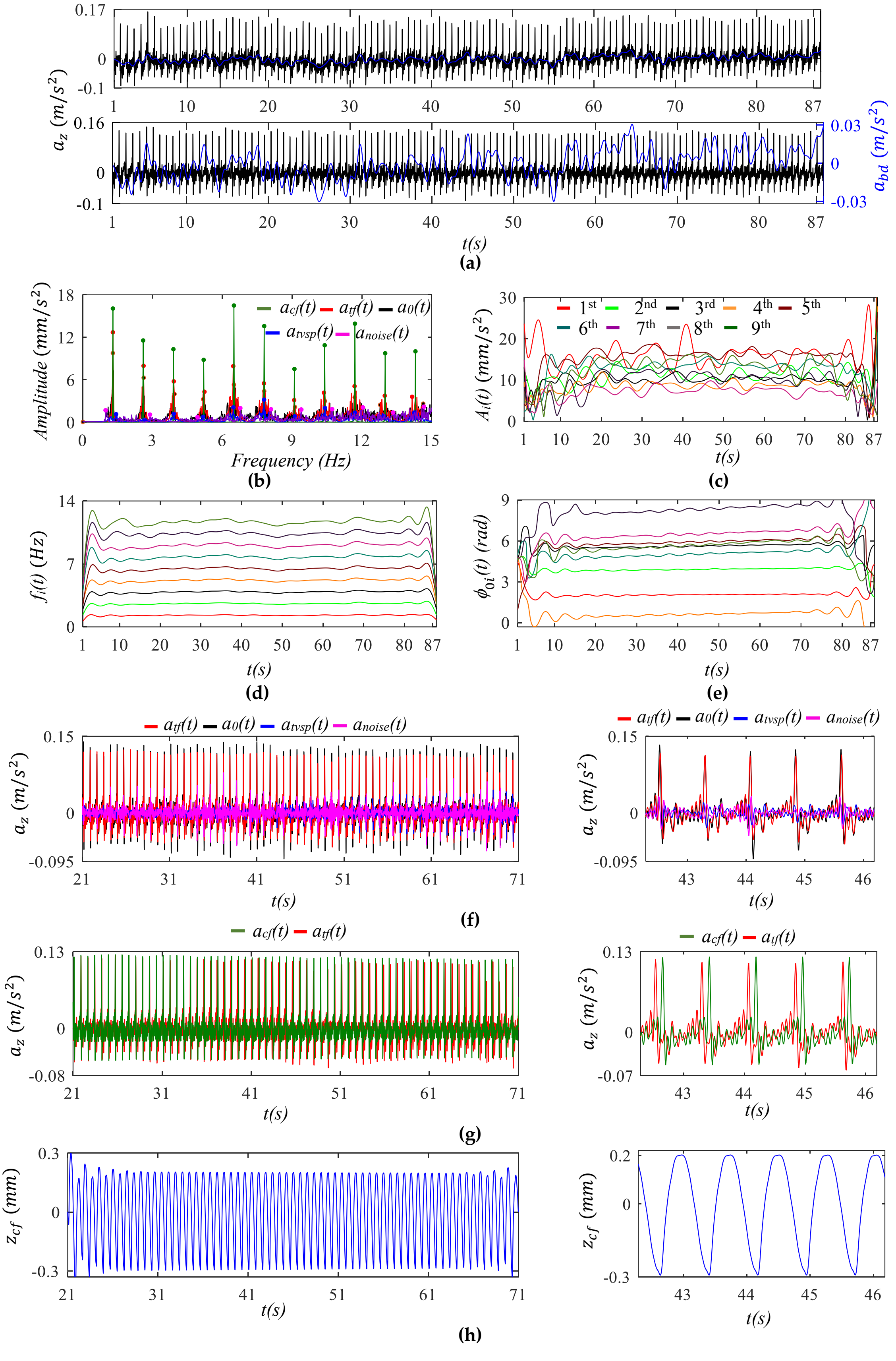

Figure S9. Measured acceleration signal of subject #6 using the analog ACC and its analyzed results **(a)** originally measured acceleration signal $a_z(t)$ and the extracted baseline-drift acceleration $a_{bd}(t)$; **(b)** frequency spectra of different signal components in $a_z(t)$; **(c)** instant amplitude of $a_z(t)$; **(d)** instant frequency of $a_z(t)$; **(e)** instant initial phase of $a_z(t)$; **(f)** comparison of $a_z(t)$ with the extracted signal components; **(g)** comparison of $a_{tf}(t)$ with $a_{cf}(t)$; and **(h)** extracted $z_{cf}(t)$ using Equation (11)

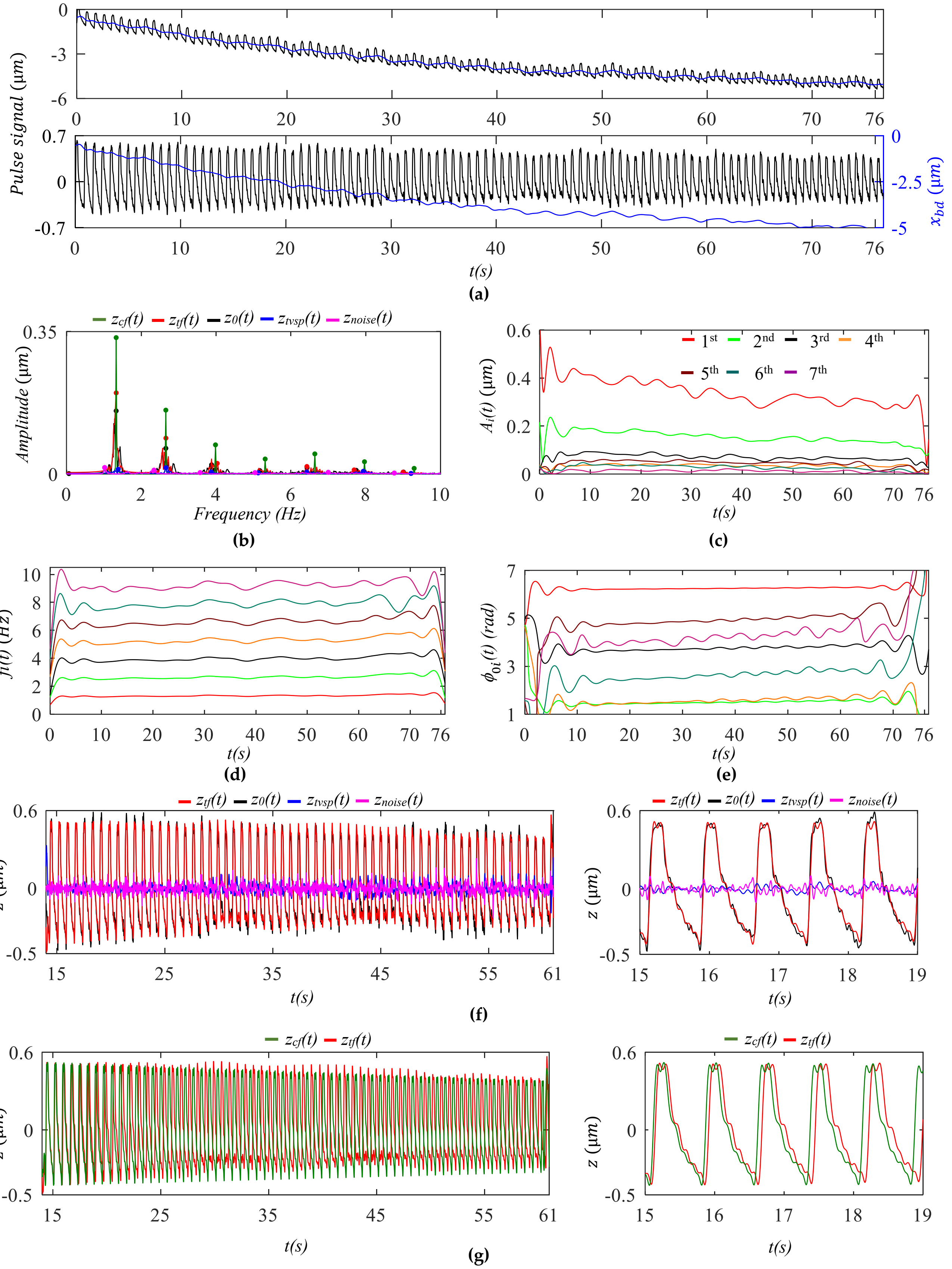

Figure S10. Measured pulse signal of subject #6 using the tactile sensor ACC and its analyzed results **(a)** originally measured pulse signal $z_{mea}(t)= z_0(t)+z_{bd}(t)$ and the extracted baseline-drift acceleration $z_{bd}(t)$; **(b)** frequency spectra of different signal components in $z_{mea}(t)$; **(c)** instant amplitude of $z_0(t)$; **(d)** instant frequency of $z_0(t)$; **(e)** instant initial phase of $z_0(t)$; **(f)** comparison of $z_0(t)$ with $z_{tvsp}(t)$ and $z_{noise}(t)$; **(g)** comparison of $z_{tf}(t)$ with $z_{cf}(t)$

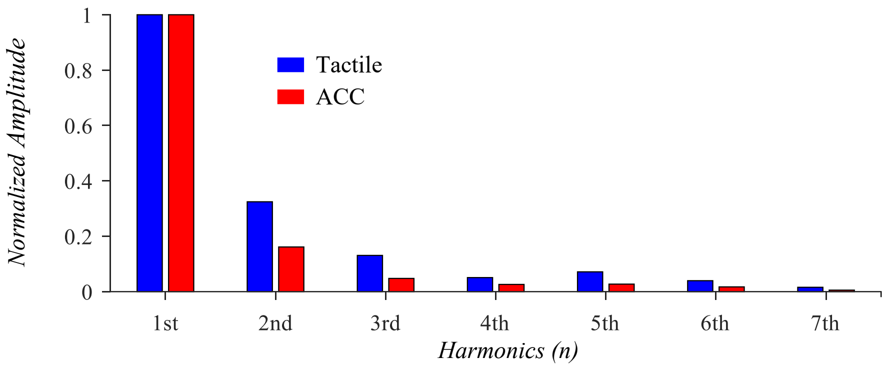

Figure S11. Comparison of normalized harmonic amplitude distribution relative to the first harmonic amplitude, A_{icf}/A_{1cf}, of Subject #6 between ACC and tactile sensor measurements

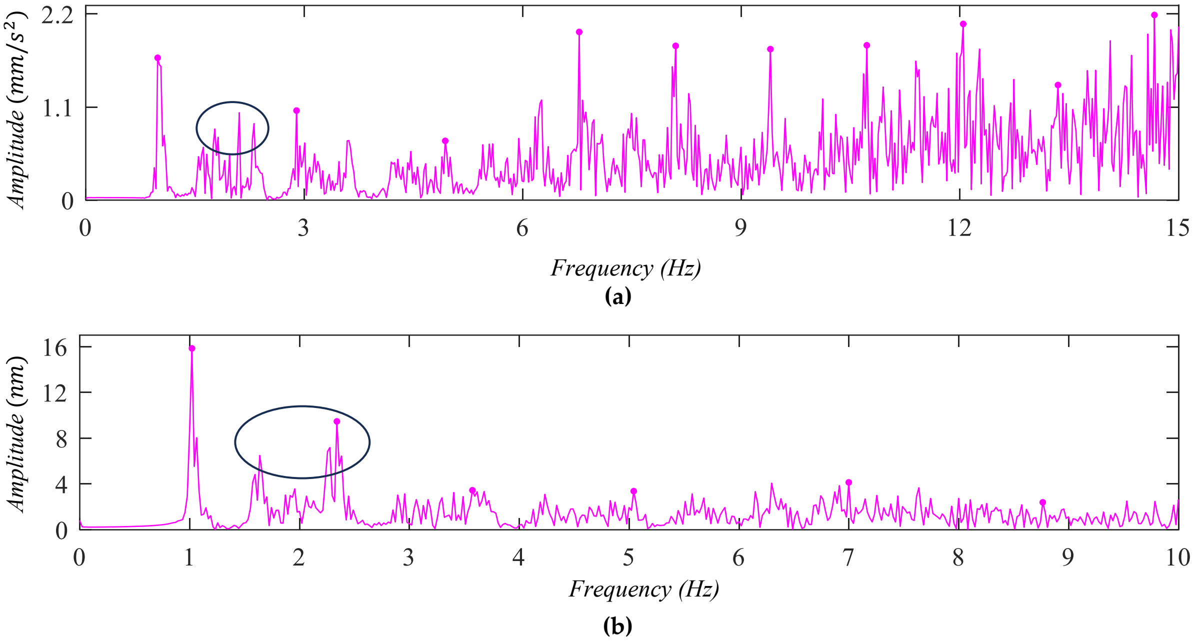

Figure S12. Comparison of the frequency spectra of sensor noise in the measured pulse signals of Subject #6 **(a)** ACC-based measurement **(b)** tactile-based measurement (Note: Due to TVSP, the peak corresponding to the natural frequency of the TCS stack spans a frequency range.)

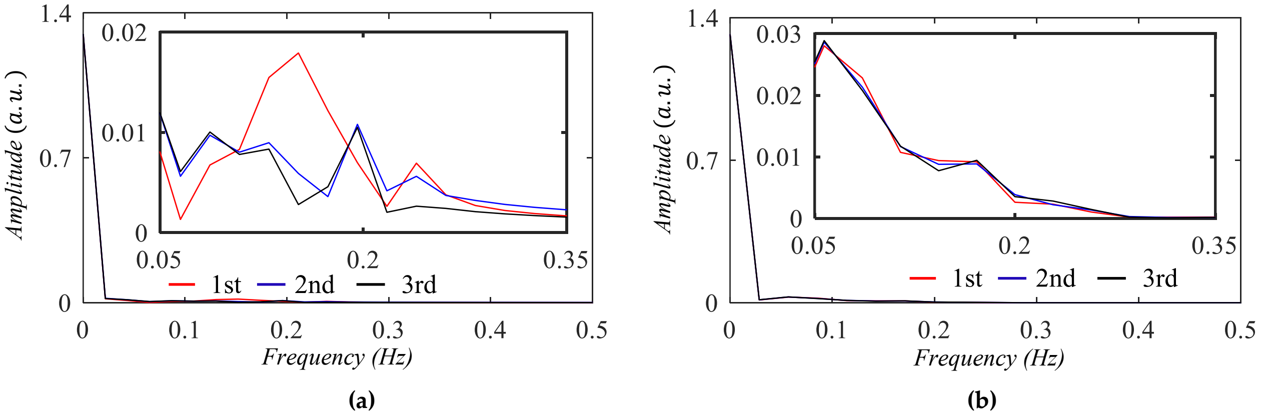

Figure S13. Comparison of the frequency spectra of the IFs of the first three harmonics of the measured pulse signals of Subject #8 **(a)** ACC-based measurement **(b)** tactile-based measurement

Supplementary G

Comparison of the measured pulse signals between the analog ACC (LIS344ALH) and the tactile sensor of Subject #7

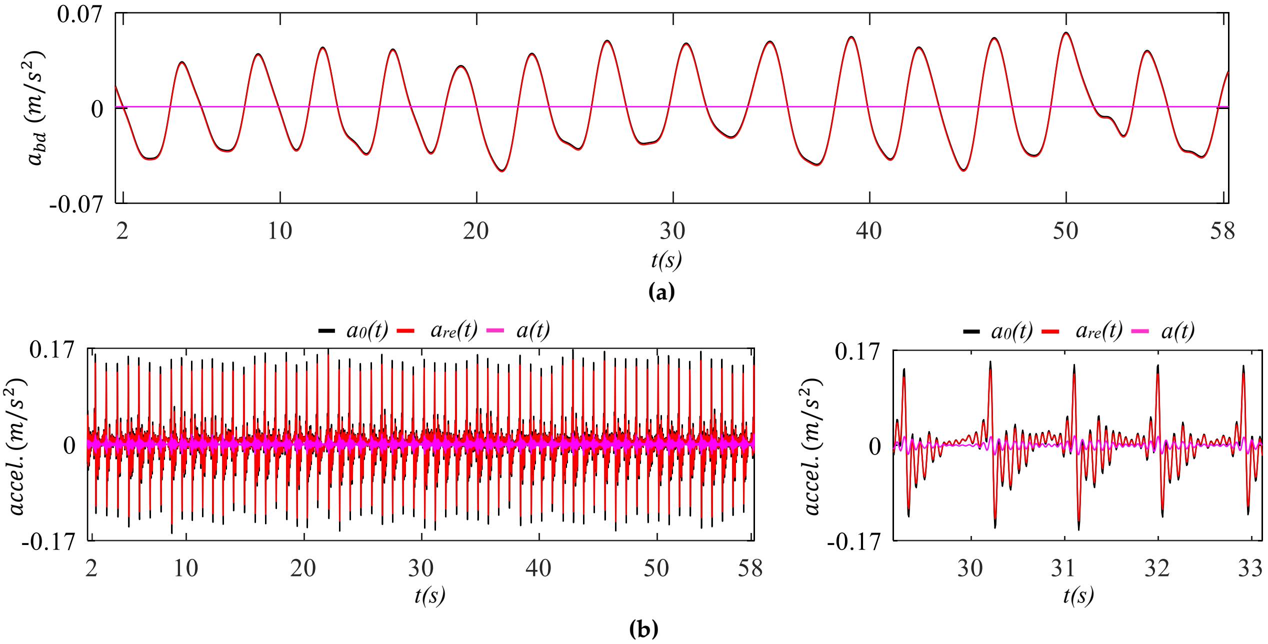

Figure S14. Comparison of **(a)** $a_{bd}(t)$, $a_{bd\_re}(t)$, and $\Delta a_{bd}(t)$; and **(b)** $a_0(t)$, $a_{0\_re}(t)$, and $\Delta a_0(t)$ of Subject #8 from the analog ACC measurement.

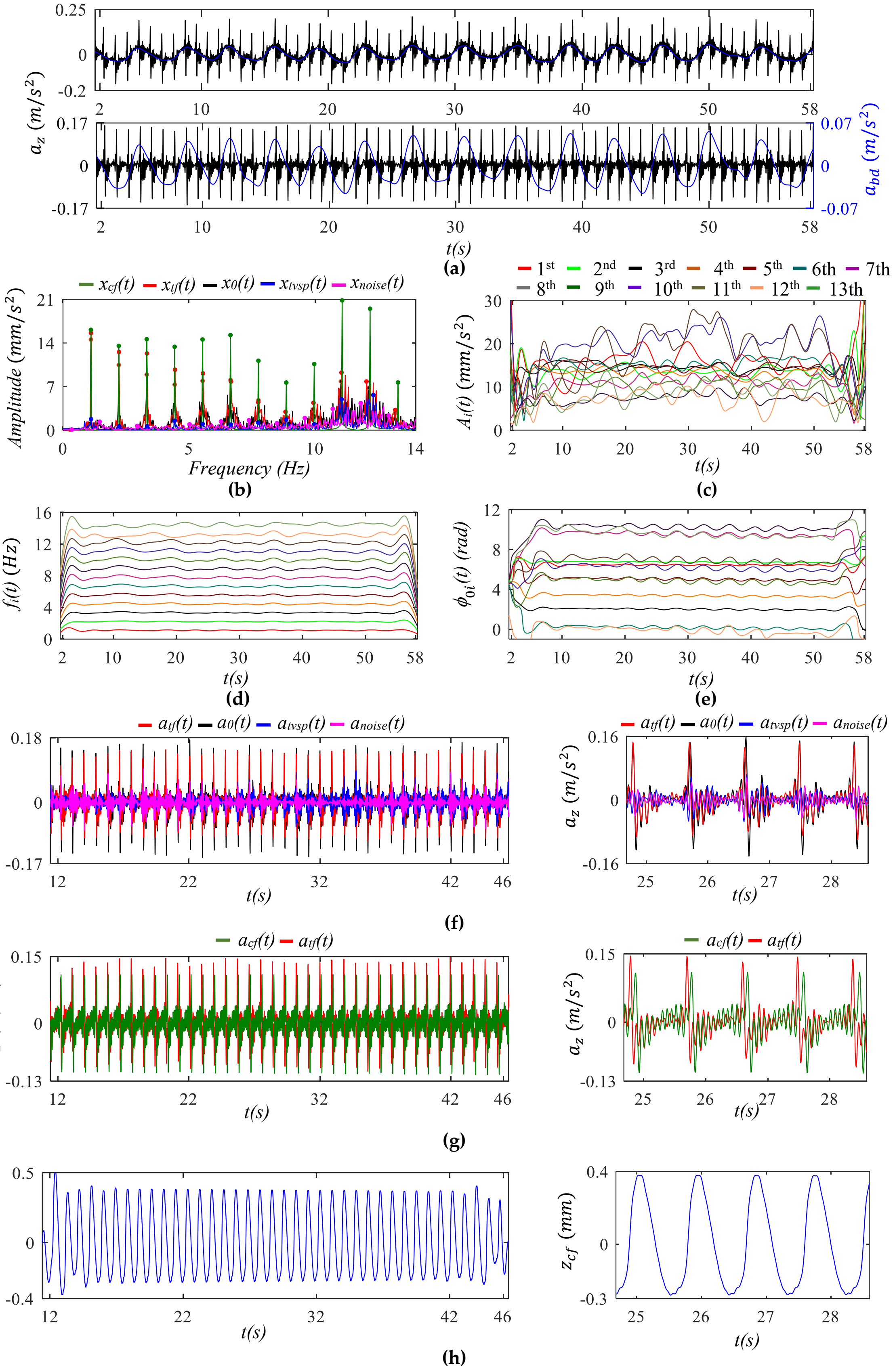

Figure S15. Measured acceleration signal of subject #7 using the analog ACC and its analyzed results **(a)** originally measured acceleration signal $a_z(t)$ and the extracted baseline-drift acceleration $a_{bd}(t)$; **(b)** frequency spectra of different signal components in $a_z(t)$; **(c)** instant amplitude of $a_z(t)$; **(d)** instant frequency of $a_z(t)$; **(e)** instant initial phase of $a_z(t)$; **(f)** comparison of $a_z(t)$ with the extracted signal components; **(g)** comparison of $a_{tf}(t)$ with $a_{cf}(t)$; and **(h)** extracted $z_{cf}(t)$ using Equation (11)

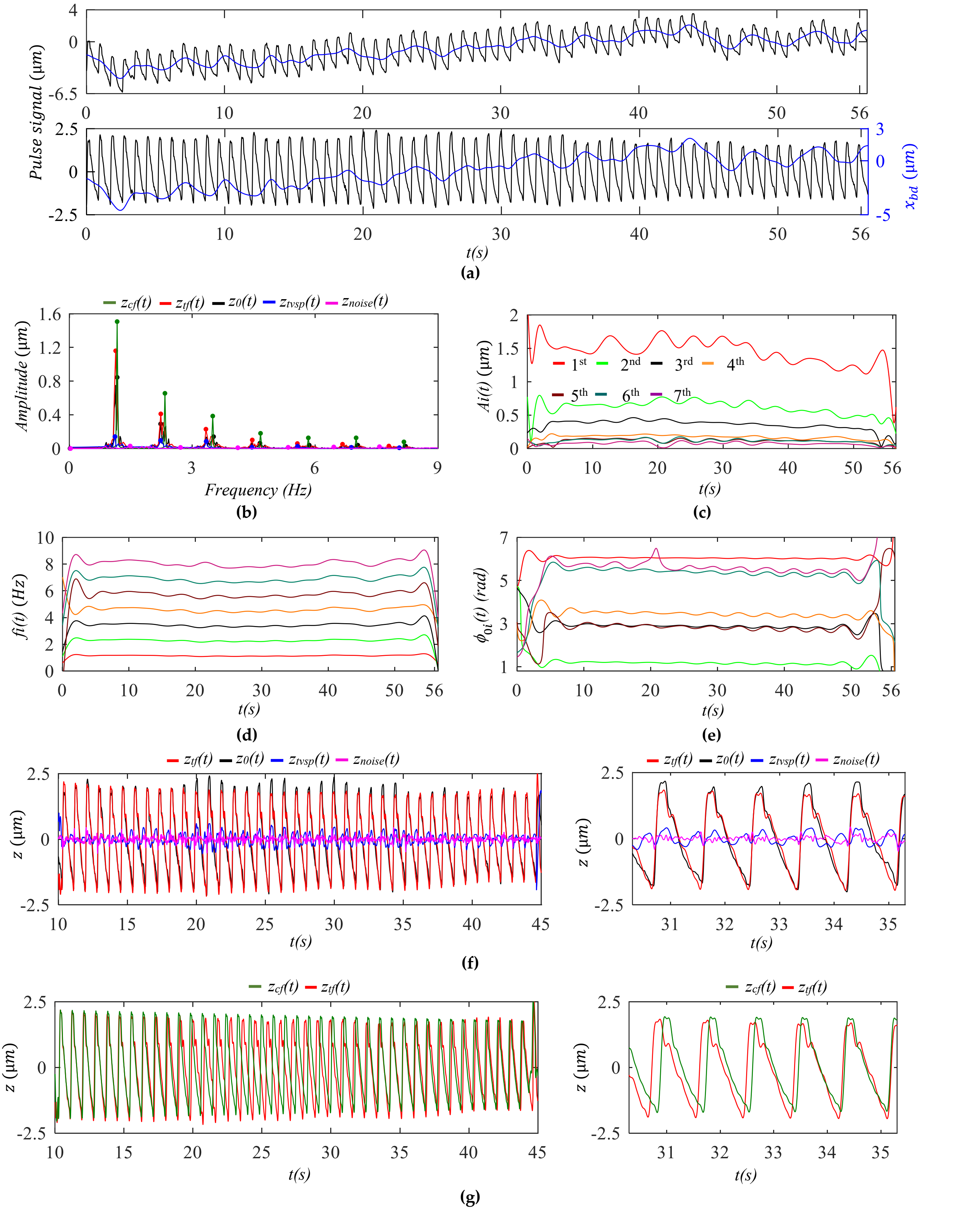

Figure S16. Measured pulse signal of subject #8 using the tactile sensor ACC and its analyzed results **(a)** originally measured pulse signal $z_{mea}(t)= z_0(t)+z_{bd}(t)$ and the extracted baseline-drift acceleration $z_{bd}(t)$; **(b)** frequency spectra of different signal components in $z_{mea}(t)$; **(c)** instant amplitude of $z_0(t)$; **(d)** instant frequency of $z_0(t)$; **(e)** instant initial phase of $z_0(t)$; **(f)** comparison of $z_0(t)$ with $z_{tvsp}(t)$ and $z_{noise}(t)$; **(g)** comparison of $z_{tf}(t)$ with $z_{cf}(t)$

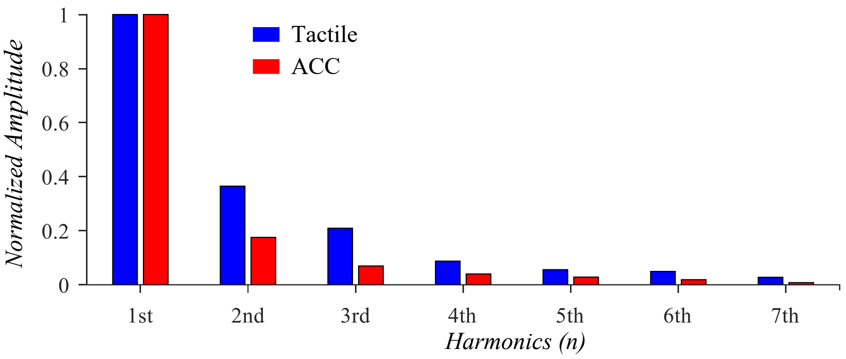

Figure S17. Comparison of normalized harmonic amplitude distribution relative to the first harmonic amplitude, A_{icf}/A_{1cf}, of Subject #7 between ACC and tactile sensor measurements

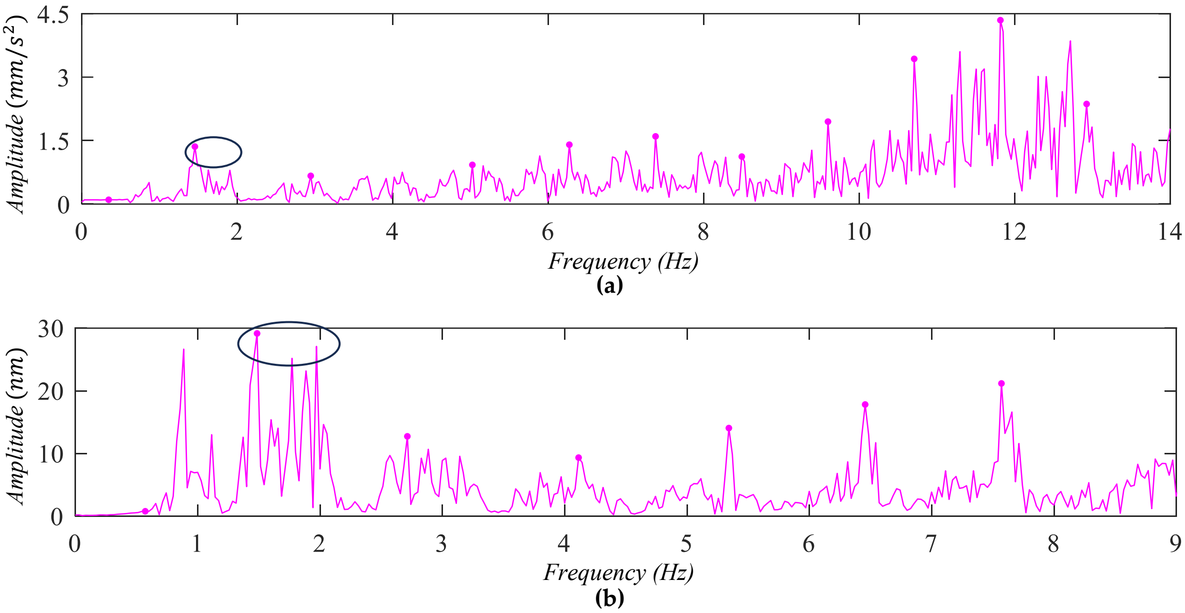

Figure S18. Comparison of the frequency spectra of sensor noise in the measured pulse signals of Subject #7 **(a)** ACC-based measurement **(b)** tactile-based measurement (Note: Due to TVSP, the peak corresponding to the natural frequency of the TCS stack spans a frequency range.)

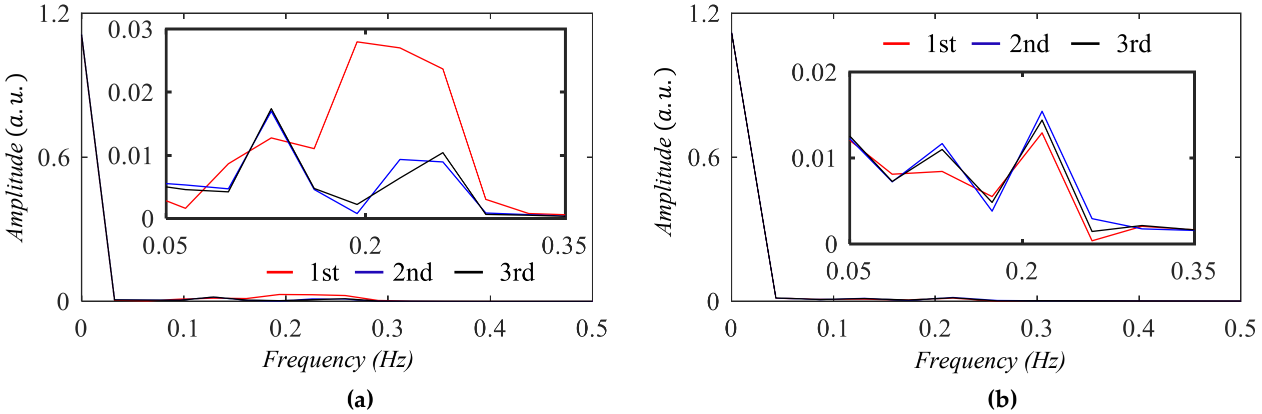

Figure S19. Comparison of the frequency spectra of the IFs of the first three harmonics of the measured pulse signals of Subject #7 **(a)** ACC-based measurement **(b)** tactile-based measurement
